# The evolutionary footprint of influenza A subtype H3N2 strains in Bangladesh: implication of vaccine strain selection

**DOI:** 10.1038/s41598-022-20179-7

**Published:** 2022-09-28

**Authors:** Sezanur Rahman, Mehedi Hasan, Md Shaheen Alam, K. M. Main Uddin, Sayra Moni, Mustafizur Rahman

**Affiliations:** 1 Virology Laboratory, Infectious Diseases Division, icddr,b, Mohakhali, 68 Shaheed Tajuddin Ahmed Sarani, Dhaka, 1212 Bangladesh; 2 Genomics Centre, icddr,b, Mohakhali, Dhaka, 1212 Bangladesh

**Keywords:** Vaccines, Influenza virus, Viral genetics

## Abstract

In February each year, World Health Organization (WHO) recommends candidate vaccine viruses for the forthcoming northern hemisphere (NH) season; however, the influenza season in the temperate zone of NH begins in October. During egg- or cell culture-propagation, the vaccine viruses become too old to confer the highest match with the latest strains, impacting vaccine effectiveness. Therefore, an alternative strategy like mRNA-based vaccine using the most recent strains should be considered. We analyzed influenza A subtype H3N2 strains circulating in NH during the last 10 years (2009–2020). Phylogenetic analysis revealed multiple clades of influenza strains circulating every season, which had substantial mismatches with WHO-recommended vaccine strains. The clustering pattern suggests that influenza A subtype H3N2 strains are not fixed to the specific geographical region but circulate globally in the same season. By analyzing 39 seasons from eight NH countries with the highest vaccine coverage, we also provide evidence that the influenza A, subtype H3N2 strains from South and Southeast Asia, including Bangladesh, had the highest genetic proximity to the NH strains. Furthermore, insilico analysis showed minimal effect on the Bangladeshi HA protein structure, indicating the stability of Bangladeshi strains. Therefore, we propose that Bangladeshi influenza strains represent genetic makeup that may better fit and serve as the most suitable candidate vaccine viruses for the forthcoming NH season.

## Introduction

In 2019, the estimated production capacity of seasonal influenza vaccines was 1.48 billion doses over 12 months^1^. However, the demand for seasonal influenza vaccines has been increasing (i.e. the USA estimated 200 million doses for 2021–2022 season), considering the health and economic impact of influenza virus with an estimated annual 5–10% infection in adults^2^ and 20–30% in children^3^. The World Health Organization (WHO) estimates that these infections result in about 3 to 5 million cases of severe illness and about 290,000 to 650,000 deaths globally^4^. However, the influenza A virus does cause not only seasonal endemic infections in humans, but also unpredictable periodic pandemics.

A recent meta-analysis calculated the influenza attack rate among unvaccinated individuals, and found that 22.5% of child < 18 years old and 10.7% of adults were influenza positive^5^. Due to influenza, the estimated average annual total economic burden was $11.1 billion in the United Stats^6^. To minimized this health and economic burden, vaccination remains the best strategy for preventing the spread of seasonal influenza. Due to rapid viral evolution, the influenza vaccine formulation requires updates each flu season; hence, the term “seasonal vaccine”^7^.

Antigenic evolution of human influenza A viruses facilitate the virus to escape pre-existing immunity through abrupt drift with structural alterations in antigenic epitopes. The segmented genome and zoonotic nature allow the exchange or reassortment of RNA segments between viruses when a host is co-infected with different influenza strains, known as antigenic shift. This may generate novel strains with enhanced pathogenicity and facilitate crossing species barriers, thereby contributing to influenza pandemics at unpredictable intervals^8^. Thus, variants of circulating or newly emerging influenza A viruses continue to trigger global health threats annually for both humans and animals.

Circulating influenza A virus is classified based on the genotype of hemagglutinin (HA) and neuraminidase (NA) proteins (18 HA and 11 NA)^9^. Among these subtypes, H1N1 pdm09 virus, a descendant of the seasonal H1N1 influenza which had circulated before 2009, is now predominant in most countries, although the proportion of H3N2 virus had increased over time^10^. Therefore, WHO and the National Influenza Centers conduct influenza virologic surveillance to monitor the spread of viruses and their continuous evolution. Each year considering previous surveillance data, an expert panel of WHO recommends the most appropriate vaccine for the next season in February and September for the Northern and Southern Hemispheres (SH), respectively. Three types of seasonal influenza vaccines are currently licensed for human use; however, most influenza vaccines are distributed as inactivated vaccines (89.6% of global production) which contain antigen from two influenza A strains (subtype H1N1 and H3N2), and including influenza B strain(s). Global production of two other vaccines, live attenuated influenza vaccine (LAIV) and recombinant influenza vaccine (RIV) was similar in 2019, 5.0% and 5.4% respectively^1^. But, over time, antigenic changes of circulating strains affects receptor preference, virulence, and vaccine effectiveness (VE)^11,12^. Therefore, VE against laboratory-confirmed influenza virus infection is rarely higher than 60% and may sometimes be 30% or less, with protection that may wane from one season to the next or within a season^13–18^.

Furthermore, the timing and duration of the influenza season vary by country and by year. Previous studies showed that new antigenic variants of influenza A subtype H3N2 appear every 3–5 years, whereas new antigenic variants of influenza A subtypr H1N1 viruses appear less frequently^19^. However, phylogenetic analyses provided evidence against the local persistence of influenza A subtype H3N2 viruses between epidemics^20^ where South and Southeast Asia (SSEA) act as the global source of the viruses^21–25^, that might spread globally^19^. In SSEA region, influenza A subtype H3N2 viruses become locally extinct between epidemics, but variation in local climates enables viruses to circulate continuously from one epidemic to another^19,21^. Therefore, we explored the evolutionary footprint of influenza A subtype H3N2 strains in Bangladesh to assess their suitability as potential vaccine candidates (referred as p-vac throughout the text).

International Centre for Diarrhoeal Disease Research, Bangladesh (icddr,b) in collaboration with the Institute of Epidemiology Disease Control and Research (IEDCR), the national influenza center, has been conducting hospital-based influenza surveillance (HBIS) since 2007 across the country^26^. Taking advantage of the existing surveillances and available genomic sequence data in EpiFlu database in GISAID.ORG, this study describes (i) the global circulating pattern of influenza A subtype H3N2 strains in contrast to the vaccine strain through phylogenetic analysis; (ii) the evolutionary pattern of the influenza A subtype H3N2 virus circulating in Bangladesh during the last decade, and finally, (iii) genetic proximity of p-vac with circulating strains of high vaccine coverage countries in the northern hemisphere (NH).

## Results

### Phylogenetic analysis of influenza H3N2 strains

**Figure 1:**
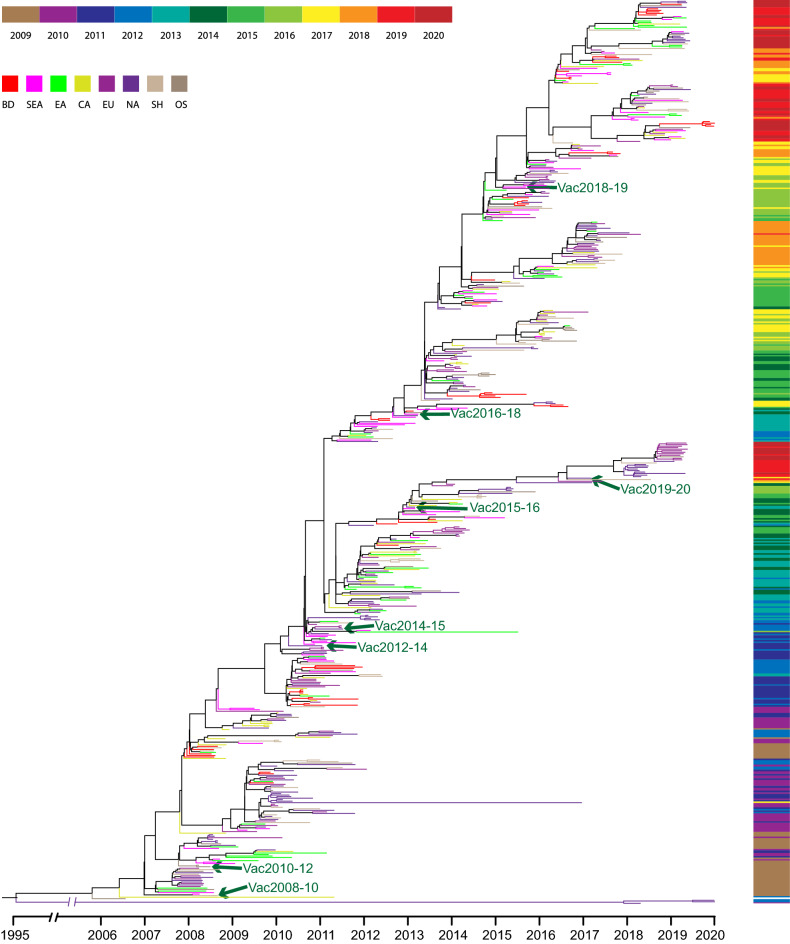
The time-scaled phylogenetic tree of HA gene from globally circulating influenza A subtype H3N2 strains, including WHO-recommended vaccine strains (indicated by green arrow). Different branch colours indicated strains location. The right colour ribbon indicates the year of sample collection.

A phylogenetic tree was constructed to observe the possible circulation pattern of influenza A subtype H3N2 over time, using HA genes of vaccine strains (recommended for the NH) and globally circulating strains (Fig. 1). Different branch colours indicate the geographical origin of the strains; i.e. red for Bangladesh and pink for South-East-Asia (SEA). The colour ribbon on the right side shows the timeline of each strain. Thus, the same colour in different positions of the ribbon specifies more than one phylogenetically distinct influenza strains circulating every season. The green arrows indicate the vaccine strains (WHO recommended) that are distantly related to the circulating strains of the respective seasons, with few exceptions. The same cluster with different branch colour suggests that influenza A subtype H3N2 strains are not fixed to the specific geographical region but circulate globally in the same season.

For understanding the relationship between the Bangladeshi dominant strains, p-vac and WHO recommended vaccines, we have constructed another phylogenetic tree using a generalized time-reversible (GTR) substitution model (Supplementary Appendix B, Fig. B1). This tree also produced similar results to MCMC tree (Fig. 1), where the WHO-recommended vaccine placed in a different clade than the most dominant Bangladeshi strains (Table 1).

**Table 1:** The clade of WHO recommended H3N2 vaccine strains and concurrent most dominant strains detected in Bangladesh.

Season	Recommended vaccine	Clade	Concurrent dominatig clade of Bangladeshi strain
2010–2011	A/Perth/16/2009	1	3C
2011–2012	A/Perth/16/2009	1	3C
2012–2013	A/Victoria/361/2011	3C	3C
2013–2014	A/Victoria/361/2011	3C	3C
2014–2015	A/Texas/50/2012	3C	3C.2a
2015–2016	A/Switzerland/9715293/2013	3C.3a	3C.2a
2016–2017	A/Hong Kong/4801/2014	3C.2a	3C.2a1b.1
2017–2018	A/Hong Kong/4801/2014	3C.2a	3C.2a1
2018–2019	A/Singapore/INFIMH-16-0019/2016	3C.2a1	3C.2a1b.1b
2019–2020	A/Kansas/14/2017	3C.3a1	3C.2a1b.2a.2

### Genetic proximity of p-vac with circulating strains

**Figure 2:**
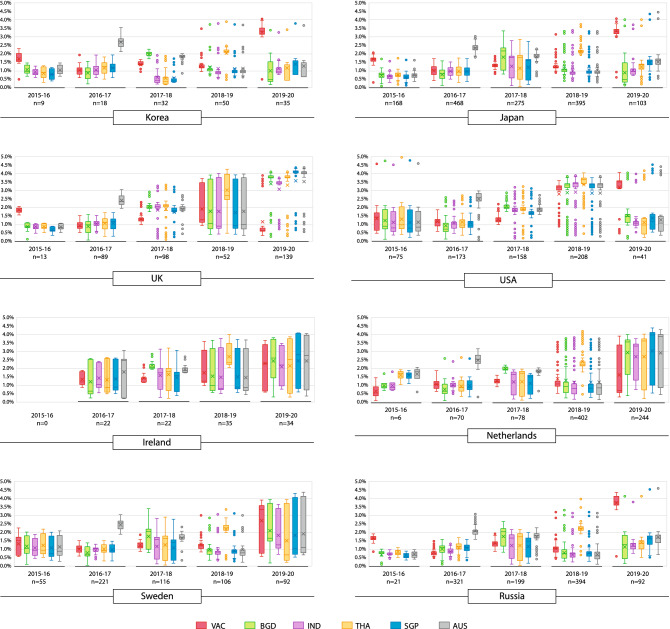
Genetic proximity of selected potential vaccine (p-vac) and WHO-recommended vaccine strain with circulating strains from consecutive seasons. Mean genetic distance (p-distance) of vaccine and p-vac strains in each season are shown separately as Box-plot graph. The number of analyzed sequences from a season were denoted with (n).

The genetic distance (p-distance) of WHO-recommended vaccine and p-vac from SSEA with NH strains (South Korea, UK, USA, Ireland, The Netherlands, Japan, Sweden and Russia) circulating during 2015–2020, are presented in Fig. 2. The box-plot analysis, which includes 39 seasons, shows that the p-vac were more closely related to NH strains circulating in the subsequent seasons (31/39, 79.5%) compared to vaccine strains (20.5%). The box-plot for Korea indicates the average p-distances of vaccine strain in red which was much higher than the p-vac in other colours; even sometimes higher than the control strain from SH in grey. A similar conclusion can be made for other countries.

**Figure 3:**
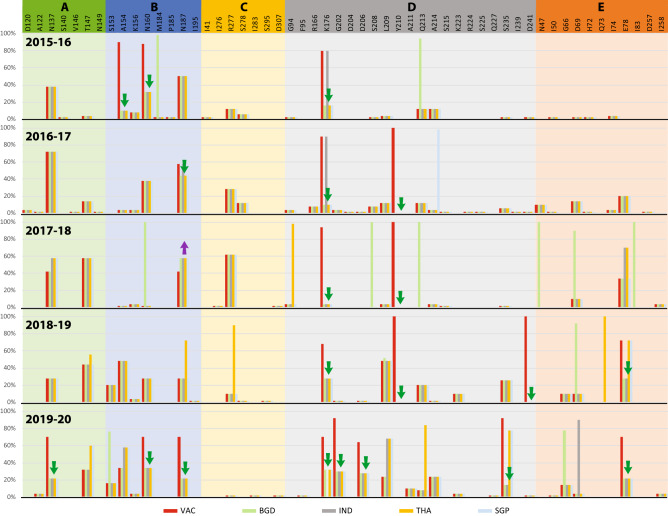
Amino acid changes in epitope site of global hemagglutinin protein (HA) sequences. The red bar indicates the percent of changes compared with the WHO-recommended vaccine, while others indicate p-vac. The green down-arrow indicates the site where p-vac (any of the four) showed higher proximity with global strains than the vaccine. The purple up-arrow indicates the site where the vaccine showed higher proximity than all p-vac.

Since the vaccine effectiveness is largely affected by epitope sites, we further compared epitope sites of vaccine strains and p-vac with globally circulating strains during 2015–2020 from Dataset-1. In most of the epitope sites, the p-vac and NH strains were closer than the vaccine strains indicated by green arrows (Fig. 3). In epitope-D, one amino acid site (K176) in 2015–2016, two (K176 and P210) during 2016–2018, three (K176, P210 and D241) in 2018–2019 and four (K176, G202, D206 and S235) in 2019–2020 were identified. In other epitopes, A154, N160 in 2015–2016; N187 in 2016–2017; E78 in 2018–2019; and E78, N137, N160 and N187 in 2019–2020, also showed higher proximity of NH strains with p-vac than vaccine except one in 2017–2018 season indicated by purple arrow (Fig. 3).

### The evolutionary trend of Bangladeshi Inf/H3N2 strains

**Figure 4:**
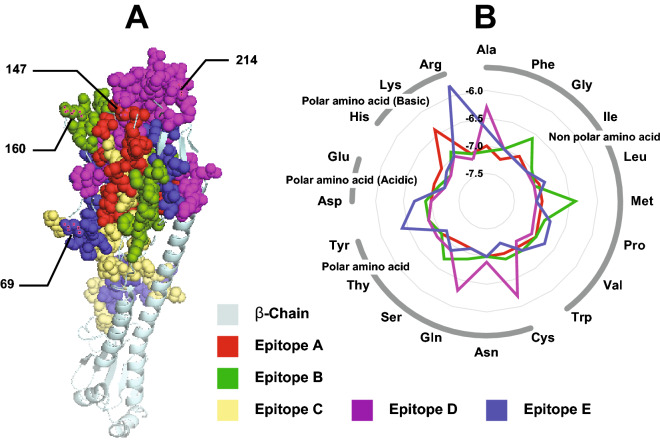
(**A**) 3D structure of hemagglutinin protein (HA) indicating diversifying epitope sites for Bangladeshi strain; (**B**) changes in host receptor binding affinity for alterations in the amino acid, at the positions of diversifying epitope sites.

We analyzed all eight gene segments of influenza A subtype H3N2 circulating in Bangladesh (n = 531) from 2009 to 2020 to observe the mutation pattern (Table 2). The highest mutation rate was found in NS (4.54 × 10^–3^ nucleotide substitutions/site/year), and the lowest rate in PA (2.37 × 10^–3^ nucleotide substitutions/site/year). The two major glycoproteins, HA and NA, showed high mutation rates as expected, 4.33 × 10^–3^ and 4.06 × 10^–3^ nucleotide substitutions/site/year, respectively. For all gene segments, the divergence ratio at non-synonymous and synonymous sites across the protein sequence was negatively selected (dN/dS < 1). However, most of the amino acids were conserved at a specific position in a protein sequence; therefore, selection pressure for each site was calculated using four different models (Table 1). The highest diversifying eight positive selection sites were identified in HA followed by six in NA, three in NS1 and PB2, two in PA, and one in MP and PB1. Among the eight diversifying HA sites, four belonged to one from each epitope site of A, B, D and E; at amino acid positions 147, 160, 214 and 69, respectively (Fig. 4A).

**Table 2:** Evolutionary rate of Bangladeshi H3N2 strains over a decade (Jan 2009–Dec 2020).

Segments (n = 531)	Evolutionary rate (95% HPD)	dN/dS^1^		Total sites^1^	Sites under diversifying positive selection (Amino acid position)			
		SLAC^2^	MEME^2^		SLAC^2^	MEME^2^	FEL^2^	FUBAR^3^
HA	4.33 × 10^–3^ (3.61–5.03)	0.269	0.238	566	(69, 160, 546)	(69, 130, 147, 151, 160, 546)	(69, 130, 160, 546)	(69, 130, 160, 188, 214, 546)
NA	4.06 × 10^–3^ (3.41–4.75)	0.278	0.247	470	(345)	(253, 345)	(144, 345)	(94, 345, 381, 469)
MP	3.81 × 10^–3^ (2.97–4.70)	0.053	0.049	252	–	–	–	(187)
NP	2.61 × 10^–3^ (2.01–3.43)	0.102	0.095	498	–	–	–	–
NS	4.54 × 10^–3^ (3.42–5.57)	0.407	0.395	230	–	(99)	–	(37, 56)
PA	2.37 × 10^–3^ (2.04–2.70)	0.11	0.102	716	–	–	(256)	(216, 256)
PB1	3.03 × 10^–3^ (2.46–3.70)	0.073	0.067	757	–	(618)	(618)	(618)
PB2	2.56 × 10^–3^ (2.49–3.21)	0.095	0.091	759	–	(299, 613)	(299, 613)	(299, 480, 613)

*HPD* highest probability density, *dN/dS* ratio of non-synonymous and synonymous sites, *SLAC* single-likelihood ancestor counting, *MEME* mixed effects model of evolution, *FEL* fixed effects likelihood, *FUBAR* fast, unconstrained Bayesian approximation.

^1^For MP and NS gene, respectively M1 and NS1 proteins were analyzed.

^2^p-value threshold of 0.1.

^3^Posterior probabilities of 0.9.

### Insilico analysis of highly diversifying epitope site

3-D models for all possible amino acid substitutions at positions 69, 147, 160, and 214 were prepared based on the highest sequence identity with templet (98 to 99), sequence similarity (0.62), coverage (0.88), and lowest resolution value (1.90 Å) determined by the X-ray diffraction method. The Ramachandran plot for all models indicates that more than 95% of the residues were in the favoured regions and only < 0.4% in the outlier region. Based on the Verify 3D software, more than 88% of the residues have an average 3D-1D score of ≥ 0.2. For all protein models, ERRAT’s overall quality factor ranges from 96 to 98. According to the ProSA-web result, all protein models were of appropriate quality and consistency with Z-scores ranging from − 8.68 to − 9.6.

To see if alterations in the amino acid, at the positions of 147, 160, 214, and 69 from A, B, D, and E epitope respectively have influenced the host receptor binding, computationally all of 80 3D prepared proteins were docked on N-acetyl-alpha-neuraminic acid-(2-6)-beta-d-galactopyranose (Fig. 4B). In position 147, substitution with Lysine showed reduced affinity while others had no significant change for epitope A. Similarly, in position 160, Glycine and Methionine; in position 214, Alanine, Cysteine and Glutamine; and in position 69, Tyrosine and Arginine showed reduced affinity; while other substitutions showed no significant changes for B, D, and E epitope respectively (Fig. 4B).

## Discussion

Our study revealed the global circulating pattern of influenza A subtype H3N2 strains over time through phylogenetic analysis, where two or more distinct influenza strains circulate simultaneously in every season were identified. Additionally, vaccine strains were misplaced from the main branch of circulating strains of the respective seasons. Therefore, we considered the latest influenza A subtype H3N2 strains from South and South-East Asian (SSEA) regions as potential vaccine candidates (p-vac). The comparative genetic proximity and amino acid analysis of the HA protein epitope showed that p-vac from SSEA were closely related to the NH strains than the vaccine.

Bangladesh has been providing genomic data through existing influenza surveillance, and here, we analyzed the evolutionary pattern of Bangladeshi strains from the last decade. A major rate difference between those segments expressed on the surface of the virus (high rates) and those that only have internal functions (low rates) was observed. However, a modest mutation rate of HA gene was observed in Bangladesh influenza A subtype H3N2 (Supplemenatry Appendix Table A2). But, this rate was higher than influenza A subtype H1N1 and influenza B in humans^27,28^. Due to a higher mutation rate, subtype H3N2 infected individuals might remain immunologically naïve for recurrent subtype H3N2 infections^19^. However, our insilico analysis showed no major affinity changes for amino acid substitution in the highly diversifying position of epitope sites of Bangladeshi strains, except few exceptions (Fig. 4). Therefore, selecting Bangladeshi strains as the global vaccine is very promising.

The degree of similarity between vaccines and circulating strains are affected by the heterogeneity of the influenza A subtype H3N2 virus, resulting in a modest VE each season^29^. In this study, the phylogenetic analysis showed a similar result in the heterogeneity of global influenza A subtype H3N2 and vaccine strains. For instance, the vaccine of the 2019–2020 season made a close cluster with north American 2018 strains while globally circulated 2019–2020 strains were in a distinctive cluster, which might result in low (39%) VE in the USA for season 2019–2020^30^. Such disparity between circulating viruses and the vaccine strain could have resulted in low immunity of populations, leading to reduced VE previously reported globally^31–34^. On the other hand, the overall VE was estimated below 40%, even after genetic proximity with circulating strains^34–36^, due to the co-circulation of different strains and/or sudden decline of a dominant strain^37^. Furthermore, the H3N2 vaccines undergo mutation during passage in eggs in 2012–2013, 2013–2014, 2014–2015, 2015–2016, 2017–2018, and 2018–2019 seasons^37,38^. The fast and unpredicted evolution is the main reason for these scenarios.

However, increased surveillance revealed that the SSEA region plays an important role in influenza virus evolution and seeding epidemics worldwide^19^. While the influenza season starts later in this region than other NH countries^19^; therefore, the WHO failed to consider the latest strains during vaccine recommendation in February for the upcoming season. Here, we tried to find potential vaccine (p-vac) strains with the highest genetic proximity to circulating strains of subsequent flu season from the latest SSEA strains. Every season constant higher genetic proximity with circulating strains was observed for p-vac. Therefore, the upcoming NH’s seasonal influenza vaccine should be recommended after considering the most recent strain from SSEA.

The study had several limitations. First, selecting the early appeared strains region as p-vac, could be a selesction bias due to the fact that the selected p-vac starin would no longer the dominant starins in that season. Second, the genetic distance between WHO-recommended strains and p-vac from SSEA was missing during 2009–2015 because of limited data on consecutive seasons available in GISAID database. Finally, we only focused on influenza A subtype H3N2 strain and NH region. Whether this method is applicable for influenza A subtype H1N1 strain and SH region, needs further investigation to illustrate the comprehensive global scenario.

Our study concluded that influenza strains from SSEA are closer to the time of intended vaccine use and had the highest genetic proximity to strains of the next season. By selecting these strains as vaccine candidates will help reducing the window of opportunity for the emergence of new antigenic variants in the human population. Although the vaccine candidates identified by this approach showed better genetic proximity to circulating strains of subsequent flu season, further study including global strains with immunological assay data is required to identify the best vaccine candidates. A highly sensitive pseudovirus-based assay for neutralization antibodies against the influenza virus could be a potential tool for in-vitro validation and evaluation of p-vac^39–41^. In addition, considering the most recent strain will delay the production of egg-based influenza vaccines, which can be overcome by using advanced mRNA based technology^42^ as an alternative for future influenza vaccines. Our findings provide new insight into the influenza vaccine selection and production process for the policymaker.

## Methods

### Time-scaled phylogenies of globally circulating strains

Neutralizing antibodies or immunity are generated against HA protein^43^. Therefore, only HA gene was considered for further analysis and Dataset-1 was prepared, including 596 global HA sequences. In brief, from 2009 to 2020 at least 5 strains were randomly selected for each year from Bangladesh, East Asia (China/Korea/Japan), Southeast Asia (Indonesia/Malaysia/Philippines/Singapore/Thailand/Vietnam), Central Asia (India/Kazakhstan/Uzbekistan), Europian Union (Germany/Greece/Spain/Sweden), United Kingdom, North America (Canada/USA), Oceania (Australia/New Zealand), and Southern Hemisphere (Namibia/ Madagascar/South Africa/Argentina/Brazil/Chile/Peru) (Supplementary Appendix D: Dataset-1). All the influenza A subtype H3N2 vaccine strains recommended for the 2008–2009 to 2019–2020 seasons in the NH were also included in this dataset.

Bayesian inference was implemented through a Markov chain Monte Carlo (MCMC) framework in BEAST v2.6.3^44^ to prepare the evolutionary tree. We employed GTR for each dataset and a coalescent Bayesian skyline tree prior and strict molecular clock model. MCMC chains were run for 5 million steps, and TreeAnnotator program v2.6.0 was used to summarise the obtaining file as a maximum clade credibility (MCC) tree. Finally, the tree with the maximum product of posterior probabilities after a 10% burn-in was visualized using FigTree v1.4.4.

### Selection and comparison of potential vaccine (p-vac) with country wise data

Countries between equator and tropic of cancer from the world map were selected for potential vaccine (p-vac) strain selection as influenza A subtype H3N2 spread globally from SSEA each year^19^. Only four countries (Bangladesh, India, Thailand, and Singapore) had sequences of the last five consecutive seasons (2015–2020) from this region. Australia from the SH was also included as an outgroup for identifying p-vac. The first identified sequence (based on collection date in the database) was selected as p-vac from each season, but no later than the 2nd of June (Supplementary Appendix D: Dataset-2). All selected p-vac were compared with sequence data from the top five influenza vaccination countries in the NH; Korea, UK, USA, Ireland, Netherlands, Japan, and Sweden^45^. Although vaccine coverage data was unavailable for Russia, sequences data were included as Russia covered a large geographical region. A separate dataset (Supplementary Appendix D: Dataset-3) having all sequences from 2015–2016 to 2019–2020 seasons of each country was prepared. The average genetic distance (p-distance) with all five p-vac and WHO recommended vaccine strains of each concurrent season was calculated using MEGA X (Version 10.0.5)^46^.

### Evolutionary rate and selection pressure measurement for Bangladeshi strain

All gene segments of influenza A subtype H3N2 strains (n = 531) from 2009 to 2020 were retrieved (Supplementary Appendix D: Dataset-4) from the EpiFlu database of the Global Initiative on Sharing All Influenza Data (GISAID) website (www.gisaid.org) for analysis of evolutionary dynamics. The MUSCLE programme^47^ was used for multiple sequence alignment. To calculate the evolutionary rate (nucleotide substitutions/site/year), an uncorrelated relaxed clock log-normal molecular clock model with coalescent Bayesian skyline prior was used through a Markov Chain Monte Carlo (MCMC) framework in BEAST (v2.6.3)^44^. MCMC chains were run for 100 million steps, sampling every 10,000 steps from the posterior distribution to ensure adequate mixing of model parameters. The best-fit nucleotide substitution model was identified according to the Akaike information criterion, Bayesian information criterion, and performance-based decision theory method with 11 (88 candidate models) substitution schemes in jModelTest v2.1.10^48^. GTR model was used for each gene, and the evolutionary rate was evaluated using Tracer v1.7.1. Finally, all the genes were subjected to selection pressures measurement using the Datamonkey server^49^ by calculating the ratio of non-synonymous (dN) and synonymous (dS) nucleotide substitutions per site (dN/dS).

### Insilico epitope analysis of HA protein

For epitope and other Insilico analysis of HA protein from NCBI GenBank references strain A/New York/392/2004 subtype H3N2 (accession# YP_308839) was used as a representative sequence for exploring the changes of binding affinity to its human receptor. Each highly diversifying positive selection site (amino acid positions 147, 160, 214, and 69) were manually substituted with corresponding amino acid and prepared a dataset of 80 target sequences for homology modelling using the SWISS-MODEL server^50^. The SWISS-MODEL template library (SMTL version 2022-01-05, PDB release 2021-12-31) was explored to search for the best templates for the target sequences. The SWISS PDB viewer optimized the geometry by energy reduction on the created 3D models. The quality of the projected 3D models was examined using Duke University’s Molprobity web server^51^, Verify 3D, and ERRAT. For the investigation of structural flaws, plotting of residue scores, and generating Z scores, ProSA^52^ was used. For ligand preparation and molecular docking to determine Binding Affinity, N-acetyl-alpha-neuraminic acid-(2-6)-beta-d-galactopyranose was extracted from the PDB:2WRE as a ligand molecule. Geometry optimization of ligand molecule was conducted using MMFF94 force field in Avogadro^53^. Molecular docking of individual 3D protein models with optimized ligand was performed by AutoDock wizard in PyRx (version 0.8) virtual screening tool^54^ to find out binding affinities. Their interactions were visualized and analyzed using Discovery Studio Visualizer v20.1.0.19295, Chimera 1.13rc, and Pymol (The EduPyMOL Molecular Graphics System, Version 1.7.4.5 Schrödinger, LLC).

## Supplementary Information

Supplementary Information.

## Data Availability

The data supporting the findings of this study are available with this article as a Supplementary Appendix file.
