# Supplementary material for: The evolutionary footprint of influenza A subtype H3N2 strains in Bangladesh: implication of vaccine strain selection
Source: Sci Rep. 2022 Sep 28;12:16186. doi: 10.1038/s41598-022-20179-7 (PMC9519982; doi:10.1038/s41598-022-20179-7)
Supplement: Supplementary file 1 — Supplementary Information. [file 41598_2022_20179_MOESM1_ESM.pdf]

Appendix of

The evolutionary footprint of Influenza A subtype H3N2 strains in Bangladesh: implication of vaccine strain selection

Sezanur Rahman[1], Mehedi Hasan[1], Md Shaheen Alam[1], K M Main Uddin[1], Sayra Moni[1], Mustafizur Rahman[1,2,*]

[1]Virology Laboratory, Infectious Diseases Division, icddr,b, Mohakhali, Dhaka-1212, Bangladesh

[2]Genome Centre, icddr,b, Mohakhali, Dhaka-1212, Bangladesh

*Correspondence to: Mustafizur Rahman, 68 Shaheed Tajuddin Ahmed Sarani, Dhaka 1212, Bangladesh; mustafizur@icddrb.org; Telephone number: +880 2 9840523-32

Table of contents

Heading	Topics	Page
Appendix A	Evidence before this study	02-02
	Table A1: MEDLINE (PubMed) search strategy (11.11.2021)	02
	Table A2: Previously reported substitution rate	02
Appendix B	Supplementary data	02-02
	Table B1: Evolutionary Rate of HA gene from whom (South and Southeast Asian country) potential vaccine (p-vac) was selected. (Jan 2015-Dec 2020)	02
	Figure B1: Mximum Likelihood tree of BD strains and vaccine strains from 2009 to 2020.	03
Appendix C	Insilico analysis and validation	04-07
	Table C1: Binding Affinity of epitopes after amino acid substitutions	04
	Table C2: Structure validations, Result from MolProbidity Serve	06
Appendix D	Data in open access server/ Accession numbers	08-43
	Dataset 1: Accession numbers of global HA sequences (n= 596) used for Time-scaled phylogenies.	08
	Dataset 2: Selected potential vaccine candidate (p-vac) from each season.	14
	Dataset 3: Sequences from 2015-16 to 2019-20 seasons	
	Dataset 4: Accession numbers of Bangladeshi strains (n=531) collected from 2009 to 2020	15
	Dataset 5: Accession numbers of South-South East Asian strains used to track evolutionary rate for HA gene (Jan 2015-Dec 2020)	37
References		44

Appendix A: Evidence before this study

Table A1: MEDLINE (PubMed) search strategy (11.11.2021)

Search number	Search Details	Results
1	(H3N2[Title]) AND (influenza[Title])	1,494
2	((phylogen*) OR (evolution*)) OR (nucleotide substitution*)	854,286
3	(search 1) AND (search 2)	412
4	(search 1) AND (search 2) Filters with_ (i) SPECIES: Humans; (ii) PUBLICATION DATE: 5 years; (iii) ARTICLE TYPE: Journal Article; (iv) LANGUAGE: English	82

Table A2: Previously reported substitution rate

Country/ Region	Time	Substitution rate (nucleotide substitutions/ site/year)	Reference
Australia	2003-2017	6.44×10^{-3}	[1]
Indonesia	2008-2010	3.27×10^{-3}	[2]
Korea	2009-2012	4.84×10^{-3}	[3]
Switzerland	2016/2017	2.90×10^{-3}	[4]
Middle East and North Africa	2009-2017	1.47×10^{-3}	[5]
Global (n=254)	1968-2015	0.50×10^{-3}	[6]
Kenya	2007-2013	4.17×10^{-3}	[7]
Global (n=284)	1968-2011	3.99×10^{-3}	[8]

Appendix B: Supplementary data

Only complete HA gene sequences available on GISAID (**List of accession number in Dataset 5**) were considered for evolutionary rate analysis (Table B1). An uncorrelated relaxed clock log-normal molecular clock model with coalescent Bayesian skyline prior was used through a Markov Chain Monte Carlo (MCMC) framework in BEAST (v2.6.3). MCMC chains were run for 10 million steps, sampling every 10,000 steps from the posterior distribution to ensure adequate mixing of model parameters.

Table B1: Evolutionary Rate of HA gene (Jan 2015-Dec 2020)

Country	Number of sequences	Evolutionary Rate (95%HPD)	dN/dS
Bangladesh	700	4.97×10-3 (4.23-5.73)	0.81
India	139	8.29×10-3 (6.54-9.99)	0.76
Singapore	680	4.31×10-3 (3.74-5.13)	0.81
Thailand	502	4.25×10-3 (3.78-4.70)	0.75

We have constructed another phylogenetic tree using a generalized time-reversible (GTR) substitution model.

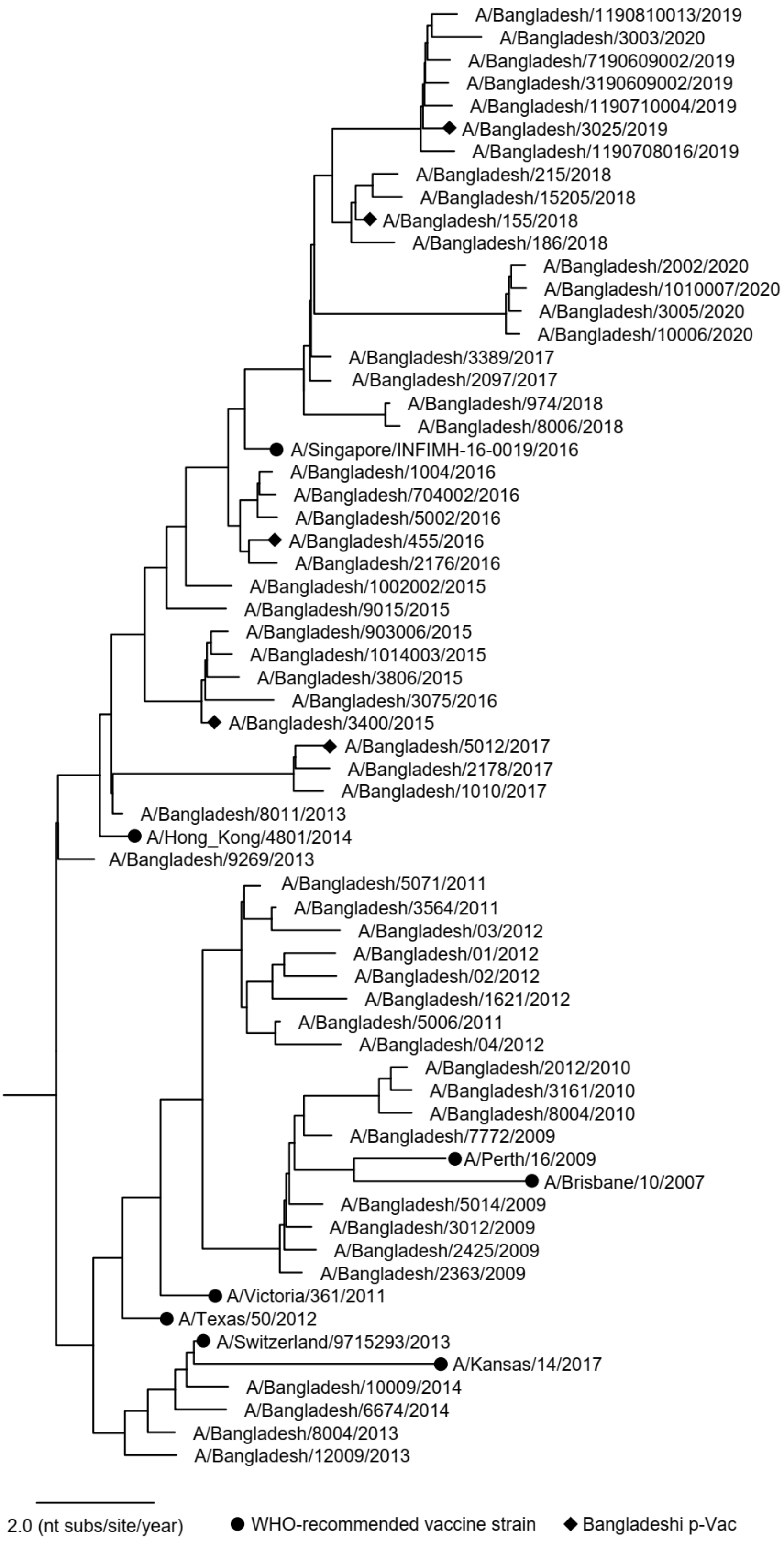

Figure B1: Mximum Likelihood tree of BD strains and vaccine strains from 2009 to 2020.

Appendix C: Insilco analysis and validation.

Table C1: Binding Affinity of epitopes after amino acid substitutions

	Amino acid substitutions	1st Pose	2nd Pose	3rd Pose	4th Pose	5th Pose	6th Pose	7th Pose	8th Pose	9th Pose
Epitope A	147 A	-7	-6.8	-6.6	-6.6	-6.6	-6.4	-6.4	-6.3	-6.3
	147 F	-7.2	-7	-6.9	-6.9	-6.9	-6.8	-6.8	-6.5	-6.4
	147 G	-7	-6.9	-6.7	-6.5	-6.5	-6.4	-6.3	-6.2	-6
	147 I	-7.1	-6.9	-6.8	-6.7	-6.7	-6.7	-6.6	-6.6	-6.5
	147 L	-7	-7	-6.9	-6.7	-6.6	-6.5	-6.5	-6.5	-6.4
	147 M	-7	-6.8	-6.8	-6.7	-6.7	-6.6	-6.5	-6.5	-6.1
	147 P	-7	-6.9	-6.8	-6.2	-6.2	-6.2	-6.1	-6	-6
	147 V	-6.9	-6.9	-6.7	-6.6	-6.6	-6.5	-6.4	-6.4	-6.4
	147 W	-7	-6.7	-6.6	-6.6	-6.6	-6.5	-6.5	-6.3	-6.3
	147 C	-7	-6.9	-6.9	-6.8	-6.7	-6.6	-6.5	-6.5	-6.4
	147 N	-7	-6.9	-6.9	-6.7	-6.6	-6.4	-6.4	-6.4	-6.3
	147 Q	-7.1	-7.1	-6.9	-6.6	-6.5	-6.3	-6.3	-6.2	-6.1
	147 S	-7.1	-7.1	-7	-6.9	-6.9	-6.8	-6.8	-6.6	-6.6
	147 T	-7	-6.9	-6.8	-6.8	-6.8	-6.7	-6.7	-6.6	-6.5
	147 Y	-6.9	-6.9	-6.7	-6.7	-6.5	-6.4	-6.3	-6.2	-6.2
	147 D	-7	-6.7	-6.7	-6.7	-6.4	-6.4	-6.4	-6.2	-6.2
	147 E	-7	-7	-6.9	-6.8	-6.8	-6.7	-6.6	-6.6	-6.4
	147 H	-7	-6.7	-6.6	-6.6	-6.5	-6.3	-6.3	-6.2	-6.1
	147 K	-6.4	-6.2	-6.1	-5.8	-5.7	-5.6	-5.6	-5.5	-5.5
	147 R	-7.1	-7.1	-6.9	-6.8	-6.7	-6.7	-6.7	-6.6	-6.6
Epitope B	160 A	-7.1	-7	-7	-6.7	-6.7	-6.7	-6.7	-6.5	-6.5
	160 F	-7	-6.9	-6.9	-6.8	-6.7	-6.7	-6.4	-6.3	-6.3
	160 G	-6.6	-6.6	-6.6	-6.5	-6.3	-6.2	-6.1	-6	-6
	160 I	-7	-6.9	-6.9	-6.9	-6.7	-6.6	-6.5	-6.5	-6.4
	160 L	-7.1	-7	-6.9	-6.7	-6.6	-6.6	-6.5	-6.4	-6.2
	160 M	-6.4	-6.4	-6.3	-6.3	-6.2	-6.2	-6.2	-6.1	-6
	160 P	-6.9	-6.8	-6.8	-6.6	-6.5	-6.2	-6.1	-6.1	-5.9
	160 V	-6.9	-6.8	-6.8	-6.6	-6.5	-6.5	-6.4	-6.3	-6.3
	160 W	-6.9	-6.8	-6.7	-6.7	-6.6	-6.3	-6.2	-6.2	-6.1
	160 C	-6.9	-6.9	-6.7	-6.7	-6.7	-6.5	-6.5	-6.4	-6.4
	160 N	-7	-6.8	-6.8	-6.7	-6.6	-6.4	-6.3	-6.3	-6.2
	160 Q	-6.9	-6.6	-6.6	-6.5	-6.4	-6.3	-6.2	-6.2	-6.2
	160 S	-6.7	-6.4	-6.4	-6.2	-6.2	-6.1	-6.1	-6.1	-6
	160 T	-7	-6.7	-6.4	-6.3	-6.2	-6.2	-6.1	-6.1	-6.1
	160 Y	-6.9	-6.8	-6.8	-6.7	-6.6	-6.5	-6.5	-6.5	-6.4
	160 D	-6.9	-6.7	-6.6	-6.6	-6.6	-6.3	-6.2	-6.1	-6.1
	160 E	-7.1	-7.1	-6.9	-6.9	-6.8	-6.7	-6.7	-6.6	-6.6
	160 H	-7.2	-7	-6.9	-6.9	-6.6	-6.5	-6.4	-6.4	-6.4
	160 K	-6.9	-6.8	-6.8	-6.5	-6.5	-6.4	-6.3	-6.3	-6.2
	160 R	-7.1	-7	-6.9	-6.9	-6.9	-6.8	-6.7	-6.7	-6.4

	Amino acid substitutions	1st Pose	2nd Pose	3rd Pose	4th Pose	5th Pose	6th Pose	7th Pose	8th Pose	9th Pose
Epitope D	214 A	-6.3	-6.1	-5.9	-5.9	-5.6	-5.6	-5.6	-5.6	-5.5
	214 F	-7	-6.9	-6.9	-6.9	-6.9	-6.7	-6.7	-6.7	-6.6
	214 G	-7.1	-6.9	-6.9	-6.8	-6.8	-6.7	-6.6	-6.5	-6.5
	214 I	-7.1	-7	-7	-6.9	-6.8	-6.6	-6.5	-6.5	-6.5
	214 L	-7.1	-6.9	-6.8	-6.5	-6.3	-6.3	-6.2	-6.2	-6.1
	214 M	-7.1	-6.9	-6.9	-6.9	-6.8	-6.8	-6.7	-6.6	-6.5
	214 P	-7.1	-7	-7	-6.8	-6.6	-6.6	-6.6	-6.4	-6.4
	214 V	-7	-6.9	-6.7	-6.7	-6.6	-6.6	-6.5	-6.4	-6.3
	214 W	-7.1	-7	-6.9	-6.8	-6.7	-6.6	-6.6	-6.5	-6.4
	214 C	-6.2	-6.2	-6.2	-6	-5.9	-5.9	-5.7	-5.7	-5.6
	214 N	-6.9	-6.9	-6.8	-6.7	-6.5	-6.4	-6.4	-6.4	-6.3
	214 Q	-6.3	-6.2	-5.9	-5.8	-5.8	-5.6	-5.5	-5.4	-5.3
	214 S	-6.9	-6.9	-6.9	-6.9	-6.6	-6.6	-6.5	-6.4	-6.4
	214 T	-6.9	-6.7	-6.7	-6.5	-6.5	-6.5	-6.3	-6.2	-6.2
	214 Y	-6.9	-6.8	-6.7	-6.7	-6.6	-6.4	-6.3	-6.2	-6.2
	214 D	-7	-6.9	-6.7	-6.4	-6.4	-6.3	-6.2	-6.2	-6.2
	214 E	-7.2	-6.9	-6.8	-6.8	-6.7	-6.6	-6.5	-6.3	-6.3
	214 H	-7.2	-7	-6.9	-6.8	-6.6	-6.6	-6.5	-6.5	-6.5
	214 K	-7	-6.9	-6.9	-6.9	-6.8	-6.7	-6.7	-6.7	-6.3
	214 R	-7.2	-7.1	-7	-6.9	-6.9	-6.8	-6.8	-6.8	-6.7
Epitope E	69 A	-6.7	-6.4	-6.3	-6.3	-6.3	-6.1	-6.1	-6.1	-6.1
	69 F	-7	-7	-6.9	-6.7	-6.6	-6.5	-6.5	-6.3	-6.2
	69 G	-7.1	-7	-6.8	-6.8	-6.7	-6.6	-6.4	-6.4	-6.4
	69 I	-7.1	-7	-6.9	-6.9	-6.8	-6.5	-6.5	-6.4	-6.2
	69 L	-6.9	-6.9	-6.9	-6.8	-6.7	-6.5	-6.5	-6.5	-6.5
	69 M	-7.1	-6.9	-6.8	-6.7	-6.6	-6.6	-6.5	-6.5	-6.5
	69 P	-6.8	-6.6	-6.6	-6.4	-6.4	-6.2	-6.1	-6.1	-6
	69 V	-6.7	-6.7	-6.5	-6.5	-6.5	-6.4	-6.3	-6.3	-6.2
	69 W	-6.9	-6.9	-6.8	-6.8	-6.7	-6.7	-6.5	-6.3	-6.3
	69 C	-7.2	-6.9	-6.9	-6.9	-6.9	-6.8	-6.8	-6.5	-6.4
	69 N	-7	-6.9	-6.8	-6.8	-6.6	-6.5	-6.5	-6.5	-6.4
	69 Q	-7.1	-6.9	-6.8	-6.5	-6.3	-6.3	-6.3	-6.2	-6.2
	69 S	-6.9	-6.7	-6.6	-6.4	-6.3	-6.3	-6.2	-6.1	-6.1
	69 T	-7.1	-6.8	-6.8	-6.7	-6.5	-6.5	-6.4	-6.3	-6.3
	69 Y	-6.4	-6.3	-6.1	-6	-5.9	-5.8	-5.6	-5.6	-5.4
	69 D	-6.7	-6.7	-6.6	-6.5	-6.5	-6.4	-6.1	-6	-6
	69 E	-7.2	-7	-7	-7	-6.8	-6.8	-6.8	-6.7	-6.7
	69 H	-7.1	-7	-6.6	-6.6	-6.5	-6.5	-6.5	-6.4	-6.4
	69 K	-7	-6.8	-6.8	-6.3	-6.3	-6.1	-6.1	-6.1	-6.1
	69 R	-5.8	-5.7	-5.6	-5.5	-5.5	-5.3	-5.3	-5.3	-5.3

Table C2: Structure validations, Result from MolProbidity Serve

Epitope	Protien with amino acid substitutions	MolProbity Score	Clash Score	Ramachandon Plot		Poor Rotamers	C-Beta Deviations	Bad Bonds	Bad Angles	Verify 3D %of the residues (averaged 3D-1D score)	ERRAT	ProSA-Web (Z-Score)
				Favored	Outliers							
Epitope A	147 A	0.9	0.4	1.0	0.0	0.0	0.0	0.0	0.0	88.9% (>= 0.2)	97.9	-8.7
	147 F	0.9	0.5	1.0	0.0	0.0	0.0	0.0	0.0	88.9% (>= 0.2)	97.9	-8.7
	147 G	0.9	0.5	1.0	0.0	0.0	0.0	0.0	0.0	88.9% (>= 0.2)	97.9	-8.8
	147 I	0.9	0.5	1.0	0.0	0.0	0.0	0.0	0.0	88.9% (>= 0.2)	97.9	-8.7
	147 L	0.9	0.5	1.0	0.0	0.0	0.0	0.0	0.0	88.9% (>= 0.2)	97.9	-8.7
	147 M	0.9	0.5	1.0	0.0	0.0	0.0	0.0	0.0	88.9% (>= 0.2)	97.9	-8.7
	147 P	0.9	0.5	1.0	0.0	0.0	0.0	0.0	0.0	88.9% (>= 0.2)	97.7	-8.8
	147 V	0.9	0.5	1.0	0.0	0.0	0.0	0.0	0.0	88.9% (>= 0.2)	97.9	-8.7
	147 W	0.9	0.5	1.0	0.0	0.0	0.0	0.0	0.0	88.9% (>= 0.2)	97.9	-8.7
	147 C	0.9	0.5	1.0	0.0	0.0	0.0	0.0	0.0	88.9% (>= 0.2)	97.9	-8.7
	147 N	0.9	0.5	1.0	0.0	0.0	0.0	0.0	0.0	88.9% (>= 0.2)	97.9	-8.8
	147 Q	0.9	0.5	1.0	0.0	0.0	0.0	0.0	0.0	88.9% (>= 0.2)	97.9	-8.8
	147 S	0.9	0.5	1.0	0.0	0.0	0.0	0.0	0.0	88.9% (>= 0.2)	97.9	-8.8
	147 T	0.9	0.5	1.0	0.0	0.0	0.0	0.0	0.0	88.9% (>= 0.2)	97.9	-8.7
	147 Y	0.9	0.5	1.0	0.0	0.0	0.0	0.0	0.0	88.9% (>= 0.2)	97.9	-8.7
	147 D	0.9	0.5	1.0	0.0	0.0	0.0	0.0	0.0	88.9% (>= 0.2)	97.9	-8.8
	147 E	0.9	0.5	1.0	0.0	0.0	0.0	0.0	0.0	88.9% (>= 0.2)	97.9	-8.8
	147 H	0.9	0.5	1.0	0.0	0.0	0.0	0.0	0.0	88.9% (>= 0.2)	97.9	-8.8
	147 K	0.9	0.5	1.0	0.0	0.0	0.0	0.0	0.0	88.9% (>= 0.2)	97.9	-8.8
	147 R	0.9	0.5	1.0	0.0	0.0	0.0	0.0	0.0	88.9% (>= 0.2)	97.9	-8.8
Epitope B	160 A	1.0	0.5	1.0	0.0	0.0	0.0	0.0	0.0	90.2% (>= 0.2)	97.7	-9.6
	160 F	1.1	1.1	1.0	0.0	0.0	0.0	0.0	0.0	89.6% (>= 0.2)	96.7	-9.0
	160 G	1.1	1.1	1.0	0.0	0.0	0.0	0.0	0.0	89.6% (>= 0.2)	96.7	-9.1
	160 I	1.1	1.1	1.0	0.0	0.0	0.0	0.0	0.0	89.6% (>= 0.2)	96.7	-9.0
	160 L	1.1	1.1	1.0	0.0	0.0	0.0	0.0	0.0	89.6% (>= 0.2)	96.7	-9.0
	160 M	1.1	1.1	1.0	0.0	0.0	0.0	0.0	0.0	89.6% (>= 0.2)	96.7	-9.0
	160 P	1.1	1.1	1.0	0.0	0.0	0.0	0.0	0.0	90.0% (>= 0.2)	96.7	-9.0
	160 V	1.1	1.1	1.0	0.0	0.0	0.0	0.0	0.0	89.6% (>= 0.2)	96.7	-9.0
	160 W	1.1	1.1	1.0	0.0	0.0	0.0	0.0	0.0	89.6% (>= 0.2)	96.7	-9.0
	160 C	1.1	1.1	1.0	0.0	0.0	0.0	0.0	0.0	89.6% (>= 0.2)	96.7	-8.9
	160 N	1.1	1.1	1.0	0.0	0.0	0.0	0.0	0.0	89.6% (>= 0.2)	96.7	-9.1
	160 Q	1.1	1.1	1.0	0.0	0.0	0.0	0.0	0.0	89.6% (>= 0.2)	96.7	-9.1
	160 S	1.0	0.5	1.0	0.0	0.0	0.0	0.0	0.0	90.2% (>= 0.2)	97.7	-9.0
	160 T	1.1	1.1	1.0	0.0	0.0	0.0	0.0	0.0	89.6% (>= 0.2)	96.7	-9.0
	160 Y	1.1	1.1	1.0	0.0	0.0	0.0	0.0	0.0	89.6% (>= 0.2)	96.7	-9.0
	160 D	1.1	1.1	1.0	0.0	0.0	0.0	0.0	0.0	89.6% (>= 0.2)	96.7	-9.0
	160 E	1.1	1.1	1.0	0.0	0.0	0.0	0.0	0.0	89.6% (>= 0.2)	96.7	-9.1
	160 H	1.1	1.1	1.0	0.0	0.0	0.0	0.0	0.0	89.6% (>= 0.2)	96.7	-9.0
	160 K	1.0	0.5	1.0	0.0	0.0	0.0	0.0	0.0	90.2% (>= 0.2)	97.7	-9.1
	160 R	1.0	0.5	1.0	0.0	0.0	0.0	0.0	0.0	90.2% (>= 0.2)	97.7	-9.0

Epitope	Protien with amino acid substitutions	MolProbity Score	Clash Score	Ramachandon Plot		Poor Rotamers	C-Beta Deviations	Bad Bonds	Bad Angles	Verify 3D %of the residues (averaged 3D-1D score)	ERRAT	ProSA-Web (Z-Score)
				Favored	Outliers							
Epitope D	214 A	0.9	0.5	1.0	0.0	0.0	0.0	0.0	0.0	88.9% (>= 0.2)	98.5	-8.8
	214 F	0.9	0.5	1.0	0.0	0.0	0.0	0.0	0.0	87.7% (>= 0.2)	97.9	-8.8
	214 G	0.9	0.5	1.0	0.0	0.0	0.0	0.0	0.0	88.9% (>= 0.2)	97.9	-8.8
	214 I	0.9	0.5	1.0	0.0	0.0	0.0	0.0	0.0	87.7% (>= 0.2)	97.9	-8.8
	214 L	0.9	0.5	1.0	0.0	0.0	0.0	0.0	0.0	88.1% (>= 0.2)	97.9	-8.8
	214 M	0.9	0.5	1.0	0.0	0.0	0.0	0.0	0.0	88.1% (>= 0.2)	97.9	-8.8
	214 P	0.9	0.5	1.0	0.0	0.0	0.0	0.0	0.0	88.9% (>= 0.2)	97.9	-8.8
	214 V	0.9	0.5	1.0	0.0	0.0	0.0	0.0	0.0	88.5% (>= 0.2)	97.9	-8.7
	214 W	0.9	0.5	1.0	0.0	0.0	0.0	0.0	0.0	88.7% (>= 0.2)	97.9	-8.8
	214 C	0.9	0.5	1.0	0.0	0.0	0.0	0.0	0.0	88.9% (>= 0.2)	97.9	-8.8
	214 N	0.9	0.5	1.0	0.0	0.0	0.0	0.0	0.0	88.9% (>= 0.2)	97.9	-8.8
	214 Q	0.9	0.5	1.0	0.0	0.0	0.0	0.0	0.0	88.7% (>= 0.2)	97.7	-8.8
	214 S	0.9	0.5	1.0	0.0	0.0	0.0	0.0	0.0	88.9% (>= 0.2)	97.9	-8.8
	214 T	0.9	0.5	1.0	0.0	0.0	0.0	0.0	0.0	88.9% (>= 0.2)	97.9	-8.8
	214 Y	0.9	0.5	1.0	0.0	0.0	0.0	0.0	0.0	88.3% (>= 0.2)	97.9	-8.8
	214 D	0.9	0.5	1.0	0.0	0.0	0.0	0.0	0.0	88.9% (>= 0.2)	97.9	-8.8
	214 E	0.9	0.5	1.0	0.0	0.0	0.0	0.0	0.0	88.7% (>= 0.2)	97.9	-8.8
	214 H	0.9	0.5	1.0	0.0	0.0	0.0	0.0	0.0	88.9% (>= 0.2)	97.9	-8.8
	214 K	0.9	0.5	1.0	0.0	0.0	0.0	0.0	0.0	88.7% (>= 0.2)	97.9	-8.8
	214 R	0.9	0.5	1.0	0.0	0.0	0.0	0.0	0.0	88.7% (>= 0.2)	97.9	-8.8
Epitope E	69 A	0.9	0.5	1.0	0.0	0.0	0.0	0.0	0.0	88.5% (>= 0.2)	97.9	-8.7
	69 F	0.9	0.5	1.0	0.0	0.0	0.0	0.0	0.0	88.5% (>= 0.2)	97.9	-8.7
	69 G	0.9	0.5	1.0	0.0	0.0	0.0	0.0	0.0	88.5% (>= 0.2)	97.9	-8.7
	69 I	0.9	0.5	1.0	0.0	0.0	0.0	0.0	0.0	89.3% (>= 0.2)	97.9	-8.7
	69 L	0.9	0.5	1.0	0.0	0.0	0.0	0.0	0.0	88.5% (>= 0.2)	97.9	-8.7
	69 M	0.9	0.5	1.0	0.0	0.0	0.0	0.0	0.0	88.5% (>= 0.2)	97.9	-8.7
	69 P	0.9	0.7	1.0	0.0	0.0	0.0	0.0	0.0	89.3% (>= 0.2)	97.9	-8.7
	69 V	0.9	0.5	1.0	0.0	0.0	0.0	0.0	0.0	89.3% (>= 0.2)	97.9	-8.7
	69 W	0.9	0.7	1.0	0.0	0.0	0.0	0.0	0.0	88.9% (>= 0.2)	97.9	-8.7
	69 C	0.9	0.5	1.0	0.0	0.0	0.0	0.0	0.0	88.9% (>= 0.2)	97.9	-8.7
	69 N	0.9	0.5	1.0	0.0	0.0	0.0	0.0	0.0	88.5% (>= 0.2)	97.9	-8.8
	69 Q	0.9	0.5	1.0	0.0	0.0	0.0	0.0	0.0	88.5% (>= 0.2)	97.9	-8.8
	69 S	0.9	0.5	1.0	0.0	0.0	0.0	0.0	0.0	88.9% (>= 0.2)	97.9	-8.8
	69 T	0.9	0.5	1.0	0.0	0.0	0.0	0.0	0.0	88.9% (>= 0.2)	97.9	-8.8
	69 Y	0.9	0.5	1.0	0.0	0.0	0.0	0.0	0.0	88.5% (>= 0.2)	97.9	-8.7
	69 D	0.9	0.5	1.0	0.0	0.0	0.0	0.0	0.0	88.9% (>= 0.2)	97.9	-8.8
	69 E	0.9	0.5	1.0	0.0	0.0	0.0	0.0	0.0	88.9% (>= 0.2)	97.9	-8.8
	69 H	0.9	0.5	1.0	0.0	0.0	0.0	0.0	0.0	88.9% (>= 0.2)	97.9	-8.8
	69 K	0.9	0.5	1.0	0.0	0.0	0.0	0.0	0.0	88.7% (>= 0.2)	97.9	-8.8
	69 R	0.9	0.5	1.0	0.0	0.0	0.0	0.0	0.0	88.5% (>= 0.2)	97.9	-8.7

Appendix D: Accession numbers.

Dataset 1: Accession numbers of global HA sequences (n= 596) used for Time-scaled phylogenies.

Selected from Bangladesh (n=55)

Accession number	Date of Collection
EPI_ISL_74079	2009-05-17
EPI_ISL_74078	2009-04-16
EPI_ISL_60775	2009-06-06
EPI_ISL_60774	2009-05-08
EPI_ISL_60773	2009-05-06
EPI_ISL_84089	2010-08-19
EPI_ISL_84088	2010-09-16
EPI_ISL_84087	2010-09-16
EPI_ISL_100423	2011-08-08
EPI_ISL_99071	2011-05-19
EPI_ISL_99070	2011-05-19
EPI_ISL_99069	2011-05-19
EPI_ISL_98636	2011-07-13
EPI_ISL_260564	2012-08-21
EPI_ISL_260563	2012-08-15
EPI_ISL_260562	2012-07-24
EPI_ISL_260561	2012-07-17
EPI_ISL_132113	2012-09-25
EPI_ISL_157089	2013-11-24

Accession number	Date of Collection
EPI_ISL_151804	2013-05-12
EPI_ISL_150284	2013-07-22
EPI_ISL_150281	2013-07-13
EPI_ISL_145040	2013-05-12
EPI_ISL_168105	2014-06-09
EPI_ISL_166789	2014-05-27
EPI_ISL_223995	2015-10-05
EPI_ISL_212959	2015-10-03
EPI_ISL_211677	2015-09-10
EPI_ISL_208596	2015-11-18
EPI_ISL_201271	2015-08-29
EPI_ISL_238626	2016-07-10
EPI_ISL_233472	2016-06-21
EPI_ISL_233452	2016-06-11
EPI_ISL_233443	2016-07-02
EPI_ISL_233440	2016-07-12
EPI_ISL_285881	2017-07-01
EPI_ISL_277159	2017-06-11
EPI_ISL_584002	2020-01-03

Accession number	Date of Collection
EPI_ISL_277154	2017-06-04
EPI_ISL_277149	2017-07-01
EPI_ISL_270162	2017-04-25
EPI_ISL_332954	2018-08-13
EPI_ISL_336711	2018-08-01
EPI_ISL_334154	2018-08-27
EPI_ISL_330939	2018-06-11
EPI_ISL_330926	2018-07-11
EPI_ISL_398391	2019-07-18
EPI_ISL_398362	2019-08-05
EPI_ISL_395398	2019-07-03
EPI_ISL_391157	2019-06-22
EPI_ISL_390067	2019-06-11
EPI_ISL_717682	2020-10-01
EPI_ISL_717681	2020-09-05
EPI_ISL_717673	2020-10-07
EPI_ISL_584003	2020-08-26

Selected from East Asia; EA (China/ Korea/ Japan), n= 60

Accession number	Date of Collection
EPI_ISL_70212	2009-11-25
EPI_ISL_32238	2009-03-03
EPI_ISL_32233	2009-03-12
EPI_ISL_74090	2009-05-23
EPI_ISL_32232	2009-01-06
EPI_ISL_148655	2010-09-08
EPI_ISL_83689	2010-09-06
EPI_ISL_83687	2010-08-04
EPI_ISL_83685	2010-07-07
EPI_ISL_83684	2010-05-12
EPI_ISL_278393	2011-12-01
EPI_ISL_101485	2011-11-16
EPI_ISL_101345	2011-11-18
EPI_ISL_94610	2011-02-14
EPI_ISL_121883	2011-12-24
EPI_ISL_328898	2012-08-06

Accession number	Date of Collection
EPI_ISL_148669	2012-03-05
EPI_ISL_137258	2012-12-25
EPI_ISL_134799	2012-12-04
EPI_ISL_132497	2012-09-10
EPI_ISL_156391	2013-01-29
EPI_ISL_148095	2013-06-06
EPI_ISL_153911	2013-11-25
EPI_ISL_153316	2013-09-25
EPI_ISL_143706	2013-04-11
EPI_ISL_176327	2014-12-12
EPI_ISL_173112	2014-03-29
EPI_ISL_176989	2014-01-24
EPI_ISL_164688	2014-03-24
EPI_ISL_158175	2014-01-29
EPI_ISL_190369	2015-01-19
EPI_ISL_207678	2015-12-06

Accession number	Date of Collection
EPI_ISL_178004	2015-01-08
EPI_ISL_213994	2015-07-07
EPI_ISL_190379	2015-04-02
EPI_ISL_242504	2016-11-13
EPI_ISL_225352	2016-03-03
EPI_ISL_225316	2016-04-13
EPI_ISL_223601	2016-01-06
EPI_ISL_242821	2016-12-04
EPI_ISL_328854	2017-06-19
EPI_ISL_302548	2017-05-16
EPI_ISL_267067	2017-04-15
EPI_ISL_262088	2017-03-22
EPI_ISL_258271	2017-03-07
EPI_ISL_313914	2018-01-25
EPI_ISL_314314	2018-02-01
EPI_ISL_334513	2018-12-11

Accession number	Date of Collection
EPI_ISL_334509	2018-11-20
EPI_ISL_334503	2018-11-06
EPI_ISL_367449	2019-04-24
EPI_ISL_367364	2019-05-22

Accession number	Date of Collection
EPI_ISL_367025	2019-04-29
EPI_ISL_405836	2019-01-17
EPI_ISL_710500	2019-11-24
EPI_ISL_480451	2020-01-05

Accession number	Date of Collection
EPI_ISL_684054	2020-02-14
EPI_ISL_528884	2020-01-08
EPI_ISL_485913	2020-01-08
EPI_ISL_485466	2020-02-17

Selected from European Union; EU (Germany/ Greece/ Spain/ Sweden), n= 60

Accession number	Date of Collection
EPI_ISL_61774	2009-01-15
EPI_ISL_70808	2009-01-14
EPI_ISL_70807	2009-01-02
EPI_ISL_70683	2009-01-22
EPI_ISL_70662	2009-02-11
EPI_ISL_86163	2010-12-22
EPI_ISL_86049	2010-12-17
EPI_ISL_85730	2010-10-22
EPI_ISL_82220	2010-10-01
EPI_ISL_81390	2010-04-29
EPI_ISL_100469	2011-11-27
EPI_ISL_100465	2011-11-19
EPI_ISL_97047	2011-09-05
EPI_ISL_107887	2011-12-02
EPI_ISL_103271	2011-09-05
EPI_ISL_219848	2012-01-28
EPI_ISL_102138	2012-01-20
EPI_ISL_132904	2012-10-30
EPI_ISL_107884	2012-01-12
EPI_ISL_219846	2012-01-02

Accession number	Date of Collection
EPI_ISL_141805	2013-02-13
EPI_ISL_152952	2013-12-04
EPI_ISL_138313	2013-02-05
EPI_ISL_141081	2013-02-05
EPI_ISL_153705	2013-12-07
EPI_ISL_169134	2014-11-03
EPI_ISL_167959	2014-10-19
EPI_ISL_167958	2014-10-25
EPI_ISL_170317	2014-12-24
EPI_ISL_155888	2014-01-13
EPI_ISL_215753	2015-12-22
EPI_ISL_177077	2015-03-05
EPI_ISL_181575	2015-02-02
EPI_ISL_190907	2015-02-09
EPI_ISL_213095	2015-10-30
EPI_ISL_253958	2016-12-28
EPI_ISL_245008	2016-12-17
EPI_ISL_242989	2016-12-16
EPI_ISL_244208	2016-11-16
EPI_ISL_248874	2016-12-17

Accession number	Date of Collection
EPI_ISL_292040	2017-12-13
EPI_ISL_285986	2017-11-18
EPI_ISL_273196	2017-01-25
EPI_ISL_262263	2017-01-16
EPI_ISL_274117	2017-01-21
EPI_ISL_310252	2018-03-14
EPI_ISL_303911	2018-01-24
EPI_ISL_315989	2018-03-07
EPI_ISL_314925	2018-04-02
EPI_ISL_304561	2018-01-08
EPI_ISL_355217	2019-01-24
EPI_ISL_347802	2019-02-26
EPI_ISL_343372	2019-01-29
EPI_ISL_345255	2019-01-17
EPI_ISL_391081	2019-08-18
EPI_ISL_410967	2020-01-21
EPI_ISL_434428	2020-02-14
EPI_ISL_405715	2020-01-14
EPI_ISL_529044	2020-02-24
EPI_ISL_485777	2020-02-04

Selected from Central Asia; CA (India/ Kazakhstan/ Uzbekistan), n= 56

Accession number	Date of Collection
EPI_ISL_165167	2009-08-13
EPI_ISL_165166	2009-08-14
EPI_ISL_165161	2009-09-13
EPI_ISL_165159	2009-08-09
EPI_ISL_69326	2009-08-18
EPI_ISL_165156	2010-08-10
EPI_ISL_165155	2010-08-10
EPI_ISL_165154	2010-09-02
EPI_ISL_89776	2010-10-25
EPI_ISL_84059	2010-09-06
EPI_ISL_235205	2011-09-12
EPI_ISL_99884	2011-02-24

Accession number	Date of Collection
EPI_ISL_105769	2011-11-14
EPI_ISL_96096	2011-06-20
EPI_ISL_96094	2011-06-29
EPI_ISL_161481	2012-12-07
EPI_ISL_161478	2012-10-26
EPI_ISL_107871	2012-01-01
EPI_ISL_134827	2012-02-02
EPI_ISL_125911	2012-01-30
EPI_ISL_161499	2013-02-20
EPI_ISL_154756	2013-12-23
EPI_ISL_154753	2013-12-18
EPI_ISL_138455	2013-01-17

Accession number	Date of Collection
EPI_ISL_156805	2013-01-27
EPI_ISL_175231	2014-12-29
EPI_ISL_165640	2014-03-04
EPI_ISL_179354	2014-11-25
EPI_ISL_164818	2014-06-05
EPI_ISL_164429	2014-01-11
EPI_ISL_206129	2015-11-03
EPI_ISL_191967	2015-02-23
EPI_ISL_189849	2015-01-21
EPI_ISL_175248	2015-01-05
EPI_ISL_175232	2015-01-02
EPI_ISL_262207	2016-11-21

Accession number	Date of Collection
EPI_ISL_262202	2016-11-21
EPI_ISL_248439	2016-12-12
EPI_ISL_226089	2016-02-22
EPI_ISL_234046	2016-02-09
EPI_ISL_281726	2017-02-14
EPI_ISL_281725	2017-02-14
EPI_ISL_270205	2017-01-28

Accession number	Date of Collection
EPI_ISL_296437	2017-09-08
EPI_ISL_296425	2017-09-15
EPI_ISL_306257	2018-01-31
EPI_ISL_305846	2018-01-10
EPI_ISL_303798	2018-02-08
EPI_ISL_303796	2018-01-16
EPI_ISL_331515	2018-03-29

Accession number	Date of Collection
EPI_ISL_412107	2019-11-30
EPI_ISL_409987	2019-12-22
EPI_ISL_367815	2019-03-05
EPI_ISL_367803	2019-02-13
EPI_ISL_404740	2019-08-16
EPI_ISL_534009	2020-02-05

Selected from North America; NA (Canada/ USA) 10 strain/year, n= 120

Accession number	Date of Collection
EPI_ISL_77961	2009-02-01
EPI_ISL_77769	2009-04-30
EPI_ISL_62497	2009-04-28
EPI_ISL_62495	2009-04-30
EPI_ISL_62487	2009-04-28
EPI_ISL_136579	2009-06-07
EPI_ISL_98965	2009-06-07
EPI_ISL_32308	2009-02-05
EPI_ISL_60740	2009-05-11
EPI_ISL_32297	2009-01-26
EPI_ISL_145077	2010-11-26
EPI_ISL_84033	2010-09-03
EPI_ISL_85591	2010-11-16
EPI_ISL_84037	2010-08-24
EPI_ISL_86092	2010-10-23
EPI_ISL_79663	2010-08-06
EPI_ISL_86107	2010-12-28
EPI_ISL_87935	2010-11-30
EPI_ISL_85587	2010-12-04
EPI_ISL_84068	2010-10-14
EPI_ISL_158625	2011-01-24
EPI_ISL_119389	2011-02-14
EPI_ISL_135251	2011-02-10
EPI_ISL_89792	2011-02-19
EPI_ISL_90862	2011-03-02
EPI_ISL_89840	2011-02-08
EPI_ISL_102456	2011-11-29
EPI_ISL_98641	2011-03-02
EPI_ISL_102990	2011-10-06
EPI_ISL_90823	2011-03-29
EPI_ISL_147967	2012-12-24
EPI_ISL_147961	2012-12-04
EPI_ISL_147945	2012-11-12

Accession number	Date of Collection
EPI_ISL_160398	2012-12-31
EPI_ISL_132807	2012-10-05
EPI_ISL_129427	2012-08-06
EPI_ISL_129410	2012-08-02
EPI_ISL_109749	2012-02-02
EPI_ISL_370275	2012-12-18
EPI_ISL_369592	2012-12-27
EPI_ISL_202132	2013-01-28
EPI_ISL_189636	2013-01-20
EPI_ISL_369602	2013-02-14
EPI_ISL_160328	2013-02-02
EPI_ISL_160280	2013-01-10
EPI_ISL_149674	2013-01-30
EPI_ISL_140990	2013-03-13
EPI_ISL_164406	2013-12-29
EPI_ISL_145125	2013-07-04
EPI_ISL_138721	2013-02-20
EPI_ISL_172607	2014-10-18
EPI_ISL_157767	2014-01-29
EPI_ISL_170022	2014-11-24
EPI_ISL_159519	2014-03-06
EPI_ISL_170698	2014-12-05
EPI_ISL_251071	2014-12-23
EPI_ISL_250954	2014-12-01
EPI_ISL_251039	2014-12-10
EPI_ISL_250345	2014-11-20
EPI_ISL_250259	2014-10-14
EPI_ISL_176709	2015-01-17
EPI_ISL_175177	2015-02-03
EPI_ISL_174181	2015-01-11
EPI_ISL_200750	2015-09-13
EPI_ISL_179010	2015-03-22
EPI_ISL_178981	2015-03-03

Accession number	Date of Collection
EPI_ISL_251247	2015-01-06
EPI_ISL_203451	2015-07-01
EPI_ISL_206243	2015-12-13
EPI_ISL_211709	2015-12-04
EPI_ISL_234053	2016-08-22
EPI_ISL_220299	2016-02-24
EPI_ISL_224026	2016-02-17
EPI_ISL_219270	2016-03-04
EPI_ISL_219198	2016-02-12
EPI_ISL_289306	2016-12-01
EPI_ISL_240189	2016-11-25
EPI_ISL_239211	2016-09-26
EPI_ISL_239224	2016-10-30
EPI_ISL_239705	2016-11-18
EPI_ISL_261805	2017-03-09
EPI_ISL_249084	2017-01-13
EPI_ISL_287522	2017-03-04
EPI_ISL_287241	2017-03-16
EPI_ISL_286710	2017-02-19
EPI_ISL_286656	2017-02-07
EPI_ISL_286599	2017-02-12
EPI_ISL_286574	2017-02-07
EPI_ISL_358238	2017-12-19
EPI_ISL_294274	2017-09-25
EPI_ISL_316759	2018-01-25
EPI_ISL_300772	2018-02-02
EPI_ISL_327318	2018-01-08
EPI_ISL_336759	2018-10-26
EPI_ISL_320720	2018-07-16
EPI_ISL_361518	2018-03-01
EPI_ISL_376832	2018-02-14
EPI_ISL_376645	2018-01-31
EPI_ISL_376580	2018-02-06

Accession number	Date of Collection
EPI_ISL_373474	2018-02-15
EPI_ISL_344623	2019-01-03
EPI_ISL_359027	2019-01-18
EPI_ISL_359015	2019-02-01
EPI_ISL_359435	2019-03-13
EPI_ISL_360100	2019-03-06
EPI_ISL_369008	2019-04-03

Accession number	Date of Collection
EPI_ISL_380670	2019-03-27
EPI_ISL_380515	2019-03-24
EPI_ISL_502040	2019-05-05
EPI_ISL_409391	2019-12-26
EPI_ISL_427949	2020-02-01
EPI_ISL_449731	2020-03-20
EPI_ISL_424732	2020-02-04

Accession number	Date of Collection
EPI_ISL_421092	2020-02-13
EPI_ISL_413744	2020-02-05
EPI_ISL_435328	2020-03-15
EPI_ISL_416806	2020-02-19
EPI_ISL_424739	2020-03-07
EPI_ISL_415774	2020-01-19
EPI_ISL_416768	2020-02-09

Selected from Oceania; OS (Australia/ New Zealand), n= 60

Accession number	Date of Collection
EPI_ISL_60760	2009-01-01
EPI_ISL_172958	2009-02-07
EPI_ISL_76618	2009-07-05
EPI_ISL_76619	2009-11-04
EPI_ISL_60149	2009-05-07
EPI_ISL_79465	2010-07-12
EPI_ISL_83869	2010-10-15
EPI_ISL_86123	2010-12-07
EPI_ISL_83861	2010-10-01
EPI_ISL_83226	2010-09-09
EPI_ISL_94867	2011-04-12
EPI_ISL_118579	2011-12-04
EPI_ISL_99734	2011-08-08
EPI_ISL_11860	2011-12-16
EPI_ISL_95093	2011-04-07
EPI_ISL_288360	2012-08-13
EPI_ISL_128704	2012-07-02
EPI_ISL_192789	2012-07-27
EPI_ISL_192785	2012-07-23
EPI_ISL_128650	2012-07-05

Accession number	Date of Collection
EPI_ISL_288242	2013-10-24
EPI_ISL_172979	2013-02-01
EPI_ISL_145684	2013-06-17
EPI_ISL_161901	2013-11-21
EPI_ISL_145321	2013-04-30
EPI_ISL_288215	2014-02-09
EPI_ISL_288186	2014-08-11
EPI_ISL_166746	2014-06-14
EPI_ISL_165591	2014-03-16
EPI_ISL_168916	2014-07-09
EPI_ISL_287937	2015-08-17
EPI_ISL_287891	2015-08-17
EPI_ISL_287834	2015-10-08
EPI_ISL_200719	2015-04-22
EPI_ISL_197525	2015-06-04
EPI_ISL_289123	2016-05-31
EPI_ISL_288832	2016-10-29
EPI_ISL_227630	2016-02-22
EPI_ISL_246675	2016-12-31
EPI_ISL_244051	2016-09-04

Accession number	Date of Collection
EPI_ISL_275253	2017-07-06
EPI_ISL_291364	2017-09-07
EPI_ISL_277541	2017-07-24
EPI_ISL_396648	2017-08-16
EPI_ISL_396534	2017-08-14
EPI_ISL_396652	2018-02-26
EPI_ISL_329732	2018-07-26
EPI_ISL_320378	2018-03-23
EPI_ISL_330022	2018-03-17
EPI_ISL_396473	2018-10-05
EPI_ISL_356625	2019-03-29
EPI_ISL_404956	2019-05-16
EPI_ISL_365267	2019-06-05
EPI_ISL_389274	2019-07-04
EPI_ISL_356644	2019-03-14
EPI_ISL_517769	2020-01-29
EPI_ISL_528758	2020-03-16
EPI_ISL_419608	2020-01-20
EPI_ISL_410359	2020-01-07
EPI_ISL_545575	2020-01-15

Selected from South-East Asia; SEA (Indonesia/ Malaysia/ Philippines/ Singapore/ Thailand/ Vietnam), n= 60

Accession number	Date of Collection
EPI_ISL_143044	2009-05-22
EPI_ISL_143015	2009-06-26
EPI_ISL_208774	2009-10-29
EPI_ISL_145179	2009-06-16
EPI_ISL_70197	2009-05-05
EPI_ISL_143053	2010-08-14
EPI_ISL_143052	2010-05-22
EPI_ISL_145207	2010-04-08
EPI_ISL_145203	2010-06-17

Accession number	Date of Collection
EPI_ISL_89836	2010-02-08
EPI_ISL_235271	2011-11-29
EPI_ISL_205407	2011-08-24
EPI_ISL_123004	2011-12-05
EPI_ISL_123001	2011-11-10
EPI_ISL_122962	2011-12-09
EPI_ISL_399276	2012-12-01
EPI_ISL_399256	2012-07-30
EPI_ISL_235211	2012-02-14

Accession number	Date of Collection
EPI_ISL_208793	2012-07-30
EPI_ISL_208780	2012-12-07
EPI_ISL_205364	2013-01-28
EPI_ISL_399250	2013-12-09
EPI_ISL_205439	2013-12-11
EPI_ISL_157088	2013-09-12
EPI_ISL_165922	2013-12-06
EPI_ISL_205457	2014-11-18
EPI_ISL_205450	2014-08-05

Accession number	Date of Collection
EPI_ISL_192831	2014-05-30
EPI_ISL_172604	2014-12-15
EPI_ISL_167845	2014-01-13
EPI_ISL_252447	2015-07-29
EPI_ISL_213025	2015-10-16
EPI_ISL_212372	2015-10-20
EPI_ISL_215710	2015-03-31
EPI_ISL_215701	2015-02-16
EPI_ISL_240800	2016-12-05
EPI_ISL_240772	2016-10-04
EPI_ISL_239326	2016-08-11

Accession number	Date of Collection
EPI_ISL_239131	2016-07-21
EPI_ISL_249244	2016-11-01
EPI_ISL_283062	2017-06-06
EPI_ISL_283009	2017-09-25
EPI_ISL_255583	2017-02-06
EPI_ISL_255569	2017-02-03
EPI_ISL_291290	2017-09-18
EPI_ISL_344190	2018-12-07
EPI_ISL_322075	2018-01-03
EPI_ISL_349924	2018-05-27
EPI_ISL_341163	2018-05-27

Accession number	Date of Collection
EPI_ISL_333863	2018-02-01
EPI_ISL_400299	2019-10-07
EPI_ISL_400298	2019-08-19
EPI_ISL_391670	2019-04-03
EPI_ISL_391596	2019-01-10
EPI_ISL_409058	2019-10-16
EPI_ISL_481340	2020-01-02
EPI_ISL_481336	2020-01-21
EPI_ISL_514710	2020-01-02
EPI_ISL_510012	2020-02-07
EPI_ISL_644650	2020-01-13

Selected from Southern Hemisphere; SH (Namibia/ Madagascar/ South Africa/ Argentina/ Brazil/ Chili/ Peru), n= 60

Accession number	Date of Collection
EPI_ISL_34979	2009-01-01
EPI_ISL_60769	2009-05-05
EPI_ISL_60763	2009-06-26
EPI_ISL_65119	2009-07-09
EPI_ISL_60916	2009-06-08
EPI_ISL_152833	2010-11-15
EPI_ISL_152840	2010-11-20
EPI_ISL_152282	2010-10-13
EPI_ISL_152146	2010-10-30
EPI_ISL_87951	2010-11-30
EPI_ISL_152593	2011-10-07
EPI_ISL_152260	2011-11-15
EPI_ISL_263182	2011-07-14
EPI_ISL_263144	2011-04-06
EPI_ISL_235307	2011-10-07
EPI_ISL_152589	2012-04-09
EPI_ISL_152584	2012-02-08
EPI_ISL_152497	2012-03-07
EPI_ISL_125910	2012-04-11
EPI_ISL_128065	2012-06-04

Accession number	Date of Collection
EPI_ISL_145642	2013-06-03
EPI_ISL_148739	2013-08-02
EPI_ISL_145113	2013-06-06
EPI_ISL_145527	2013-02-25
EPI_ISL_145487	2013-03-11
EPI_ISL_263170	2014-07-11
EPI_ISL_166794	2014-01-20
EPI_ISL_168112	2014-02-19
EPI_ISL_166719	2014-06-03
EPI_ISL_166716	2014-07-01
EPI_ISL_263242	2015-05-29
EPI_ISL_201225	2015-05-18
EPI_ISL_205201	2015-07-22
EPI_ISL_205194	2015-06-15
EPI_ISL_205192	2015-06-15
EPI_ISL_244823	2016-09-11
EPI_ISL_230308	2016-04-27
EPI_ISL_274149	2016-12-23
EPI_ISL_232562	2016-06-14
EPI_ISL_231815	2016-06-02

Accession number	Date of Collection
EPI_ISL_274175	2017-02-21
EPI_ISL_275807	2017-01-22
EPI_ISL_278118	2017-07-13
EPI_ISL_281742	2017-07-20
EPI_ISL_279223	2017-06-06
EPI_ISL_355840	2018-08-28
EPI_ISL_346980	2018-06-27
EPI_ISL_322156	2018-05-22
EPI_ISL_322963	2018-04-09
EPI_ISL_329855	2018-06-28
EPI_ISL_367546	2019-01-30
EPI_ISL_393597	2019-05-03
EPI_ISL_393591	2019-04-23
EPI_ISL_584010	2019-12-16
EPI_ISL_388768	2019-06-10
EPI_ISL_516175	2020-02-25
EPI_ISL_516144	2020-01-30
EPI_ISL_516189	2020-02-20
EPI_ISL_516098	2020-03-05
EPI_ISL_584007	2020-03-02

Selected from United Kingdom; UK, n= 57

Accession number	Date of Collection
EPI_ISL_60046	2009-07-01
EPI_ISL_60047	2009-07-01
EPI_ISL_60048	2009-07-01
EPI_ISL_90600	2010-12-13
EPI_ISL_88034	2010-12-06

Accession number	Date of Collection
EPI_ISL_85727	2010-11-26
EPI_ISL_85726	2010-09-27
EPI_ISL_99876	2011-11-07
EPI_ISL_99875	2011-10-10
EPI_ISL_99874	2011-10-12

Accession number	Date of Collection
EPI_ISL_99872	2011-09-11
EPI_ISL_105765	2011-11-07
EPI_ISL_132005	2012-04-18
EPI_ISL_121909	2012-03-19
EPI_ISL_121928	2012-04-03

Accession number	Date of Collection
EPI_ISL_121927	2012-04-03
EPI_ISL_117470	2012-03-30
EPI_ISL_154749	2013-12-18
EPI_ISL_153157	2013-10-16
EPI_ISL_141675	2013-02-15
EPI_ISL_141672	2013-01-30
EPI_ISL_141669	2013-01-09
EPI_ISL_192200	2014-12-10
EPI_ISL_192195	2014-11-20
EPI_ISL_188792	2014-12-15
EPI_ISL_188768	2014-11-28
EPI_ISL_286310	2014-12-12
EPI_ISL_192262	2015-04-01
EPI_ISL_186613	2015-02-11

Accession number	Date of Collection
EPI_ISL_287017	2015-01-21
EPI_ISL_286942	2015-01-04
EPI_ISL_285510	2015-01-14
EPI_ISL_270463	2016-12-30
EPI_ISL_269791	2016-12-06
EPI_ISL_248521	2016-12-23
EPI_ISL_274388	2016-09-06
EPI_ISL_273942	2016-12-28
EPI_ISL_292269	2017-12-16
EPI_ISL_286917	2017-09-30
EPI_ISL_270528	2017-02-01
EPI_ISL_270419	2017-02-18
EPI_ISL_274883	2017-01-02
EPI_ISL_314162	2018-02-09

Accession number	Date of Collection
EPI_ISL_312054	2018-03-01
EPI_ISL_303486	2018-01-25
EPI_ISL_303415	2018-02-06
EPI_ISL_327399	2018-04-06
EPI_ISL_405114	2019-11-04
EPI_ISL_399842	2019-10-24
EPI_ISL_400525	2019-12-06
EPI_ISL_404293	2019-12-03
EPI_ISL_487025	2019-12-24
EPI_ISL_480515	2020-01-09
EPI_ISL_480494	2020-01-06
EPI_ISL_471039	2020-01-04
EPI_ISL_463769	2020-02-21
EPI_ISL_414060	2020-01-06

Dataset 2: Selected potential vaccine candidate (p-vac) from each season.

Season (WHO recommended Vaccine)	P-Vac Strain Name	Accession number	Country	Date of Collection
2015-16 (A/Switzerland/9715293/2013)	A/Bangladesh/3400	EPI_ISL_195894	Bangladesh	2015-05-25
	A/India/0609	EPI_ISL_212992	India	2015-02-21
	A/Nakhonratchaisma/35	EPI_ISL_197500	Thailand	2015-05-16
	A/Singapore/EN283	EPI_ISL_215830	Singapore	2015-04-30
	A/South Australia/1001	EPI_ISL_288038	Australia	2015-05-15
	A/Victoria/7	EPI_ISL_288040		
2016-17 (Hong_Kong/4801/2014)	A/Bangladesh/455	EPI_ISL_233460	Bangladesh	2016-06-02
	A/India/1460	EPI_ISL_234046	India	2016-02-09
	A/Bangkok/175	EPI_ISL_232109	Thailand	2016-04-26
	A/Singapore/INFKK-16-0542	EPI_ISL_225830	Singapore	2016-05-16
	A/Victoria/504	EPI_ISL_233842	Australia	2016-05-15
2017-18 (Hong_Kong/4801/2014)	A/Bangladesh/8007	EPI_ISL_277271	Bangladesh	2017-05-16
	Bangladesh/5012	EPI_ISL_277272		
	A/Trivandrum/MCVRAG1073	EPI_ISL_329338	India	2017-05-16
	A/Thailand/CU-B21873	EPI_ISL_290143	Thailand	2017-05-15
	A/Singapore/TT0555	EPI_ISL_269771	Singapore	2017-05-12
	A/Sydney/119	EPI_ISL_271318	Australia	2017-05-15
	A/Brisbane/61	EPI_ISL_271281		
	A/South Australia/110	EPI_ISL_271265		
	A/Brisbane/74	EPI_ISL_396283		
2018-19 (Singapore/INFIMH-16-0019/2016)	A/Bangladesh/455	EPI_ISL_233460	Bangladesh	2016-06-02
	A/India/1460	EPI_ISL_234046	India	2016-02-09
	A/Bangkok/175	EPI_ISL_232109	Thailand	2016-04-26
	A/Singapore/INFKK-16-0542	EPI_ISL_225830	Singapore	2016-05-16
	A/Victoria/504	EPI_ISL_233842	Australia	2016-05-15
2019-20 (A/Kansas/14/2017)	A/Bangladesh/3025	EPI_ISL_368171	Bangladesh	2019-05-15
	A/India/0534	EPI_ISL_391120	India	2019-04-10
	A/Lopburi/235	EPI_ISL_390099	Thailand	2019-05-15
	A/Singapore/OHC0015	EPI_ISL_392640	Singapore	2019-05-14
	A/Sydney/1027	EPI_ISL_391462	Australia	2019-05-15
	A/Perth/1020	EPI_ISL_405892		
	A/Victoria/1008	EPI_ISL_391453		
	A/Sydney/811	EPI_ISL_405908		
	A/Victoria/30	EPI_ISL_391400		

Dataset 3: Sequences from 2015-16 to 2019-20 seasons

Selected from South Korea (n=144)

**Season 2015-16**	EPI_ISL_378755	EPI_ISL_376771	EPI_ISL_369948	EPI_ISL_502014	EPI_ISL_427990
EPI_ISL_253937	EPI_ISL_378754	EPI_ISL_320990	EPI_ISL_370172	EPI_ISL_368982	EPI_ISL_427991
EPI_ISL_258599	EPI_ISL_258604	EPI_ISL_331252	EPI_ISL_502013	EPI_ISL_369037	EPI_ISL_427993
EPI_ISL_212984	EPI_ISL_258605	EPI_ISL_310077	EPI_ISL_502021	EPI_ISL_369038	EPI_ISL_414881
EPI_ISL_212983	EPI_ISL_378757	EPI_ISL_310078	EPI_ISL_502022	EPI_ISL_502025	EPI_ISL_405870
EPI_ISL_302500	**Season 2017-18**	EPI_ISL_310079	EPI_ISL_502032	EPI_ISL_370022	EPI_ISL_414854
EPI_ISL_253938	EPI_ISL_311679	EPI_ISL_331253	EPI_ISL_502031	EPI_ISL_502028	EPI_ISL_485512
EPI_ISL_258597	EPI_ISL_292819	EPI_ISL_321856	EPI_ISL_368880	EPI_ISL_502029	EPI_ISL_485513
EPI_ISL_258598	EPI_ISL_307858	EPI_ISL_373480	EPI_ISL_405869	EPI_ISL_502030	EPI_ISL_485514
EPI_ISL_302498	EPI_ISL_331251	EPI_ISL_376769	EPI_ISL_366581	EPI_ISL_376524	EPI_ISL_485516
**Season 2016-17**	EPI_ISL_376766	EPI_ISL_320994	EPI_ISL_366587	EPI_ISL_376540	EPI_ISL_405462
EPI_ISL_378752	EPI_ISL_292818	EPI_ISL_307859	EPI_ISL_366582	EPI_ISL_376541	EPI_ISL_405872
EPI_ISL_302501	EPI_ISL_376770	EPI_ISL_376767	EPI_ISL_501979	EPI_ISL_376542	EPI_ISL_414877
EPI_ISL_258602	EPI_ISL_505577	**Season 2018-19**	EPI_ISL_369108	EPI_ISL_376530	EPI_ISL_405873
EPI_ISL_258603	EPI_ISL_376765	EPI_ISL_502026	EPI_ISL_502023	**Season 2019-20**	EPI_ISL_485515
EPI_ISL_292820	EPI_ISL_376582	EPI_ISL_346643	EPI_ISL_366584	EPI_ISL_414155	EPI_ISL_414879
EPI_ISL_302499	EPI_ISL_376764	EPI_ISL_366520	EPI_ISL_366585	EPI_ISL_405871	EPI_ISL_414882
EPI_ISL_378753	EPI_ISL_376840	EPI_ISL_370023	EPI_ISL_366586	EPI_ISL_405452	EPI_ISL_414883
EPI_ISL_378756	EPI_ISL_321846	EPI_ISL_341448	EPI_ISL_366583	EPI_ISL_405459	EPI_ISL_414884
EPI_ISL_292816	EPI_ISL_505581	EPI_ISL_341449	EPI_ISL_366588	EPI_ISL_435229	EPI_ISL_414885
EPI_ISL_292817	EPI_ISL_505580	EPI_ISL_370020	EPI_ISL_366589	EPI_ISL_405453	EPI_ISL_414886
EPI_ISL_258601	EPI_ISL_376583	EPI_ISL_370021	EPI_ISL_366590	EPI_ISL_412543	EPI_ISL_414887
EPI_ISL_258606	EPI_ISL_307856	EPI_ISL_502024	EPI_ISL_366591	EPI_ISL_414142	EPI_ISL_414888
EPI_ISL_258600	EPI_ISL_376763	EPI_ISL_366522	EPI_ISL_372593	EPI_ISL_414845	EPI_ISL_414889
	EPI_ISL_376768	EPI_ISL_366523	EPI_ISL_501980	EPI_ISL_414851	EPI_ISL_414890

Selected from Japan (n=1409)

**Season 2015-16**	EPI_ISL_213435	EPI_ISL_205812	EPI_ISL_235035	EPI_ISL_218450	EPI_ISL_235043
EPI_ISL_225427	EPI_ISL_218458	EPI_ISL_208266	EPI_ISL_229802	EPI_ISL_218457	EPI_ISL_222745
EPI_ISL_205816	EPI_ISL_222747	EPI_ISL_214007	EPI_ISL_229798	EPI_ISL_224693	EPI_ISL_229786
EPI_ISL_236851	EPI_ISL_229783	EPI_ISL_218961	EPI_ISL_225421	EPI_ISL_229788	EPI_ISL_229789
EPI_ISL_224694	EPI_ISL_215162	EPI_ISL_229784	EPI_ISL_224701	EPI_ISL_229794	EPI_ISL_224688
EPI_ISL_218444	EPI_ISL_229799	EPI_ISL_229785	EPI_ISL_224698	EPI_ISL_213432	EPI_ISL_215161
EPI_ISL_225429	EPI_ISL_236841	EPI_ISL_232763	EPI_ISL_224696	EPI_ISL_218454	EPI_ISL_213329
EPI_ISL_224699	EPI_ISL_236843	EPI_ISL_229800	EPI_ISL_224695	EPI_ISL_224219	EPI_ISL_222746
EPI_ISL_231964	EPI_ISL_225433	EPI_ISL_229787	EPI_ISL_224228	EPI_ISL_229790	EPI_ISL_224687
EPI_ISL_231965	EPI_ISL_229807	EPI_ISL_224700	EPI_ISL_222750	EPI_ISL_229806	EPI_ISL_229793
EPI_ISL_210372	EPI_ISL_224689	EPI_ISL_218461	EPI_ISL_222749	EPI_ISL_231963	EPI_ISL_236842
EPI_ISL_218452	EPI_ISL_215157	EPI_ISL_215153	EPI_ISL_218453	EPI_ISL_237572	EPI_ISL_208271
EPI_ISL_218455	EPI_ISL_231967	EPI_ISL_210373	EPI_ISL_229803	EPI_ISL_203065	EPI_ISL_202069
EPI_ISL_224686	EPI_ISL_210371	EPI_ISL_208267	EPI_ISL_218451	EPI_ISL_208238	EPI_ISL_213434
EPI_ISL_231962	EPI_ISL_215163	EPI_ISL_208268	EPI_ISL_218448	EPI_ISL_203066	EPI_ISL_208273
EPI_ISL_222744	EPI_ISL_224697	EPI_ISL_205813	EPI_ISL_215158	EPI_ISL_203062	EPI_ISL_203059
EPI_ISL_236849	EPI_ISL_215151	EPI_ISL_205814	EPI_ISL_215154	EPI_ISL_203063	EPI_ISL_210374
EPI_ISL_224230	EPI_ISL_222748	EPI_ISL_203064	EPI_ISL_210370	EPI_ISL_203067	EPI_ISL_218456
EPI_ISL_225432	EPI_ISL_235042	EPI_ISL_211890	EPI_ISL_205810	EPI_ISL_208272	EPI_ISL_213433
EPI_ISL_224227	EPI_ISL_229801	EPI_ISL_229804	EPI_ISL_205811	EPI_ISL_202068	EPI_ISL_213431
EPI_ISL_218459	EPI_ISL_224220	EPI_ISL_215155	EPI_ISL_215160	EPI_ISL_208243	EPI_ISL_215156
EPI_ISL_222742	EPI_ISL_225417	EPI_ISL_236844	EPI_ISL_218449	EPI_ISL_237571	EPI_ISL_202066

EPI_ISL_201174	EPI_ISL_276995	EPI_ISL_273494	EPI_ISL_254701	EPI_ISL_252777	EPI_ISL_277007
EPI_ISL_201175	EPI_ISL_239595	EPI_ISL_257708	EPI_ISL_282950	EPI_ISL_267782	EPI_ISL_247735
EPI_ISL_208241	EPI_ISL_242819	EPI_ISL_253081	EPI_ISL_245988	EPI_ISL_257924	EPI_ISL_254710
EPI_ISL_229791	EPI_ISL_263355	EPI_ISL_252779	EPI_ISL_239587	EPI_ISL_288997	EPI_ISL_263347
EPI_ISL_218460	EPI_ISL_247746	EPI_ISL_262076	EPI_ISL_239593	EPI_ISL_282948	EPI_ISL_291149
EPI_ISL_229796	EPI_ISL_254714	EPI_ISL_267063	EPI_ISL_239599	EPI_ISL_296094	EPI_ISL_282947
EPI_ISL_205815	EPI_ISL_253079	EPI_ISL_246757	EPI_ISL_239615	EPI_ISL_287740	EPI_ISL_277006
EPI_ISL_215165	EPI_ISL_262990	EPI_ISL_257928	EPI_ISL_239621	EPI_ISL_264335	EPI_ISL_262090
EPI_ISL_224222	EPI_ISL_257917	EPI_ISL_258258	EPI_ISL_242823	EPI_ISL_248913	EPI_ISL_264338
EPI_ISL_225420	EPI_ISL_254706	EPI_ISL_246759	EPI_ISL_246741	EPI_ISL_279061	EPI_ISL_257927
EPI_ISL_218446	EPI_ISL_242839	EPI_ISL_248932	EPI_ISL_267775	EPI_ISL_279060	EPI_ISL_253182
EPI_ISL_201173	EPI_ISL_252772	EPI_ISL_240549	EPI_ISL_274668	EPI_ISL_258262	EPI_ISL_247736
EPI_ISL_218447	EPI_ISL_237972	EPI_ISL_240725	EPI_ISL_274669	EPI_ISL_273490	EPI_ISL_240729
EPI_ISL_225431	EPI_ISL_277002	EPI_ISL_255894	EPI_ISL_267783	EPI_ISL_248917	EPI_ISL_240731
EPI_ISL_229792	EPI_ISL_237971	EPI_ISL_254699	EPI_ISL_292541	EPI_ISL_237967	EPI_ISL_242835
EPI_ISL_215164	EPI_ISL_255898	EPI_ISL_240543	EPI_ISL_273492	EPI_ISL_237973	EPI_ISL_242840
EPI_ISL_213436	EPI_ISL_255899	EPI_ISL_240544	EPI_ISL_273493	EPI_ISL_237974	EPI_ISL_242841
EPI_ISL_229797	EPI_ISL_237566	EPI_ISL_258252	EPI_ISL_274664	EPI_ISL_238900	EPI_ISL_247738
EPI_ISL_213430	EPI_ISL_239606	EPI_ISL_258254	EPI_ISL_267057	EPI_ISL_238901	EPI_ISL_248920
EPI_ISL_203060	EPI_ISL_273506	EPI_ISL_282956	EPI_ISL_277429	EPI_ISL_238902	EPI_ISL_252765
EPI_ISL_203061	EPI_ISL_258255	EPI_ISL_267781	EPI_ISL_275598	EPI_ISL_239589	EPI_ISL_252767
EPI_ISL_224702	EPI_ISL_254702	EPI_ISL_259766	EPI_ISL_273495	EPI_ISL_239617	EPI_ISL_252768
EPI_ISL_215150	EPI_ISL_250877	EPI_ISL_257926	EPI_ISL_282951	EPI_ISL_237975	EPI_ISL_252769
EPI_ISL_202310	EPI_ISL_240542	EPI_ISL_248908	EPI_ISL_277433	EPI_ISL_238903	EPI_ISL_252773
EPI_ISL_208269	EPI_ISL_253068	EPI_ISL_267058	EPI_ISL_284431	EPI_ISL_247741	EPI_ISL_252774
EPI_ISL_208270	EPI_ISL_240559	EPI_ISL_259771	EPI_ISL_273496	EPI_ISL_239594	EPI_ISL_253075
EPI_ISL_224692	EPI_ISL_247743	EPI_ISL_248927	EPI_ISL_267785	EPI_ISL_242818	EPI_ISL_253082
EPI_ISL_229805	EPI_ISL_237417	EPI_ISL_262088	EPI_ISL_267066	EPI_ISL_257918	EPI_ISL_254712
EPI_ISL_224691	EPI_ISL_237420	EPI_ISL_313911	EPI_ISL_267062	EPI_ISL_248930	EPI_ISL_254715
EPI_ISL_222743	EPI_ISL_237421	EPI_ISL_258268	EPI_ISL_263348	EPI_ISL_255895	EPI_ISL_255896
EPI_ISL_208245	EPI_ISL_238898	EPI_ISL_267784	EPI_ISL_262080	EPI_ISL_258264	EPI_ISL_257707
EPI_ISL_208242	EPI_ISL_238899	EPI_ISL_331214	EPI_ISL_274663	EPI_ISL_262069	EPI_ISL_262987
EPI_ISL_215152	EPI_ISL_240541	EPI_ISL_267065	EPI_ISL_257921	EPI_ISL_267776	EPI_ISL_276993
EPI_ISL_215159	EPI_ISL_240546	EPI_ISL_254703	EPI_ISL_264320	EPI_ISL_258270	EPI_ISL_242836
EPI_ISL_218445	EPI_ISL_240547	EPI_ISL_242837	EPI_ISL_277430	EPI_ISL_248903	EPI_ISL_257714
EPI_ISL_224690	EPI_ISL_240548	EPI_ISL_252770	EPI_ISL_276998	EPI_ISL_262082	EPI_ISL_277434
EPI_ISL_208244	EPI_ISL_242838	EPI_ISL_240557	EPI_ISL_274667	EPI_ISL_262078	EPI_ISL_292542
**Season 2016-17**	EPI_ISL_252771	EPI_ISL_250878	EPI_ISL_267778	EPI_ISL_246754	EPI_ISL_279062
EPI_ISL_240561	EPI_ISL_263350	EPI_ISL_248928	EPI_ISL_267064	EPI_ISL_252776	EPI_ISL_305340
EPI_ISL_240563	EPI_ISL_262071	EPI_ISL_248924	EPI_ISL_258261	EPI_ISL_277427	EPI_ISL_289002
EPI_ISL_240555	EPI_ISL_258266	EPI_ISL_264337	EPI_ISL_273498	EPI_ISL_257704	EPI_ISL_277439
EPI_ISL_240027	EPI_ISL_246740	EPI_ISL_259773	EPI_ISL_273499	EPI_ISL_248925	EPI_ISL_277437
EPI_ISL_240545	EPI_ISL_257919	EPI_ISL_264332	EPI_ISL_273497	EPI_ISL_248918	EPI_ISL_273503
EPI_ISL_240552	EPI_ISL_259764	EPI_ISL_274670	EPI_ISL_253080	EPI_ISL_246742	EPI_ISL_273502
EPI_ISL_239585	EPI_ISL_240551	EPI_ISL_277001	EPI_ISL_257929	EPI_ISL_245992	EPI_ISL_277431
EPI_ISL_239590	EPI_ISL_248905	EPI_ISL_287738	EPI_ISL_263353	EPI_ISL_237568	EPI_ISL_277435
EPI_ISL_239613	EPI_ISL_239609	EPI_ISL_267067	EPI_ISL_273501	EPI_ISL_240550	EPI_ISL_282946
EPI_ISL_239618	EPI_ISL_313910	EPI_ISL_263356	EPI_ISL_276999	EPI_ISL_246753	EPI_ISL_282952
EPI_ISL_237565	EPI_ISL_258271	EPI_ISL_263352	EPI_ISL_264330	EPI_ISL_247742	EPI_ISL_267056
EPI_ISL_237567	EPI_ISL_276992	EPI_ISL_258263	EPI_ISL_274685	EPI_ISL_252775	EPI_ISL_277441
EPI_ISL_239586	EPI_ISL_247739	EPI_ISL_254698	EPI_ISL_264321	EPI_ISL_245991	EPI_ISL_282955
EPI_ISL_239603	EPI_ISL_253074	EPI_ISL_252778	EPI_ISL_277438	EPI_ISL_262086	EPI_ISL_277432
EPI_ISL_239614	EPI_ISL_262986	EPI_ISL_248907	EPI_ISL_259772	EPI_ISL_240562	EPI_ISL_257930
EPI_ISL_239604	EPI_ISL_281381	EPI_ISL_248902	EPI_ISL_246755	EPI_ISL_248922	EPI_ISL_292540

EPI_ISL_277004	EPI_ISL_242820	EPI_ISL_273849	EPI_ISL_257712	EPI_ISL_320559	EPI_ISL_316501
EPI_ISL_273505	EPI_ISL_242821	EPI_ISL_258259	EPI_ISL_253069	EPI_ISL_319635	EPI_ISL_316113
EPI_ISL_267772	EPI_ISL_252766	EPI_ISL_253067	EPI_ISL_248931	EPI_ISL_311970	EPI_ISL_316101
EPI_ISL_263354	EPI_ISL_239592	EPI_ISL_257705	EPI_ISL_237418	EPI_ISL_311964	EPI_ISL_315226
EPI_ISL_254711	EPI_ISL_239620	EPI_ISL_258265	EPI_ISL_240721	EPI_ISL_293304	EPI_ISL_315179
EPI_ISL_254717	EPI_ISL_264329	EPI_ISL_248926	EPI_ISL_248904	EPI_ISL_306948	EPI_ISL_314319
EPI_ISL_258256	EPI_ISL_245989	EPI_ISL_273504	EPI_ISL_282757	EPI_ISL_306951	EPI_ISL_314314
EPI_ISL_277005	EPI_ISL_237970	EPI_ISL_282957	EPI_ISL_264333	EPI_ISL_319631	EPI_ISL_314306
EPI_ISL_262077	EPI_ISL_259765	EPI_ISL_246761	EPI_ISL_257709	EPI_ISL_308927	EPI_ISL_314293
EPI_ISL_282069	EPI_ISL_263357	EPI_ISL_263351	EPI_ISL_239605	EPI_ISL_316100	EPI_ISL_310080
EPI_ISL_276991	EPI_ISL_263358	EPI_ISL_257923	EPI_ISL_240722	EPI_ISL_366211	EPI_ISL_311968
EPI_ISL_262084	EPI_ISL_255900	EPI_ISL_274665	EPI_ISL_255903	EPI_ISL_313914	EPI_ISL_315230
EPI_ISL_240553	EPI_ISL_250875	EPI_ISL_287737	EPI_ISL_255897	EPI_ISL_306945	EPI_ISL_315233
EPI_ISL_249075	EPI_ISL_239607	EPI_ISL_248933	EPI_ISL_237419	EPI_ISL_320555	EPI_ISL_376743
EPI_ISL_259129	EPI_ISL_247740	EPI_ISL_254716	EPI_ISL_264334	EPI_ISL_322120	EPI_ISL_307398
EPI_ISL_240554	EPI_ISL_248929	EPI_ISL_248923	EPI_ISL_262075	EPI_ISL_320989	EPI_ISL_306953
EPI_ISL_263359	EPI_ISL_257706	EPI_ISL_254700	EPI_ISL_262072	EPI_ISL_320561	EPI_ISL_291145
EPI_ISL_254704	EPI_ISL_254705	EPI_ISL_248916	EPI_ISL_258267	EPI_ISL_320558	EPI_ISL_310074
EPI_ISL_267779	EPI_ISL_262079	EPI_ISL_258260	EPI_ISL_250879	EPI_ISL_315234	EPI_ISL_314298
EPI_ISL_257920	EPI_ISL_253073	EPI_ISL_264326	EPI_ISL_240726	EPI_ISL_315231	EPI_ISL_310075
EPI_ISL_277428	EPI_ISL_262985	EPI_ISL_264328	**Season 2017-18**	EPI_ISL_314310	EPI_ISL_319628
EPI_ISL_263361	EPI_ISL_253083	EPI_ISL_246756	EPI_ISL_310071	EPI_ISL_308931	EPI_ISL_330081
EPI_ISL_263362	EPI_ISL_250876	EPI_ISL_281375	EPI_ISL_320986	EPI_ISL_306952	EPI_ISL_376744
EPI_ISL_263360	EPI_ISL_237968	EPI_ISL_257922	EPI_ISL_311975	EPI_ISL_373452	EPI_ISL_313913
EPI_ISL_262089	EPI_ISL_240556	EPI_ISL_267059	EPI_ISL_316505	EPI_ISL_376742	EPI_ISL_291162
EPI_ISL_259768	EPI_ISL_237969	EPI_ISL_255902	EPI_ISL_320156	EPI_ISL_376567	EPI_ISL_315216
EPI_ISL_259769	EPI_ISL_277440	EPI_ISL_239608	EPI_ISL_308924	EPI_ISL_330082	EPI_ISL_305562
EPI_ISL_259770	EPI_ISL_282958	EPI_ISL_258257	EPI_ISL_331843	EPI_ISL_315228	EPI_ISL_315180
EPI_ISL_258253	EPI_ISL_247737	EPI_ISL_248906	EPI_ISL_320569	EPI_ISL_314322	EPI_ISL_288995
EPI_ISL_246760	EPI_ISL_273507	EPI_ISL_239601	EPI_ISL_319638	EPI_ISL_322118	EPI_ISL_289504
EPI_ISL_248914	EPI_ISL_267773	EPI_ISL_239602	EPI_ISL_319637	EPI_ISL_330085	EPI_ISL_289505
EPI_ISL_248921	EPI_ISL_267060	EPI_ISL_264327	EPI_ISL_315229	EPI_ISL_322117	EPI_ISL_293302
EPI_ISL_253076	EPI_ISL_274666	EPI_ISL_282962	EPI_ISL_314317	EPI_ISL_320155	EPI_ISL_311965
EPI_ISL_257716	EPI_ISL_273500	EPI_ISL_259767	EPI_ISL_315219	EPI_ISL_319634	EPI_ISL_316099
EPI_ISL_267780	EPI_ISL_277003	EPI_ISL_254697	EPI_ISL_310076	EPI_ISL_319633	EPI_ISL_288994
EPI_ISL_254713	EPI_ISL_276996	EPI_ISL_262070	EPI_ISL_315223	EPI_ISL_319630	EPI_ISL_288990
EPI_ISL_240558	EPI_ISL_276994	EPI_ISL_237569	EPI_ISL_330083	EPI_ISL_316502	EPI_ISL_291172
EPI_ISL_240560	EPI_ISL_268527	EPI_ISL_253078	EPI_ISL_319632	EPI_ISL_316109	EPI_ISL_293301
EPI_ISL_239600	EPI_ISL_267774	EPI_ISL_262989	EPI_ISL_316098	EPI_ISL_316103	EPI_ISL_314296
EPI_ISL_246758	EPI_ISL_276997	EPI_ISL_240728	EPI_ISL_311971	EPI_ISL_315225	EPI_ISL_314297
EPI_ISL_257715	EPI_ISL_267777	EPI_ISL_237564	EPI_ISL_316105	EPI_ISL_313909	EPI_ISL_304124
EPI_ISL_258269	EPI_ISL_267068	EPI_ISL_262074	EPI_ISL_320566	EPI_ISL_307389	EPI_ISL_302796
EPI_ISL_263349	EPI_ISL_262083	EPI_ISL_240727	EPI_ISL_320560	EPI_ISL_293299	EPI_ISL_288991
EPI_ISL_253077	EPI_ISL_247744	EPI_ISL_248919	EPI_ISL_314308	EPI_ISL_319626	EPI_ISL_291163
EPI_ISL_262988	EPI_ISL_262073	EPI_ISL_240723	EPI_ISL_322113	EPI_ISL_315235	EPI_ISL_292813
EPI_ISL_239598	EPI_ISL_262081	EPI_ISL_240724	EPI_ISL_320564	EPI_ISL_319629	EPI_ISL_311963
EPI_ISL_242822	EPI_ISL_262085	EPI_ISL_253164	EPI_ISL_314307	EPI_ISL_376570	EPI_ISL_289509
EPI_ISL_239588	EPI_ISL_262087	EPI_ISL_255901	EPI_ISL_307392	EPI_ISL_376745	EPI_ISL_305553
EPI_ISL_239591	EPI_ISL_263363	EPI_ISL_239611	EPI_ISL_314321	EPI_ISL_331841	EPI_ISL_320562
EPI_ISL_239596	EPI_ISL_263364	EPI_ISL_242834	EPI_ISL_322115	EPI_ISL_331619	EPI_ISL_307396
EPI_ISL_239597	EPI_ISL_277000	EPI_ISL_240028	EPI_ISL_311974	EPI_ISL_330581	EPI_ISL_307399
EPI_ISL_239616	EPI_ISL_257925	EPI_ISL_254696	EPI_ISL_320988	EPI_ISL_322114	EPI_ISL_327656
EPI_ISL_239619	EPI_ISL_277436	EPI_ISL_257713	EPI_ISL_308926	EPI_ISL_319636	EPI_ISL_307394
EPI_ISL_240730	EPI_ISL_245990	EPI_ISL_239610	EPI_ISL_330584	EPI_ISL_319627	EPI_ISL_297229

EPI_ISL_304126	EPI_ISL_320154	EPI_ISL_297226	EPI_ISL_345215	EPI_ISL_376980	EPI_ISL_356445
EPI_ISL_315224	EPI_ISL_291147	EPI_ISL_311962	EPI_ISL_364629	EPI_ISL_344511	EPI_ISL_356868
EPI_ISL_306949	EPI_ISL_297222	EPI_ISL_292809	EPI_ISL_346656	EPI_ISL_344509	EPI_ISL_356869
EPI_ISL_294306	EPI_ISL_294323	EPI_ISL_311969	EPI_ISL_502011	EPI_ISL_376975	EPI_ISL_361865
EPI_ISL_315227	EPI_ISL_304127	EPI_ISL_376741	EPI_ISL_405843	EPI_ISL_341454	EPI_ISL_364625
EPI_ISL_306943	EPI_ISL_315232	EPI_ISL_315222	EPI_ISL_395206	EPI_ISL_341445	EPI_ISL_364626
EPI_ISL_297225	EPI_ISL_315215	EPI_ISL_291161	EPI_ISL_391436	EPI_ISL_340691	EPI_ISL_365569
EPI_ISL_376568	EPI_ISL_314315	EPI_ISL_307390	EPI_ISL_389100	EPI_ISL_340692	EPI_ISL_365574
EPI_ISL_311961	EPI_ISL_306503	EPI_ISL_307391	EPI_ISL_381715	EPI_ISL_337457	EPI_ISL_366073
EPI_ISL_311972	EPI_ISL_306954	EPI_ISL_322116	EPI_ISL_376985	EPI_ISL_337458	EPI_ISL_366074
EPI_ISL_291141	EPI_ISL_315217	EPI_ISL_327653	EPI_ISL_376986	EPI_ISL_340689	EPI_ISL_366088
EPI_ISL_312784	EPI_ISL_306946	EPI_ISL_327652	EPI_ISL_376971	EPI_ISL_340690	EPI_ISL_366089
EPI_ISL_311966	EPI_ISL_292806	EPI_ISL_297227	EPI_ISL_376970	EPI_ISL_336975	EPI_ISL_366095
EPI_ISL_320568	EPI_ISL_302790	EPI_ISL_308928	EPI_ISL_376969	EPI_ISL_405846	EPI_ISL_366097
EPI_ISL_314313	EPI_ISL_308930	EPI_ISL_320987	EPI_ISL_376972	EPI_ISL_376979	EPI_ISL_366111
EPI_ISL_316108	EPI_ISL_311967	EPI_ISL_316107	EPI_ISL_371938	EPI_ISL_405836	EPI_ISL_366538
EPI_ISL_306687	EPI_ISL_312777	EPI_ISL_330582	EPI_ISL_371936	EPI_ISL_348324	EPI_ISL_366699
EPI_ISL_306685	EPI_ISL_315177	EPI_ISL_308933	EPI_ISL_370112	EPI_ISL_376976	EPI_ISL_366748
EPI_ISL_312771	EPI_ISL_314312	EPI_ISL_327657	EPI_ISL_369912	EPI_ISL_1716902	EPI_ISL_370352
EPI_ISL_299134	EPI_ISL_307397	EPI_ISL_330084	EPI_ISL_366747	EPI_ISL_405849	EPI_ISL_370593
EPI_ISL_306686	EPI_ISL_313912	EPI_ISL_320567	EPI_ISL_371926	EPI_ISL_405848	EPI_ISL_371924
EPI_ISL_312772	EPI_ISL_306947	EPI_ISL_320565	EPI_ISL_371933	EPI_ISL_405838	EPI_ISL_371925
EPI_ISL_312785	EPI_ISL_304125	EPI_ISL_302789	EPI_ISL_371937	EPI_ISL_405837	EPI_ISL_371929
EPI_ISL_294304	EPI_ISL_306682	EPI_ISL_327663	EPI_ISL_366539	EPI_ISL_333607	EPI_ISL_371934
EPI_ISL_306684	EPI_ISL_314309	EPI_ISL_320563	EPI_ISL_366478	EPI_ISL_333609	EPI_ISL_371935
EPI_ISL_312770	EPI_ISL_304128	EPI_ISL_314320	EPI_ISL_366114	EPI_ISL_332908	EPI_ISL_376968
EPI_ISL_282944	EPI_ISL_320556	**Season 2018-19**	EPI_ISL_366113	EPI_ISL_332911	EPI_ISL_376973
EPI_ISL_282945	EPI_ISL_289510	EPI_ISL_356427	EPI_ISL_366099	EPI_ISL_336970	EPI_ISL_376977
EPI_ISL_331842	EPI_ISL_306504	EPI_ISL_346663	EPI_ISL_376966	EPI_ISL_339720	EPI_ISL_391099
EPI_ISL_315221	EPI_ISL_308929	EPI_ISL_346660	EPI_ISL_366091	EPI_ISL_339839	EPI_ISL_395227
EPI_ISL_291138	EPI_ISL_294324	EPI_ISL_346653	EPI_ISL_366082	EPI_ISL_340694	EPI_ISL_395228
EPI_ISL_289507	EPI_ISL_315220	EPI_ISL_345227	EPI_ISL_366078	EPI_ISL_340695	EPI_ISL_395229
EPI_ISL_315178	EPI_ISL_294302	EPI_ISL_356746	EPI_ISL_366077	EPI_ISL_340696	EPI_ISL_395230
EPI_ISL_305563	EPI_ISL_299135	EPI_ISL_345216	EPI_ISL_365581	EPI_ISL_340697	EPI_ISL_395308
EPI_ISL_308923	EPI_ISL_320984	EPI_ISL_354788	EPI_ISL_365571	EPI_ISL_340698	EPI_ISL_398728
EPI_ISL_308925	EPI_ISL_310072	EPI_ISL_357493	EPI_ISL_363749	EPI_ISL_340699	EPI_ISL_400632
EPI_ISL_316102	EPI_ISL_291160	EPI_ISL_364630	EPI_ISL_357499	EPI_ISL_341446	EPI_ISL_1716901
EPI_ISL_314318	EPI_ISL_288992	EPI_ISL_366079	EPI_ISL_357494	EPI_ISL_341447	EPI_ISL_346664
EPI_ISL_311973	EPI_ISL_320985	EPI_ISL_344522	EPI_ISL_356878	EPI_ISL_341453	EPI_ISL_412121
EPI_ISL_314305	EPI_ISL_288996	EPI_ISL_405845	EPI_ISL_356751	EPI_ISL_344519	EPI_ISL_361860
EPI_ISL_315218	EPI_ISL_289508	EPI_ISL_395205	EPI_ISL_356430	EPI_ISL_344521	EPI_ISL_361861
EPI_ISL_306944	EPI_ISL_288989	EPI_ISL_348326	EPI_ISL_347536	EPI_ISL_345211	EPI_ISL_336977
EPI_ISL_314316	EPI_ISL_304122	EPI_ISL_356428	EPI_ISL_346650	EPI_ISL_345212	EPI_ISL_357495
EPI_ISL_297218	EPI_ISL_306950	EPI_ISL_356429	EPI_ISL_346649	EPI_ISL_345223	EPI_ISL_333921
EPI_ISL_293298	EPI_ISL_291164	EPI_ISL_361863	EPI_ISL_345225	EPI_ISL_345226	EPI_ISL_333922
EPI_ISL_291137	EPI_ISL_297219	EPI_ISL_398303	EPI_ISL_345222	EPI_ISL_346065	EPI_ISL_333924
EPI_ISL_289506	EPI_ISL_316106	EPI_ISL_391100	EPI_ISL_356741	EPI_ISL_346227	EPI_ISL_333927
EPI_ISL_289502	EPI_ISL_314311	EPI_ISL_381707	EPI_ISL_376974	EPI_ISL_346654	EPI_ISL_336959
EPI_ISL_297223	EPI_ISL_308932	EPI_ISL_365575	EPI_ISL_345218	EPI_ISL_346655	EPI_ISL_1005694
EPI_ISL_288993	EPI_ISL_304121	EPI_ISL_357497	EPI_ISL_345219	EPI_ISL_346657	EPI_ISL_356425
EPI_ISL_320554	EPI_ISL_304123	EPI_ISL_405842	EPI_ISL_366080	EPI_ISL_348322	EPI_ISL_376978
EPI_ISL_320153	EPI_ISL_320557	EPI_ISL_364628	EPI_ISL_366081	EPI_ISL_356432	EPI_ISL_376982
EPI_ISL_289503	EPI_ISL_316104	EPI_ISL_356433	EPI_ISL_366084	EPI_ISL_356436	EPI_ISL_1716903
EPI_ISL_297220	EPI_ISL_310073	EPI_ISL_356446	EPI_ISL_344520	EPI_ISL_356442	EPI_ISL_366070

EPI_ISL_366096	EPI_ISL_369763	EPI_ISL_370355	EPI_ISL_405847	EPI_ISL_572186	EPI_ISL_405852
EPI_ISL_336965	EPI_ISL_369764	EPI_ISL_370108	EPI_ISL_366108	EPI_ISL_1064126	EPI_ISL_480453
EPI_ISL_337451	EPI_ISL_370113	EPI_ISL_370111	EPI_ISL_395208	EPI_ISL_577615	EPI_ISL_414387
EPI_ISL_337452	EPI_ISL_341441	EPI_ISL_363750	EPI_ISL_356426	EPI_ISL_1064124	EPI_ISL_405855
EPI_ISL_337453	EPI_ISL_1005690	EPI_ISL_354789	EPI_ISL_366083	EPI_ISL_498215	EPI_ISL_405857
EPI_ISL_370109	EPI_ISL_337455	EPI_ISL_363718	EPI_ISL_366085	EPI_ISL_415179	EPI_ISL_405853
EPI_ISL_333920	EPI_ISL_337456	EPI_ISL_348325	EPI_ISL_366086	EPI_ISL_403088	EPI_ISL_684048
EPI_ISL_333931	EPI_ISL_337459	EPI_ISL_405830	EPI_ISL_369705	EPI_ISL_395532	EPI_ISL_684049
EPI_ISL_333928	EPI_ISL_337460	EPI_ISL_376984	EPI_ISL_340687	EPI_ISL_411135	EPI_ISL_684050
EPI_ISL_336961	EPI_ISL_340021	EPI_ISL_370595	EPI_ISL_336960	EPI_ISL_413234	EPI_ISL_400568
EPI_ISL_372696	EPI_ISL_1716900	EPI_ISL_370364	EPI_ISL_340686	EPI_ISL_405850	EPI_ISL_428430
EPI_ISL_395209	EPI_ISL_364627	EPI_ISL_366876	EPI_ISL_381721	EPI_ISL_395536	EPI_ISL_517667
EPI_ISL_411127	EPI_ISL_336974	EPI_ISL_366110	EPI_ISL_392485	EPI_ISL_419328	EPI_ISL_594224
EPI_ISL_413232	EPI_ISL_1005691	EPI_ISL_366094	EPI_ISL_398305	EPI_ISL_428433	EPI_ISL_398727
EPI_ISL_398729	EPI_ISL_370354	EPI_ISL_366093	EPI_ISL_363725	EPI_ISL_414386	EPI_ISL_400565
EPI_ISL_505770	EPI_ISL_1716904	EPI_ISL_366090	EPI_ISL_366746	EPI_ISL_480452	EPI_ISL_415178
EPI_ISL_395204	EPI_ISL_356440	EPI_ISL_366071	EPI_ISL_363744	EPI_ISL_414390	EPI_ISL_498218
EPI_ISL_333926	EPI_ISL_365585	EPI_ISL_357504	EPI_ISL_341451	EPI_ISL_454740	EPI_ISL_405851
EPI_ISL_395207	EPI_ISL_364651	EPI_ISL_356871	EPI_ISL_341452	EPI_ISL_417061	EPI_ISL_535490
EPI_ISL_340018	EPI_ISL_394199	EPI_ISL_356748	EPI_ISL_405829	EPI_ISL_498206	EPI_ISL_414388
EPI_ISL_346644	EPI_ISL_371922	EPI_ISL_356742	EPI_ISL_1005692	EPI_ISL_405877	EPI_ISL_392480
EPI_ISL_336962	EPI_ISL_363479	EPI_ISL_356447	EPI_ISL_356444	EPI_ISL_414391	EPI_ISL_392482
EPI_ISL_336964	EPI_ISL_344510	EPI_ISL_356434	EPI_ISL_405835	EPI_ISL_414392	EPI_ISL_583905
EPI_ISL_333923	EPI_ISL_345224	EPI_ISL_356431	EPI_ISL_405833	EPI_ISL_498205	EPI_ISL_428428
EPI_ISL_376967	EPI_ISL_333608	EPI_ISL_352951	EPI_ISL_366749	EPI_ISL_408571	EPI_ISL_414397
EPI_ISL_336973	EPI_ISL_332909	EPI_ISL_350485	EPI_ISL_371930	EPI_ISL_428427	EPI_ISL_408546
EPI_ISL_341442	EPI_ISL_332910	EPI_ISL_350486	EPI_ISL_1005693	EPI_ISL_428435	EPI_ISL_414396
EPI_ISL_332411	EPI_ISL_361864	EPI_ISL_366072	EPI_ISL_394188	EPI_ISL_498209	EPI_ISL_584120
EPI_ISL_376983	EPI_ISL_371923	EPI_ISL_347535	EPI_ISL_366098	EPI_ISL_415176	EPI_ISL_405880
EPI_ISL_336963	EPI_ISL_368916	EPI_ISL_344513	EPI_ISL_365582	EPI_ISL_710501	EPI_ISL_414382
EPI_ISL_346658	EPI_ISL_366479	EPI_ISL_344512	EPI_ISL_356870	EPI_ISL_415183	EPI_ISL_517684
EPI_ISL_363478	EPI_ISL_364641	EPI_ISL_356747	EPI_ISL_348323	EPI_ISL_577614	EPI_ISL_417063
EPI_ISL_333919	EPI_ISL_346647	EPI_ISL_341444	EPI_ISL_346645	EPI_ISL_577616	EPI_ISL_469258
EPI_ISL_376981	EPI_ISL_346648	EPI_ISL_364654	EPI_ISL_344523	EPI_ISL_710502	EPI_ISL_428434
EPI_ISL_336969	EPI_ISL_333917	EPI_ISL_365572	EPI_ISL_357502	EPI_ISL_577608	EPI_ISL_454747
EPI_ISL_370351	EPI_ISL_365570	EPI_ISL_366075	EPI_ISL_370594	EPI_ISL_498217	EPI_ISL_517683
EPI_ISL_336971	EPI_ISL_333930	EPI_ISL_366112	EPI_ISL_370353	EPI_ISL_684054	EPI_ISL_584121
EPI_ISL_336968	EPI_ISL_333918	EPI_ISL_371927	EPI_ISL_356752	EPI_ISL_684053	EPI_ISL_405878
EPI_ISL_331837	EPI_ISL_341439	EPI_ISL_356872	EPI_ISL_356753	EPI_ISL_454745	EPI_ISL_415182
EPI_ISL_339838	EPI_ISL_376987	EPI_ISL_336976	EPI_ISL_356875	EPI_ISL_498216	EPI_ISL_414393
EPI_ISL_341440	EPI_ISL_333929	EPI_ISL_341443	EPI_ISL_356876	EPI_ISL_684052	EPI_ISL_480451
EPI_ISL_336966	EPI_ISL_336972	EPI_ISL_346659	EPI_ISL_356877	EPI_ISL_400553	EPI_ISL_710500
EPI_ISL_346662	EPI_ISL_505769	EPI_ISL_356874	EPI_ISL_405828	EPI_ISL_411130	EPI_ISL_400582
EPI_ISL_336967	EPI_ISL_356873	EPI_ISL_357500	EPI_ISL_371939	EPI_ISL_413233	EPI_ISL_684051
EPI_ISL_363715	EPI_ISL_370110	EPI_ISL_363714	EPI_ISL_371932	EPI_ISL_415177	EPI_ISL_405879
EPI_ISL_357498	EPI_ISL_370356	EPI_ISL_365573	EPI_ISL_366100	EPI_ISL_428437	EPI_ISL_480454
EPI_ISL_332409	EPI_ISL_357505	EPI_ISL_366076	EPI_ISL_366092	EPI_ISL_395196	EPI_ISL_400577
EPI_ISL_333925	EPI_ISL_371928	EPI_ISL_371931	EPI_ISL_365576	EPI_ISL_419327	EPI_ISL_400583
EPI_ISL_348327	EPI_ISL_370362	EPI_ISL_391422	EPI_ISL_363716	EPI_ISL_419329	EPI_ISL_428429
EPI_ISL_346661	EPI_ISL_392484	EPI_ISL_391435	EPI_ISL_357501	EPI_ISL_684055	EPI_ISL_400618
EPI_ISL_378923	EPI_ISL_381720	EPI_ISL_395226	**Season 2019-20**	EPI_ISL_428426	
EPI_ISL_369762	EPI_ISL_376965	EPI_ISL_405844	EPI_ISL_480456	EPI_ISL_415180	

Selected from UK (n=391)

Season 2015-16

EPI_ISL_233165
EPI_ISL_237666
EPI_ISL_237667
EPI_ISL_237598
EPI_ISL_237599
EPI_ISL_241329
EPI_ISL_241278
EPI_ISL_237600
EPI_ISL_241210
EPI_ISL_241243
EPI_ISL_241446
EPI_ISL_203379
EPI_ISL_241294

Season 2016-17

EPI_ISL_270544
EPI_ISL_286844
EPI_ISL_302464
EPI_ISL_270470
EPI_ISL_270522
EPI_ISL_270784
EPI_ISL_270528
EPI_ISL_248537
EPI_ISL_270550
EPI_ISL_270471
EPI_ISL_270531
EPI_ISL_270491
EPI_ISL_270568
EPI_ISL_276317
EPI_ISL_270420
EPI_ISL_270475
EPI_ISL_270448
EPI_ISL_270428
EPI_ISL_248497
EPI_ISL_274635
EPI_ISL_270514
EPI_ISL_270466
EPI_ISL_248572
EPI_ISL_270765
EPI_ISL_270521
EPI_ISL_270525
EPI_ISL_270559
EPI_ISL_270562
EPI_ISL_270565
EPI_ISL_270814
EPI_ISL_248483
EPI_ISL_248487
EPI_ISL_248515
EPI_ISL_270451
EPI_ISL_270464
EPI_ISL_270485
EPI_ISL_270504
EPI_ISL_270533
EPI_ISL_270443
EPI_ISL_270548
EPI_ISL_270396
EPI_ISL_270419
EPI_ISL_286817
EPI_ISL_286830
EPI_ISL_270407
EPI_ISL_248489
EPI_ISL_270392
EPI_ISL_270413
EPI_ISL_290995
EPI_ISL_286908
EPI_ISL_269817
EPI_ISL_302187
EPI_ISL_269897
EPI_ISL_269819
EPI_ISL_286787
EPI_ISL_286790
EPI_ISL_269825
EPI_ISL_269827
EPI_ISL_269865
EPI_ISL_269902
EPI_ISL_269846
EPI_ISL_269877
EPI_ISL_269834
EPI_ISL_274890
EPI_ISL_269791
EPI_ISL_248548
EPI_ISL_269805
EPI_ISL_269845
EPI_ISL_270782
EPI_ISL_239768
EPI_ISL_269802
EPI_ISL_274392
EPI_ISL_270770
EPI_ISL_269900
EPI_ISL_269855
EPI_ISL_269820
EPI_ISL_269821
EPI_ISL_269889
EPI_ISL_274658
EPI_ISL_270787
EPI_ISL_269792
EPI_ISL_248508
EPI_ISL_269824
EPI_ISL_269892
EPI_ISL_286788
EPI_ISL_269826
EPI_ISL_248506
EPI_ISL_270759
EPI_ISL_248553

Season 2017-18

EPI_ISL_304944
EPI_ISL_303429
EPI_ISL_304784
EPI_ISL_291096
EPI_ISL_312048
EPI_ISL_304850
EPI_ISL_303310
EPI_ISL_313954
EPI_ISL_304829
EPI_ISL_312132
EPI_ISL_303361
EPI_ISL_304891
EPI_ISL_301429
EPI_ISL_303321
EPI_ISL_294238
EPI_ISL_298087
EPI_ISL_301437
EPI_ISL_304901
EPI_ISL_298096
EPI_ISL_301398
EPI_ISL_292244
EPI_ISL_309442
EPI_ISL_303391
EPI_ISL_292291
EPI_ISL_301538
EPI_ISL_301453
EPI_ISL_304918
EPI_ISL_312057
EPI_ISL_304939
EPI_ISL_304883
EPI_ISL_304846
EPI_ISL_303415
EPI_ISL_301497
EPI_ISL_301403
EPI_ISL_293625
EPI_ISL_313830
EPI_ISL_313957
EPI_ISL_292301
EPI_ISL_304951
EPI_ISL_327409
EPI_ISL_312059
EPI_ISL_309470
EPI_ISL_303380
EPI_ISL_298093
EPI_ISL_292307
EPI_ISL_292264
EPI_ISL_298102
EPI_ISL_291009
EPI_ISL_291095
EPI_ISL_293680
EPI_ISL_303362
EPI_ISL_309451
EPI_ISL_327404
EPI_ISL_303412
EPI_ISL_303515
EPI_ISL_312094
EPI_ISL_312051
EPI_ISL_312033
EPI_ISL_307639
EPI_ISL_304937
EPI_ISL_304899
EPI_ISL_304895
EPI_ISL_303430
EPI_ISL_303375
EPI_ISL_303353
EPI_ISL_301539
EPI_ISL_301480
EPI_ISL_298083
EPI_ISL_301477
EPI_ISL_301529
EPI_ISL_303366
EPI_ISL_304832
EPI_ISL_304915
EPI_ISL_307644
EPI_ISL_309428
EPI_ISL_312065
EPI_ISL_301467
EPI_ISL_309461
EPI_ISL_286855
EPI_ISL_301451
EPI_ISL_291018
EPI_ISL_309468
EPI_ISL_301456
EPI_ISL_298012
EPI_ISL_304782
EPI_ISL_291015
EPI_ISL_327401
EPI_ISL_291002
EPI_ISL_301490
EPI_ISL_292300
EPI_ISL_291034
EPI_ISL_301542
EPI_ISL_301525
EPI_ISL_301537
EPI_ISL_304761
EPI_ISL_301461
EPI_ISL_292274
EPI_ISL_301541

Season 2018-19

EPI_ISL_366945
EPI_ISL_389199
EPI_ISL_356573
EPI_ISL_364714
EPI_ISL_514053
EPI_ISL_514048
EPI_ISL_514050
EPI_ISL_514010
EPI_ISL_514042
EPI_ISL_399804
EPI_ISL_514011
EPI_ISL_369067
EPI_ISL_389203
EPI_ISL_364713
EPI_ISL_514035
EPI_ISL_389207
EPI_ISL_343203
EPI_ISL_347140
EPI_ISL_347143
EPI_ISL_351410
EPI_ISL_334408
EPI_ISL_336080
EPI_ISL_338759
EPI_ISL_514056
EPI_ISL_513997
EPI_ISL_514014
EPI_ISL_514005
EPI_ISL_356580
EPI_ISL_357338
EPI_ISL_387437
EPI_ISL_399783
EPI_ISL_356708
EPI_ISL_514017
EPI_ISL_514025
EPI_ISL_356575
EPI_ISL_399787
EPI_ISL_338758
EPI_ISL_369014
EPI_ISL_356460
EPI_ISL_336081
EPI_ISL_336082
EPI_ISL_350624
EPI_ISL_366466
EPI_ISL_369692
EPI_ISL_370504
EPI_ISL_350731
EPI_ISL_343206
EPI_ISL_366731
EPI_ISL_335689
EPI_ISL_364744
EPI_ISL_356536
EPI_ISL_513974

Season 2019-20

EPI_ISL_470987
EPI_ISL_480491
EPI_ISL_428546
EPI_ISL_480457
EPI_ISL_459781
EPI_ISL_428599
EPI_ISL_428598
EPI_ISL_416208
EPI_ISL_459728
EPI_ISL_480464
EPI_ISL_470930
EPI_ISL_459750
EPI_ISL_428614
EPI_ISL_408292
EPI_ISL_408064
EPI_ISL_408012
EPI_ISL_405163
EPI_ISL_405222
EPI_ISL_405135
EPI_ISL_405132
EPI_ISL_405129
EPI_ISL_405095
EPI_ISL_408109
EPI_ISL_400614
EPI_ISL_405128
EPI_ISL_405218
EPI_ISL_405257
EPI_ISL_408033
EPI_ISL_408055
EPI_ISL_408100
EPI_ISL_408316
EPI_ISL_428567
EPI_ISL_459804
EPI_ISL_480541

EPI_ISL_459779	EPI_ISL_487014	EPI_ISL_459787	EPI_ISL_405235	EPI_ISL_428578	EPI_ISL_398664
EPI_ISL_480469	EPI_ISL_470943	EPI_ISL_459824	EPI_ISL_405190	EPI_ISL_428596	EPI_ISL_401559
EPI_ISL_428657	EPI_ISL_459828	EPI_ISL_480496	EPI_ISL_405184	EPI_ISL_459855	EPI_ISL_401555
EPI_ISL_459823	EPI_ISL_459813	EPI_ISL_400529	EPI_ISL_399833	EPI_ISL_401704	EPI_ISL_405236
EPI_ISL_459837	EPI_ISL_428636	EPI_ISL_398666	EPI_ISL_399835	EPI_ISL_470913	EPI_ISL_428573
EPI_ISL_459786	EPI_ISL_428580	EPI_ISL_405141	EPI_ISL_408067	EPI_ISL_480475	EPI_ISL_408110
EPI_ISL_428617	EPI_ISL_408376	EPI_ISL_408043	EPI_ISL_428576	EPI_ISL_480504	EPI_ISL_399962
EPI_ISL_416213	EPI_ISL_408359	EPI_ISL_408338	EPI_ISL_400543	EPI_ISL_459825	EPI_ISL_407600
EPI_ISL_428562	EPI_ISL_408038	EPI_ISL_459831	EPI_ISL_405082	EPI_ISL_479606	EPI_ISL_480511
EPI_ISL_408341	EPI_ISL_407601	EPI_ISL_480468	EPI_ISL_405112	EPI_ISL_459796	EPI_ISL_470915
EPI_ISL_405200	EPI_ISL_405106	EPI_ISL_459802	EPI_ISL_405188	EPI_ISL_459792	EPI_ISL_480487
EPI_ISL_405138	EPI_ISL_401702	EPI_ISL_459797	EPI_ISL_408058	EPI_ISL_400524	EPI_ISL_487041
EPI_ISL_408099	EPI_ISL_401688	EPI_ISL_459751	EPI_ISL_408075	EPI_ISL_405137	EPI_ISL_487073
EPI_ISL_416205	EPI_ISL_400590	EPI_ISL_459748	EPI_ISL_408116	EPI_ISL_413640	EPI_ISL_471009
EPI_ISL_428626	EPI_ISL_405115	EPI_ISL_459735	EPI_ISL_408356	EPI_ISL_480550	EPI_ISL_418450
EPI_ISL_428633	EPI_ISL_459791	EPI_ISL_459734	EPI_ISL_428502	EPI_ISL_408014	
EPI_ISL_471043	EPI_ISL_416211	EPI_ISL_408300	EPI_ISL_428503	EPI_ISL_399840	
EPI_ISL_487016	EPI_ISL_459758	EPI_ISL_408296	EPI_ISL_428522	EPI_ISL_405205	

Selected from USA (n=655)

**Season 2015-16**	EPI_ISL_201281	EPI_ISL_253785	EPI_ISL_259261	EPI_ISL_259804	EPI_ISL_289334
EPI_ISL_234479	EPI_ISL_212964	EPI_ISL_225113	EPI_ISL_253602	EPI_ISL_264457	EPI_ISL_287542
EPI_ISL_202436	EPI_ISL_202429	EPI_ISL_224039	EPI_ISL_286430	EPI_ISL_286470	EPI_ISL_287195
EPI_ISL_237353	EPI_ISL_202825	EPI_ISL_223177	EPI_ISL_247956	EPI_ISL_277874	EPI_ISL_268269
EPI_ISL_237317	EPI_ISL_205658	EPI_ISL_222058	EPI_ISL_259211	EPI_ISL_282843	EPI_ISL_239648
EPI_ISL_237362	EPI_ISL_207026	EPI_ISL_224015	EPI_ISL_286338	EPI_ISL_287547	EPI_ISL_272898
EPI_ISL_240113	EPI_ISL_211903	EPI_ISL_219221	EPI_ISL_286398	EPI_ISL_260394	EPI_ISL_272929
EPI_ISL_230337	EPI_ISL_202937	EPI_ISL_219260	EPI_ISL_286653	EPI_ISL_287367	EPI_ISL_256987
EPI_ISL_224062	EPI_ISL_201290	EPI_ISL_220360	EPI_ISL_286343	EPI_ISL_241577	EPI_ISL_240859
EPI_ISL_225107	EPI_ISL_202078	EPI_ISL_224038	EPI_ISL_286334	EPI_ISL_287444	EPI_ISL_259203
EPI_ISL_202915	EPI_ISL_202431	EPI_ISL_219184	EPI_ISL_249046	EPI_ISL_286712	EPI_ISL_239637
EPI_ISL_240160	EPI_ISL_203569	EPI_ISL_232528	EPI_ISL_244659	EPI_ISL_287254	EPI_ISL_239655
EPI_ISL_238623	EPI_ISL_202432	EPI_ISL_223360	EPI_ISL_239209	EPI_ISL_250037	EPI_ISL_240198
EPI_ISL_212163	EPI_ISL_203561	EPI_ISL_224064	EPI_ISL_284134	EPI_ISL_266705	EPI_ISL_239722
EPI_ISL_207392	EPI_ISL_203556	EPI_ISL_235115	EPI_ISL_286655	EPI_ISL_263434	EPI_ISL_240867
EPI_ISL_211906	EPI_ISL_220285	EPI_ISL_238642	EPI_ISL_256166	EPI_ISL_258341	EPI_ISL_259195
EPI_ISL_202095	EPI_ISL_205671	**Season 2016-17**	EPI_ISL_266681	EPI_ISL_256171	EPI_ISL_289219
EPI_ISL_222014	EPI_ISL_202094	EPI_ISL_294278	EPI_ISL_261842	EPI_ISL_239178	EPI_ISL_289243
EPI_ISL_202827	EPI_ISL_202824	EPI_ISL_329975	EPI_ISL_293412	EPI_ISL_287128	EPI_ISL_289322
EPI_ISL_207399	EPI_ISL_237293	EPI_ISL_253185	EPI_ISL_242744	EPI_ISL_287397	EPI_ISL_252738
EPI_ISL_230330	EPI_ISL_232103	EPI_ISL_252732	EPI_ISL_287306	EPI_ISL_327445	EPI_ISL_241632
EPI_ISL_214540	EPI_ISL_242718	EPI_ISL_286720	EPI_ISL_287368	EPI_ISL_253580	EPI_ISL_239208
EPI_ISL_222078	EPI_ISL_232552	EPI_ISL_258388	EPI_ISL_257856	EPI_ISL_259219	EPI_ISL_286373
EPI_ISL_213984	EPI_ISL_223166	EPI_ISL_286543	EPI_ISL_286516	EPI_ISL_238712	EPI_ISL_256958
EPI_ISL_212970	EPI_ISL_225138	EPI_ISL_286566	EPI_ISL_277277	EPI_ISL_287506	EPI_ISL_253603
EPI_ISL_225142	EPI_ISL_219234	EPI_ISL_286647	EPI_ISL_248139	EPI_ISL_287525	EPI_ISL_237444
EPI_ISL_219164	EPI_ISL_207412	EPI_ISL_286527	EPI_ISL_286716	EPI_ISL_239700	EPI_ISL_239644
EPI_ISL_219195	EPI_ISL_224050	EPI_ISL_286328	EPI_ISL_240178	EPI_ISL_289328	EPI_ISL_240862
EPI_ISL_208606	EPI_ISL_226829	EPI_ISL_286635	EPI_ISL_284165	EPI_ISL_275957	EPI_ISL_240168
EPI_ISL_225131	EPI_ISL_244911	EPI_ISL_286333	EPI_ISL_257846	EPI_ISL_256189	EPI_ISL_237335

EPI_ISL_287534	EPI_ISL_286514	EPI_ISL_320739	EPI_ISL_360921	EPI_ISL_361530	EPI_ISL_360092
EPI_ISL_239200	EPI_ISL_287421	EPI_ISL_306223	EPI_ISL_361367	EPI_ISL_300195	EPI_ISL_366984
EPI_ISL_239643	EPI_ISL_258292	EPI_ISL_289530	EPI_ISL_373406	EPI_ISL_312841	EPI_ISL_339853
EPI_ISL_239652	EPI_ISL_270167	EPI_ISL_308938	EPI_ISL_376645	EPI_ISL_320333	EPI_ISL_346221
EPI_ISL_241595	EPI_ISL_248099	EPI_ISL_284015	EPI_ISL_314348	EPI_ISL_305053	EPI_ISL_336554
EPI_ISL_286339	EPI_ISL_293410	EPI_ISL_300818	EPI_ISL_320729	EPI_ISL_300784	EPI_ISL_346247
EPI_ISL_283345	EPI_ISL_327414	EPI_ISL_298318	EPI_ISL_296123	EPI_ISL_303144	EPI_ISL_358972
EPI_ISL_281488	EPI_ISL_288276	EPI_ISL_360741	EPI_ISL_360839	EPI_ISL_286124	EPI_ISL_363913
EPI_ISL_256196	EPI_ISL_255322	EPI_ISL_315032	EPI_ISL_287581	EPI_ISL_290873	EPI_ISL_366468
EPI_ISL_286669	EPI_ISL_259108	EPI_ISL_360282	EPI_ISL_360435	EPI_ISL_287612	EPI_ISL_335605
EPI_ISL_248117	EPI_ISL_238719	EPI_ISL_361859	EPI_ISL_360436	EPI_ISL_286126	EPI_ISL_336548
EPI_ISL_248156	EPI_ISL_241621	EPI_ISL_308962	EPI_ISL_284065	EPI_ISL_287613	EPI_ISL_353481
EPI_ISL_255327	EPI_ISL_286510	EPI_ISL_306261	EPI_ISL_289767	EPI_ISL_287600	EPI_ISL_366924
EPI_ISL_240863	EPI_ISL_287443	EPI_ISL_295834	EPI_ISL_360266	**Season 2018-19**	EPI_ISL_370008
EPI_ISL_258394	EPI_ISL_241598	EPI_ISL_361474	EPI_ISL_295863	EPI_ISL_380974	EPI_ISL_353486
EPI_ISL_259095	EPI_ISL_266676	EPI_ISL_361220	EPI_ISL_288648	EPI_ISL_380981	EPI_ISL_359500
EPI_ISL_248007	**Season 2017-18**	EPI_ISL_361343	EPI_ISL_360451	EPI_ISL_380553	EPI_ISL_365851
EPI_ISL_287223	EPI_ISL_329971	EPI_ISL_373494	EPI_ISL_298331	EPI_ISL_366985	EPI_ISL_380663
EPI_ISL_287241	EPI_ISL_360513	EPI_ISL_376701	EPI_ISL_360491	EPI_ISL_501978	EPI_ISL_380664
EPI_ISL_262745	EPI_ISL_287594	EPI_ISL_361441	EPI_ISL_360293	EPI_ISL_349694	EPI_ISL_387465
EPI_ISL_266709	EPI_ISL_288525	EPI_ISL_360627	EPI_ISL_312904	EPI_ISL_357837	EPI_ISL_354161
EPI_ISL_270975	EPI_ISL_289731	EPI_ISL_358218	EPI_ISL_299923	EPI_ISL_380781	EPI_ISL_356252
EPI_ISL_287511	EPI_ISL_305285	EPI_ISL_319716	EPI_ISL_358240	EPI_ISL_368925	EPI_ISL_362582
EPI_ISL_289283	EPI_ISL_314410	EPI_ISL_312858	EPI_ISL_298340	EPI_ISL_360147	EPI_ISL_380764
EPI_ISL_264485	EPI_ISL_376846	EPI_ISL_309032	EPI_ISL_284033	EPI_ISL_380529	EPI_ISL_370059
EPI_ISL_281716	EPI_ISL_303097	EPI_ISL_303157	EPI_ISL_361392	EPI_ISL_366930	EPI_ISL_359488
EPI_ISL_239188	EPI_ISL_319730	EPI_ISL_330507	EPI_ISL_361256	EPI_ISL_369980	EPI_ISL_349710
EPI_ISL_287221	EPI_ISL_306880	EPI_ISL_331555	EPI_ISL_361408	EPI_ISL_347946	EPI_ISL_339743
EPI_ISL_286535	EPI_ISL_373457	EPI_ISL_358270	EPI_ISL_361245	EPI_ISL_349934	EPI_ISL_363846
EPI_ISL_275704	EPI_ISL_361585	EPI_ISL_300822	EPI_ISL_360393	EPI_ISL_351937	EPI_ISL_351994
EPI_ISL_272946	EPI_ISL_305050	EPI_ISL_376823	EPI_ISL_300821	EPI_ISL_358757	EPI_ISL_359497
EPI_ISL_282878	EPI_ISL_312859	EPI_ISL_327681	EPI_ISL_298326	EPI_ISL_362378	EPI_ISL_348007
EPI_ISL_277865	EPI_ISL_299968	EPI_ISL_361461	EPI_ISL_287603	EPI_ISL_362560	EPI_ISL_501945
EPI_ISL_281504	EPI_ISL_305830	EPI_ISL_360712	EPI_ISL_288450	EPI_ISL_360049	EPI_ISL_359433
EPI_ISL_286455	EPI_ISL_307894	EPI_ISL_360632	EPI_ISL_289734	EPI_ISL_360143	EPI_ISL_349754
EPI_ISL_273619	EPI_ISL_360913	EPI_ISL_360575	EPI_ISL_298319	EPI_ISL_372544	EPI_ISL_370638
EPI_ISL_266291	EPI_ISL_330494	EPI_ISL_312819	EPI_ISL_289741	EPI_ISL_334148	EPI_ISL_336536
EPI_ISL_240210	EPI_ISL_284032	EPI_ISL_309557	EPI_ISL_360270	EPI_ISL_349879	EPI_ISL_368998
EPI_ISL_247995	EPI_ISL_360901	EPI_ISL_303226	EPI_ISL_299439	EPI_ISL_358945	EPI_ISL_344691
EPI_ISL_257849	EPI_ISL_316778	EPI_ISL_295865	EPI_ISL_299503	EPI_ISL_359585	EPI_ISL_351914
EPI_ISL_256200	EPI_ISL_288656	EPI_ISL_290682	EPI_ISL_304302	EPI_ISL_366969	EPI_ISL_353446
EPI_ISL_287312	EPI_ISL_313687	EPI_ISL_327323	EPI_ISL_284034	EPI_ISL_368919	EPI_ISL_353478
EPI_ISL_266707	EPI_ISL_366206	EPI_ISL_360440	EPI_ISL_288458	EPI_ISL_369125	EPI_ISL_354211
EPI_ISL_258363	EPI_ISL_331803	EPI_ISL_320274	EPI_ISL_303118	EPI_ISL_372029	EPI_ISL_354227
EPI_ISL_239645	EPI_ISL_305049	EPI_ISL_360246	EPI_ISL_298595	EPI_ISL_372586	EPI_ISL_359023
EPI_ISL_249090	EPI_ISL_320752	EPI_ISL_360602	EPI_ISL_298566	EPI_ISL_344644	EPI_ISL_380639
EPI_ISL_256993	EPI_ISL_361720	EPI_ISL_360305	EPI_ISL_296120	EPI_ISL_359857	
EPI_ISL_241686	EPI_ISL_376610	EPI_ISL_286044	EPI_ISL_285678	EPI_ISL_359938	
EPI_ISL_258324	EPI_ISL_306246	EPI_ISL_296132	EPI_ISL_292593		
EPI_ISL_266701	EPI_ISL_373491	EPI_ISL_296150	EPI_ISL_373454		
EPI_ISL_240173	EPI_ISL_361230	EPI_ISL_298324	EPI_ISL_286133		
EPI_ISL_287255	EPI_ISL_360608	EPI_ISL_305032	EPI_ISL_360420		
EPI_ISL_250056	EPI_ISL_305301	EPI_ISL_305298	EPI_ISL_376587		
EPI_ISL_253615	EPI_ISL_360291	EPI_ISL_360879	EPI_ISL_361331		

EPI_ISL_502004	EPI_ISL_359027	EPI_ISL_380450	EPI_ISL_336744	EPI_ISL_372599	EPI_ISL_415774
EPI_ISL_505746	EPI_ISL_359414	EPI_ISL_366449	EPI_ISL_333762	EPI_ISL_380614	EPI_ISL_435325
EPI_ISL_360073	EPI_ISL_360152	EPI_ISL_368999	EPI_ISL_349721	EPI_ISL_334102	EPI_ISL_409121
EPI_ISL_360137	EPI_ISL_365848	EPI_ISL_502037	EPI_ISL_347919	EPI_ISL_334103	EPI_ISL_400025
EPI_ISL_351909	EPI_ISL_380985	EPI_ISL_354203	EPI_ISL_365841	EPI_ISL_335613	EPI_ISL_402743
EPI_ISL_360030	EPI_ISL_370137	EPI_ISL_353667	EPI_ISL_395148	EPI_ISL_336687	EPI_ISL_402758
EPI_ISL_349948	EPI_ISL_380843	EPI_ISL_358858	EPI_ISL_395433	EPI_ISL_359987	EPI_ISL_410760
EPI_ISL_362636	EPI_ISL_344626	EPI_ISL_359031	EPI_ISL_346216	EPI_ISL_334140	EPI_ISL_400836
EPI_ISL_352055	EPI_ISL_348158	EPI_ISL_333741	EPI_ISL_349937	EPI_ISL_349914	EPI_ISL_415762
EPI_ISL_335608	EPI_ISL_341042	EPI_ISL_366463	EPI_ISL_377938	EPI_ISL_332551	EPI_ISL_416775
EPI_ISL_357898	EPI_ISL_351956	EPI_ISL_353680	EPI_ISL_360148	EPI_ISL_331410	EPI_ISL_404760
EPI_ISL_359284	EPI_ISL_362417	EPI_ISL_359901	EPI_ISL_377886	EPI_ISL_344665	EPI_ISL_400873
EPI_ISL_344623	EPI_ISL_366819	EPI_ISL_362635	EPI_ISL_394926	**Season 2019-20**	EPI_ISL_410758
EPI_ISL_353707	EPI_ISL_354122	EPI_ISL_380882	EPI_ISL_400777	EPI_ISL_400881	EPI_ISL_410738
EPI_ISL_370087	EPI_ISL_380415	EPI_ISL_348176	EPI_ISL_354128	EPI_ISL_400994	EPI_ISL_414116
EPI_ISL_354219	EPI_ISL_505739	EPI_ISL_369126	EPI_ISL_355751	EPI_ISL_402445	EPI_ISL_424755
EPI_ISL_360142	EPI_ISL_359892	EPI_ISL_366713	EPI_ISL_365821	EPI_ISL_396999	EPI_ISL_409048
EPI_ISL_334146	EPI_ISL_380409	EPI_ISL_369894	EPI_ISL_359558	EPI_ISL_398548	EPI_ISL_398829
EPI_ISL_336515	EPI_ISL_380736	EPI_ISL_348233	EPI_ISL_377888	EPI_ISL_397195	EPI_ISL_397228
EPI_ISL_338594	EPI_ISL_369099	EPI_ISL_359029	EPI_ISL_398835	EPI_ISL_415687	EPI_ISL_398800
EPI_ISL_338597	EPI_ISL_351948	EPI_ISL_359922	EPI_ISL_393791	EPI_ISL_410752	EPI_ISL_410733
EPI_ISL_336564	EPI_ISL_362641	EPI_ISL_362349	EPI_ISL_395408	EPI_ISL_412157	EPI_ISL_413261
EPI_ISL_344705	EPI_ISL_363835	EPI_ISL_370018	EPI_ISL_395427	EPI_ISL_415771	EPI_ISL_404543
EPI_ISL_349913	EPI_ISL_365985	EPI_ISL_370203	EPI_ISL_391368	EPI_ISL_400817	EPI_ISL_410755
EPI_ISL_349946	EPI_ISL_380783	EPI_ISL_380549	EPI_ISL_393775	EPI_ISL_416788	EPI_ISL_409385
EPI_ISL_353635	EPI_ISL_369039	EPI_ISL_331864	EPI_ISL_363856	EPI_ISL_449732	
EPI_ISL_354174	EPI_ISL_355723	EPI_ISL_335612	EPI_ISL_398955	EPI_ISL_482891	
EPI_ISL_355575	EPI_ISL_359152	EPI_ISL_332558	EPI_ISL_336484	EPI_ISL_455806	
EPI_ISL_358934	EPI_ISL_360088	EPI_ISL_333750	EPI_ISL_387496	EPI_ISL_409391	

Selected from Ireland (n=112)

**Season 2016-17**	EPI_ISL_267720	EPI_ISL_308723	**Season 2018-19**	EPI_ISL_393995	EPI_ISL_344492
EPI_ISL_267726	EPI_ISL_267723	EPI_ISL_321850	EPI_ISL_344506	EPI_ISL_344502	EPI_ISL_344491
EPI_ISL_267732	EPI_ISL_256029	EPI_ISL_321848	EPI_ISL_393996	EPI_ISL_344500	**Season 2019-20**
EPI_ISL_267729	EPI_ISL_267721	EPI_ISL_299042	EPI_ISL_394007	EPI_ISL_356557	EPI_ISL_406189
EPI_ISL_267722	EPI_ISL_256026	EPI_ISL_299041	EPI_ISL_394005	EPI_ISL_394001	EPI_ISL_406190
EPI_ISL_267719	EPI_ISL_267749	EPI_ISL_299040	EPI_ISL_393999	EPI_ISL_394004	EPI_ISL_403306
EPI_ISL_256025	**Season 2017-18**	EPI_ISL_297340	EPI_ISL_394002	EPI_ISL_344499	EPI_ISL_407489
EPI_ISL_267730	EPI_ISL_329775	EPI_ISL_297339	EPI_ISL_356549	EPI_ISL_344498	EPI_ISL_426207
EPI_ISL_267725	EPI_ISL_300412	EPI_ISL_308721	EPI_ISL_394003	EPI_ISL_337040	EPI_ISL_409985
EPI_ISL_267731	EPI_ISL_321849	EPI_ISL_329777	EPI_ISL_389211	EPI_ISL_337904	EPI_ISL_403295
EPI_ISL_256030	EPI_ISL_329780	EPI_ISL_308720	EPI_ISL_344505	EPI_ISL_337903	EPI_ISL_403302
EPI_ISL_267728	EPI_ISL_329778	EPI_ISL_299043	EPI_ISL_344494	EPI_ISL_336094	EPI_ISL_406187
EPI_ISL_267727	EPI_ISL_329776	EPI_ISL_308722	EPI_ISL_336115	EPI_ISL_336093	EPI_ISL_406192
EPI_ISL_267733	EPI_ISL_329774	EPI_ISL_329779	EPI_ISL_337902	EPI_ISL_394006	EPI_ISL_426210
EPI_ISL_267724	EPI_ISL_321847		EPI_ISL_336092	EPI_ISL_356547	EPI_ISL_426213
EPI_ISL_256027			EPI_ISL_393998	EPI_ISL_344495	
EPI_ISL_256028			EPI_ISL_356550	EPI_ISL_393997	

EPI_ISL_426208	EPI_ISL_403288	EPI_ISL_426212	EPI_ISL_403289	EPI_ISL_426214	EPI_ISL_426209
EPI_ISL_406193	EPI_ISL_527231	EPI_ISL_403290	EPI_ISL_403300	EPI_ISL_403301	EPI_ISL_406194
EPI_ISL_403291	EPI_ISL_407488	EPI_ISL_403303	EPI_ISL_407487	EPI_ISL_426206	
EPI_ISL_406191	EPI_ISL_403304	EPI_ISL_426211	EPI_ISL_407486	EPI_ISL_406188	

Selected from Netherlands (n=800)

Season 2015-16

- EPI_ISL_355168
- EPI_ISL_230723
- EPI_ISL_230717
- EPI_ISL_216008
- EPI_ISL_216007
- EPI_ISL_233371

Season 2016-17

- EPI_ISL_255649
- EPI_ISL_316672
- EPI_ISL_253138
- EPI_ISL_255644
- EPI_ISL_247420
- EPI_ISL_270971
- EPI_ISL_355173
- EPI_ISL_241355
- EPI_ISL_253132
- EPI_ISL_241365
- EPI_ISL_259986
- EPI_ISL_247424
- EPI_ISL_241356
- EPI_ISL_259072
- EPI_ISL_240591
- EPI_ISL_291988
- EPI_ISL_241351
- EPI_ISL_253133
- EPI_ISL_249661
- EPI_ISL_241348
- EPI_ISL_240593
- EPI_ISL_270970
- EPI_ISL_258248
- EPI_ISL_255664
- EPI_ISL_308728
- EPI_ISL_293135
- EPI_ISL_242388
- EPI_ISL_240588
- EPI_ISL_253131
- EPI_ISL_241364
- EPI_ISL_241359
- EPI_ISL_240589
- EPI_ISL_247423
- EPI_ISL_253139
- EPI_ISL_241362
- EPI_ISL_247428
- EPI_ISL_247419
- EPI_ISL_316673
- EPI_ISL_247426
- EPI_ISL_242382
- EPI_ISL_249658
- EPI_ISL_241353
- EPI_ISL_240594
- EPI_ISL_253134
- EPI_ISL_241350
- EPI_ISL_253136
- EPI_ISL_241357
- EPI_ISL_253135
- EPI_ISL_249667
- EPI_ISL_241360
- EPI_ISL_241352
- EPI_ISL_242386
- EPI_ISL_255641
- EPI_ISL_253130
- EPI_ISL_241347
- EPI_ISL_240590
- EPI_ISL_253137
- EPI_ISL_241358
- EPI_ISL_242387
- EPI_ISL_249663
- EPI_ISL_241354
- EPI_ISL_240587
- EPI_ISL_291989
- EPI_ISL_291990
- EPI_ISL_241349
- EPI_ISL_249659
- EPI_ISL_255639
- EPI_ISL_249662
- EPI_ISL_247417
- EPI_ISL_241363

Season 2017-18

- EPI_ISL_309604
- EPI_ISL_309308
- EPI_ISL_301261
- EPI_ISL_305532
- EPI_ISL_309409
- EPI_ISL_305419
- EPI_ISL_309407
- EPI_ISL_291991
- EPI_ISL_309303
- EPI_ISL_309304
- EPI_ISL_293131
- EPI_ISL_309410
- EPI_ISL_305418
- EPI_ISL_316674
- EPI_ISL_305426
- EPI_ISL_309406
- EPI_ISL_305422
- EPI_ISL_304568
- EPI_ISL_305425
- EPI_ISL_305427
- EPI_ISL_309311
- EPI_ISL_302338
- EPI_ISL_305513
- EPI_ISL_301287
- EPI_ISL_309301
- EPI_ISL_309609
- EPI_ISL_306979
- EPI_ISL_306988
- EPI_ISL_309603
- EPI_ISL_291966
- EPI_ISL_305506
- EPI_ISL_309610
- EPI_ISL_305516
- EPI_ISL_305497
- EPI_ISL_305494
- EPI_ISL_305504
- EPI_ISL_305499
- EPI_ISL_309607
- EPI_ISL_309606
- EPI_ISL_309608
- EPI_ISL_301295
- EPI_ISL_293130
- EPI_ISL_301300
- EPI_ISL_305498
- EPI_ISL_291938
- EPI_ISL_305503
- EPI_ISL_301286
- EPI_ISL_304567
- EPI_ISL_304566
- EPI_ISL_305420
- EPI_ISL_309309
- EPI_ISL_301264
- EPI_ISL_305500
- EPI_ISL_309305
- EPI_ISL_293132
- EPI_ISL_309605
- EPI_ISL_306986
- EPI_ISL_334099
- EPI_ISL_348116
- EPI_ISL_336557
- EPI_ISL_305501
- EPI_ISL_302340
- EPI_ISL_306977
- EPI_ISL_305505
- EPI_ISL_302032
- EPI_ISL_309306
- EPI_ISL_309307
- EPI_ISL_309310
- EPI_ISL_293134
- EPI_ISL_293133
- EPI_ISL_305424
- EPI_ISL_309408
- EPI_ISL_309405
- EPI_ISL_291933
- EPI_ISL_291932
- EPI_ISL_305421
- EPI_ISL_316675
- EPI_ISL_305423

Season 2018-19

- EPI_ISL_355165
- EPI_ISL_339299
- EPI_ISL_340919
- EPI_ISL_352421
- EPI_ISL_342118
- EPI_ISL_351174
- EPI_ISL_347633
- EPI_ISL_347546
- EPI_ISL_342158
- EPI_ISL_355138
- EPI_ISL_347560
- EPI_ISL_347580
- EPI_ISL_343215
- EPI_ISL_343244
- EPI_ISL_354023
- EPI_ISL_351327
- EPI_ISL_355163
- EPI_ISL_355162
- EPI_ISL_339536
- EPI_ISL_345720
- EPI_ISL_347565
- EPI_ISL_350542
- EPI_ISL_347571
- EPI_ISL_340298
- EPI_ISL_340913
- EPI_ISL_339310
- EPI_ISL_347549
- EPI_ISL_347556
- EPI_ISL_347566
- EPI_ISL_347538
- EPI_ISL_343213
- EPI_ISL_351333
- EPI_ISL_354001
- EPI_ISL_355136
- EPI_ISL_356891
- EPI_ISL_340305
- EPI_ISL_345946
- EPI_ISL_342096
- EPI_ISL_335584
- EPI_ISL_339372
- EPI_ISL_349450
- EPI_ISL_352423
- EPI_ISL_342097
- EPI_ISL_343352
- EPI_ISL_336268
- EPI_ISL_350534
- EPI_ISL_337181
- EPI_ISL_340940
- EPI_ISL_343223
- EPI_ISL_355142
- EPI_ISL_334254
- EPI_ISL_336169
- EPI_ISL_336190
- EPI_ISL_336193
- EPI_ISL_336188
- EPI_ISL_343335
- EPI_ISL_339540
- EPI_ISL_339523
- EPI_ISL_336170
- EPI_ISL_343326
- EPI_ISL_343267
- EPI_ISL_339550
- EPI_ISL_339542
- EPI_ISL_342153
- EPI_ISL_334243
- EPI_ISL_340515
- EPI_ISL_337182
- EPI_ISL_343214
- EPI_ISL_339305
- EPI_ISL_337199
- EPI_ISL_370374
- EPI_ISL_370378
- EPI_ISL_356888
- EPI_ISL_350531
- EPI_ISL_355166
- EPI_ISL_355170
- EPI_ISL_350667
- EPI_ISL_351337
- EPI_ISL_354013
- EPI_ISL_351345
- EPI_ISL_356889
- EPI_ISL_356879
- EPI_ISL_343239
- EPI_ISL_343259
- EPI_ISL_340932
- EPI_ISL_356890
- EPI_ISL_354021
- EPI_ISL_354007
- EPI_ISL_349471
- EPI_ISL_350665
- EPI_ISL_355159
- EPI_ISL_351330
- EPI_ISL_342114
- EPI_ISL_347634
- EPI_ISL_339378
- EPI_ISL_344867
- EPI_ISL_342098
- EPI_ISL_349474
- EPI_ISL_345947
- EPI_ISL_340287
- EPI_ISL_339371
- EPI_ISL_349465
- EPI_ISL_350539
- EPI_ISL_336197

EPI_ISL_345712	EPI_ISL_342157	EPI_ISL_340922	EPI_ISL_340912	EPI_ISL_350666	EPI_ISL_354016
EPI_ISL_347545	EPI_ISL_395133	EPI_ISL_339291	EPI_ISL_339539	EPI_ISL_342122	EPI_ISL_351335
EPI_ISL_347596	EPI_ISL_351325	EPI_ISL_340916	EPI_ISL_340508	EPI_ISL_334166	EPI_ISL_343245
EPI_ISL_351344	EPI_ISL_347600	EPI_ISL_347548	EPI_ISL_339294	EPI_ISL_337193	EPI_ISL_347598
EPI_ISL_350545	EPI_ISL_340307	EPI_ISL_354012	EPI_ISL_342137	EPI_ISL_339308	EPI_ISL_342166
EPI_ISL_347553	EPI_ISL_351324	EPI_ISL_345722	EPI_ISL_339298	EPI_ISL_340315	EPI_ISL_342140
EPI_ISL_347547	EPI_ISL_350669	EPI_ISL_339304	EPI_ISL_356883	EPI_ISL_340301	EPI_ISL_342156
EPI_ISL_347555	EPI_ISL_351177	EPI_ISL_342141	EPI_ISL_356882	EPI_ISL_345724	EPI_ISL_339309
EPI_ISL_347541	EPI_ISL_351176	EPI_ISL_337190	EPI_ISL_347554	EPI_ISL_350543	EPI_ISL_343216
EPI_ISL_347568	EPI_ISL_342152	EPI_ISL_343241	EPI_ISL_336182	EPI_ISL_335565	EPI_ISL_356886
EPI_ISL_347567	EPI_ISL_355150	EPI_ISL_343255	EPI_ISL_343242	EPI_ISL_354015	EPI_ISL_342143
EPI_ISL_345711	EPI_ISL_355131	EPI_ISL_347579	EPI_ISL_344864	EPI_ISL_345726	EPI_ISL_343243
EPI_ISL_345703	EPI_ISL_343252	EPI_ISL_343251	EPI_ISL_344862	EPI_ISL_354010	EPI_ISL_343230
EPI_ISL_345710	EPI_ISL_340308	EPI_ISL_336180	EPI_ISL_343351	EPI_ISL_347597	EPI_ISL_345719
EPI_ISL_345709	EPI_ISL_344870	EPI_ISL_340506	EPI_ISL_343367	EPI_ISL_336175	EPI_ISL_356887
EPI_ISL_354005	EPI_ISL_349479	EPI_ISL_340504	EPI_ISL_343378	EPI_ISL_340499	EPI_ISL_355171
EPI_ISL_350548	EPI_ISL_344866	EPI_ISL_340310	EPI_ISL_339379	EPI_ISL_336189	EPI_ISL_355160
EPI_ISL_350546	EPI_ISL_339300	EPI_ISL_355143	EPI_ISL_349495	EPI_ISL_335567	EPI_ISL_337198
EPI_ISL_350544	EPI_ISL_354011	EPI_ISL_340295	EPI_ISL_339377	EPI_ISL_334255	EPI_ISL_343248
EPI_ISL_347589	EPI_ISL_355161	EPI_ISL_339376	EPI_ISL_342124	EPI_ISL_347587	EPI_ISL_343249
EPI_ISL_347592	EPI_ISL_347594	EPI_ISL_345721	EPI_ISL_342011	EPI_ISL_343332	EPI_ISL_354008
EPI_ISL_342138	EPI_ISL_355157	EPI_ISL_340920	EPI_ISL_335587	EPI_ISL_347588	EPI_ISL_351341
EPI_ISL_355164	EPI_ISL_355156	EPI_ISL_340918	EPI_ISL_335571	EPI_ISL_356880	EPI_ISL_343253
EPI_ISL_340923	EPI_ISL_355145	EPI_ISL_340510	EPI_ISL_340939	EPI_ISL_343237	EPI_ISL_351338
EPI_ISL_347585	EPI_ISL_343211	EPI_ISL_343358	EPI_ISL_355167	EPI_ISL_355132	EPI_ISL_342100
EPI_ISL_347599	EPI_ISL_355140	EPI_ISL_345725	EPI_ISL_351328	EPI_ISL_354009	EPI_ISL_335582
EPI_ISL_354014	EPI_ISL_355152	EPI_ISL_342155	EPI_ISL_342148	EPI_ISL_351340	EPI_ISL_334163
EPI_ISL_354019	EPI_ISL_355135	EPI_ISL_342154	EPI_ISL_339289	EPI_ISL_337211	EPI_ISL_345943
EPI_ISL_356881	EPI_ISL_355141	EPI_ISL_336202	EPI_ISL_335560	EPI_ISL_340312	**Season 2019-20**
EPI_ISL_356884	EPI_ISL_349494	EPI_ISL_340500	EPI_ISL_340915	EPI_ISL_340914	EPI_ISL_415726
EPI_ISL_342131	EPI_ISL_349469	EPI_ISL_335561	EPI_ISL_343322	EPI_ISL_349463	EPI_ISL_401965
EPI_ISL_336198	EPI_ISL_343354	EPI_ISL_335557	EPI_ISL_336184	EPI_ISL_351323	EPI_ISL_416275
EPI_ISL_340938	EPI_ISL_342121	EPI_ISL_342159	EPI_ISL_345715	EPI_ISL_354022	EPI_ISL_421643
EPI_ISL_336199	EPI_ISL_340304	EPI_ISL_339290	EPI_ISL_345716	EPI_ISL_342130	EPI_ISL_412432
EPI_ISL_347586	EPI_ISL_395130	EPI_ISL_340302	EPI_ISL_340503	EPI_ISL_350528	EPI_ISL_406858
EPI_ISL_356885	EPI_ISL_331830	EPI_ISL_340294	EPI_ISL_337195	EPI_ISL_335563	EPI_ISL_414609
EPI_ISL_342139	EPI_ISL_355155	EPI_ISL_347601	EPI_ISL_343232	EPI_ISL_335558	EPI_ISL_416563
EPI_ISL_342133	EPI_ISL_355154	EPI_ISL_340525	EPI_ISL_339538	EPI_ISL_336181	EPI_ISL_400505
EPI_ISL_334249	EPI_ISL_342135	EPI_ISL_343329	EPI_ISL_339303	EPI_ISL_339545	EPI_ISL_415715
EPI_ISL_351331	EPI_ISL_339527	EPI_ISL_343271	EPI_ISL_340917	EPI_ISL_345707	EPI_ISL_415714
EPI_ISL_351343	EPI_ISL_337186	EPI_ISL_336174	EPI_ISL_343210	EPI_ISL_354006	EPI_ISL_415713
EPI_ISL_354020	EPI_ISL_347636	EPI_ISL_355139	EPI_ISL_343257	EPI_ISL_355153	EPI_ISL_408499
EPI_ISL_335562	EPI_ISL_340297	EPI_ISL_342117	EPI_ISL_342160	EPI_ISL_355149	EPI_ISL_401969
EPI_ISL_337205	EPI_ISL_342115	EPI_ISL_340309	EPI_ISL_334250	EPI_ISL_355147	EPI_ISL_413972
EPI_ISL_343235	EPI_ISL_342099	EPI_ISL_339293	EPI_ISL_340509	EPI_ISL_342113	EPI_ISL_413971
EPI_ISL_351348	EPI_ISL_336265	EPI_ISL_336178	EPI_ISL_351326	EPI_ISL_343357	EPI_ISL_411942
EPI_ISL_347581	EPI_ISL_350552	EPI_ISL_342010	EPI_ISL_340517	EPI_ISL_345944	EPI_ISL_402107
EPI_ISL_354018	EPI_ISL_347540	EPI_ISL_335569	EPI_ISL_340909	EPI_ISL_342125	EPI_ISL_403747
EPI_ISL_342164	EPI_ISL_347542	EPI_ISL_347570	EPI_ISL_355133	EPI_ISL_352424	EPI_ISL_416557
EPI_ISL_340505	EPI_ISL_345717	EPI_ISL_340924	EPI_ISL_337180	EPI_ISL_343353	EPI_ISL_413525
EPI_ISL_337207	EPI_ISL_343218	EPI_ISL_339306	EPI_ISL_350551	EPI_ISL_342126	EPI_ISL_411937
EPI_ISL_337200	EPI_ISL_337184	EPI_ISL_331832	EPI_ISL_347551	EPI_ISL_340904	EPI_ISL_413549
EPI_ISL_336192	EPI_ISL_350529	EPI_ISL_334245	EPI_ISL_351172	EPI_ISL_350550	EPI_ISL_414604
EPI_ISL_342162	EPI_ISL_354024	EPI_ISL_334164	EPI_ISL_350668	EPI_ISL_343247	EPI_ISL_414602

EPI_ISL_402106	EPI_ISL_404140	EPI_ISL_410400	EPI_ISL_403754	EPI_ISL_410388	EPI_ISL_408491
EPI_ISL_410008	EPI_ISL_404137	EPI_ISL_412427	EPI_ISL_403755	EPI_ISL_401980	EPI_ISL_414607
EPI_ISL_410011	EPI_ISL_402114	EPI_ISL_412440	EPI_ISL_403756	EPI_ISL_408502	EPI_ISL_408510
EPI_ISL_416280	EPI_ISL_402105	EPI_ISL_412453	EPI_ISL_404143	EPI_ISL_403758	EPI_ISL_403760
EPI_ISL_416279	EPI_ISL_402104	EPI_ISL_415712	EPI_ISL_408492	EPI_ISL_403757	EPI_ISL_401978
EPI_ISL_416281	EPI_ISL_402103	EPI_ISL_415725	EPI_ISL_408508	EPI_ISL_415732	EPI_ISL_415738
EPI_ISL_416282	EPI_ISL_408393	EPI_ISL_415716	EPI_ISL_406882	EPI_ISL_408392	EPI_ISL_408396
EPI_ISL_400507	EPI_ISL_406853	EPI_ISL_415727	EPI_ISL_403753	EPI_ISL_406847	EPI_ISL_408391
EPI_ISL_405407	EPI_ISL_416273	EPI_ISL_415728	EPI_ISL_410396	EPI_ISL_416277	EPI_ISL_401981
EPI_ISL_413533	EPI_ISL_416560	EPI_ISL_415729	EPI_ISL_412442	EPI_ISL_395172	EPI_ISL_410383
EPI_ISL_421646	EPI_ISL_415717	EPI_ISL_415730	EPI_ISL_406869	EPI_ISL_416552	EPI_ISL_410395
EPI_ISL_403761	EPI_ISL_416548	EPI_ISL_401983	EPI_ISL_406881	EPI_ISL_413537	EPI_ISL_412449
EPI_ISL_400587	EPI_ISL_405396	EPI_ISL_415731	EPI_ISL_406884	EPI_ISL_411947	EPI_ISL_413969
EPI_ISL_400290	EPI_ISL_406848	EPI_ISL_415721	EPI_ISL_408387	EPI_ISL_411940	EPI_ISL_412431
EPI_ISL_421650	EPI_ISL_412430	EPI_ISL_401975	EPI_ISL_408397	EPI_ISL_404145	EPI_ISL_413962
EPI_ISL_413532	EPI_ISL_401974	EPI_ISL_412446	EPI_ISL_410067	EPI_ISL_405720	EPI_ISL_410077
EPI_ISL_408507	EPI_ISL_410064	EPI_ISL_401970	EPI_ISL_410085	EPI_ISL_416551	EPI_ISL_410074
EPI_ISL_400508	EPI_ISL_413547	EPI_ISL_403759	EPI_ISL_408395	EPI_ISL_410381	EPI_ISL_412457
EPI_ISL_400506	EPI_ISL_413535	EPI_ISL_403764	EPI_ISL_408390	EPI_ISL_410397	EPI_ISL_412429
EPI_ISL_415734	EPI_ISL_413540	EPI_ISL_405402	EPI_ISL_406888	EPI_ISL_410072	EPI_ISL_405400
EPI_ISL_400588	EPI_ISL_401973	EPI_ISL_416554	EPI_ISL_406879	EPI_ISL_413964	EPI_ISL_406889
EPI_ISL_402110	EPI_ISL_403750	EPI_ISL_406851	EPI_ISL_406873	EPI_ISL_413959	EPI_ISL_415719
EPI_ISL_405723	EPI_ISL_403762	EPI_ISL_406856	EPI_ISL_406870	EPI_ISL_412452	EPI_ISL_413957
EPI_ISL_407060	EPI_ISL_415722	EPI_ISL_406878	EPI_ISL_401972	EPI_ISL_410384	EPI_ISL_410010
EPI_ISL_411943	EPI_ISL_415723	EPI_ISL_406872	EPI_ISL_412438	EPI_ISL_403763	EPI_ISL_397929
EPI_ISL_413541	EPI_ISL_405722	EPI_ISL_406855	EPI_ISL_412428	EPI_ISL_406846	EPI_ISL_407065
EPI_ISL_400586	EPI_ISL_416556	EPI_ISL_406852	EPI_ISL_410393	EPI_ISL_408389	EPI_ISL_411941
EPI_ISL_421647	EPI_ISL_406883	EPI_ISL_405403	EPI_ISL_406890	EPI_ISL_406877	EPI_ISL_402109
EPI_ISL_411939	EPI_ISL_408386	EPI_ISL_405392	EPI_ISL_398158	EPI_ISL_406857	EPI_ISL_410385
EPI_ISL_411948	EPI_ISL_410093	EPI_ISL_403766	EPI_ISL_416278	EPI_ISL_415718	EPI_ISL_405401
EPI_ISL_411946	EPI_ISL_413966	EPI_ISL_403749	EPI_ISL_398159	EPI_ISL_405393	EPI_ISL_410069
EPI_ISL_408509	EPI_ISL_413967	EPI_ISL_413963	EPI_ISL_412439	EPI_ISL_402108	EPI_ISL_401977
EPI_ISL_408503	EPI_ISL_405395	EPI_ISL_410082	EPI_ISL_406859	EPI_ISL_410009	EPI_ISL_408388
EPI_ISL_408496	EPI_ISL_410061	EPI_ISL_413960	EPI_ISL_401966	EPI_ISL_412441	EPI_ISL_412456
EPI_ISL_408495	EPI_ISL_410387	EPI_ISL_401971	EPI_ISL_405397	EPI_ISL_410386	
EPI_ISL_407062	EPI_ISL_410390	EPI_ISL_403748	EPI_ISL_401967	EPI_ISL_405399	
EPI_ISL_405719	EPI_ISL_410391	EPI_ISL_403752	EPI_ISL_404141	EPI_ISL_405718	

Selected from Sweden (n=590)

**Season 2015-16**	EPI_ISL_205767	EPI_ISL_215741	EPI_ISL_215738	**Season 2016-17**	EPI_ISL_239538
EPI_ISL_215753	EPI_ISL_205771	EPI_ISL_205790	EPI_ISL_215721	EPI_ISL_241921	EPI_ISL_239547
EPI_ISL_205784	EPI_ISL_205774	EPI_ISL_205782	EPI_ISL_220477	EPI_ISL_241929	EPI_ISL_239539
EPI_ISL_215720	EPI_ISL_205826	EPI_ISL_208206	EPI_ISL_220479	EPI_ISL_253865	EPI_ISL_258913
EPI_ISL_231826	EPI_ISL_206414	EPI_ISL_220465	EPI_ISL_220435	EPI_ISL_234033	EPI_ISL_258826
EPI_ISL_215719	EPI_ISL_208207	EPI_ISL_226094	EPI_ISL_220458	EPI_ISL_261945	EPI_ISL_245011
EPI_ISL_231827	EPI_ISL_205770	EPI_ISL_220472	EPI_ISL_205783	EPI_ISL_258813	EPI_ISL_248876
EPI_ISL_220452	EPI_ISL_201101	EPI_ISL_226095	EPI_ISL_220449	EPI_ISL_257398	EPI_ISL_241935
EPI_ISL_220454	EPI_ISL_201103	EPI_ISL_220457	EPI_ISL_226093	EPI_ISL_253958	EPI_ISL_241920
EPI_ISL_208205	EPI_ISL_234034	EPI_ISL_233407	EPI_ISL_202344	EPI_ISL_253148	EPI_ISL_239529
EPI_ISL_208204	EPI_ISL_220447	EPI_ISL_205781	EPI_ISL_213095	EPI_ISL_253146	EPI_ISL_239530
EPI_ISL_205778	EPI_ISL_220450	EPI_ISL_233408	EPI_ISL_199503	EPI_ISL_241907	EPI_ISL_241899
EPI_ISL_205772	EPI_ISL_226092	EPI_ISL_220448	EPI_ISL_201102	EPI_ISL_239537	EPI_ISL_241900
EPI_ISL_205766	EPI_ISL_215718	EPI_ISL_215722	EPI_ISL_201100	EPI_ISL_241914	EPI_ISL_241902

EPI_ISL_245009	EPI_ISL_253891	EPI_ISL_237515	EPI_ISL_241934	EPI_ISL_282969	EPI_ISL_285976
EPI_ISL_245013	EPI_ISL_253917	EPI_ISL_237516	EPI_ISL_261689	EPI_ISL_282974	EPI_ISL_285979
EPI_ISL_245014	EPI_ISL_258809	EPI_ISL_241912	EPI_ISL_274117	EPI_ISL_310247	EPI_ISL_310243
EPI_ISL_245016	EPI_ISL_258817	EPI_ISL_258927	EPI_ISL_285506	EPI_ISL_282971	EPI_ISL_307083
EPI_ISL_248875	EPI_ISL_258820	EPI_ISL_258804	EPI_ISL_263021	EPI_ISL_302173	EPI_ISL_307079
EPI_ISL_258811	EPI_ISL_261639	EPI_ISL_237508	EPI_ISL_239535	EPI_ISL_314665	EPI_ISL_292065
EPI_ISL_258814	EPI_ISL_261650	EPI_ISL_237512	EPI_ISL_239548	EPI_ISL_302283	EPI_ISL_292057
EPI_ISL_258818	EPI_ISL_261653	EPI_ISL_239532	EPI_ISL_241915	EPI_ISL_314683	EPI_ISL_291379
EPI_ISL_239546	EPI_ISL_261932	EPI_ISL_263032	EPI_ISL_253149	EPI_ISL_310262	EPI_ISL_291376
EPI_ISL_239549	EPI_ISL_261941	EPI_ISL_261654	EPI_ISL_253869	EPI_ISL_310245	EPI_ISL_282985
EPI_ISL_241911	EPI_ISL_274121	EPI_ISL_261940	EPI_ISL_241926	EPI_ISL_310255	EPI_ISL_282977
EPI_ISL_258911	EPI_ISL_241930	EPI_ISL_258832	EPI_ISL_281576	EPI_ISL_314684	EPI_ISL_292918
EPI_ISL_263022	EPI_ISL_274123	EPI_ISL_258835	EPI_ISL_292920	EPI_ISL_282973	EPI_ISL_285991
EPI_ISL_261667	EPI_ISL_258918	EPI_ISL_253859	EPI_ISL_241924	EPI_ISL_302286	EPI_ISL_285992
EPI_ISL_258920	EPI_ISL_245023	EPI_ISL_245017	EPI_ISL_241925	EPI_ISL_302184	EPI_ISL_285993
EPI_ISL_261942	EPI_ISL_241905	EPI_ISL_241931	EPI_ISL_241933	EPI_ISL_292922	EPI_ISL_289495
EPI_ISL_258929	EPI_ISL_245008	EPI_ISL_253879	EPI_ISL_241932	EPI_ISL_285973	EPI_ISL_291373
EPI_ISL_261644	EPI_ISL_245010	EPI_ISL_261662	EPI_ISL_258915	EPI_ISL_285980	EPI_ISL_292900
EPI_ISL_274105	EPI_ISL_248874	EPI_ISL_258833	EPI_ISL_258917	EPI_ISL_285988	EPI_ISL_292901
EPI_ISL_263028	EPI_ISL_257402	EPI_ISL_253956	EPI_ISL_253868	EPI_ISL_291397	EPI_ISL_292902
EPI_ISL_258925	EPI_ISL_245007	EPI_ISL_253147	EPI_ISL_258921	EPI_ISL_291398	EPI_ISL_297352
EPI_ISL_261646	EPI_ISL_245012	EPI_ISL_239542	EPI_ISL_253880	EPI_ISL_291399	EPI_ISL_302181
EPI_ISL_258805	EPI_ISL_248873	EPI_ISL_253873	EPI_ISL_239555	EPI_ISL_292036	EPI_ISL_302287
EPI_ISL_261663	EPI_ISL_248877	EPI_ISL_253898	EPI_ISL_241936	EPI_ISL_292071	EPI_ISL_310250
EPI_ISL_263029	EPI_ISL_253953	EPI_ISL_253870	EPI_ISL_245006	EPI_ISL_292046	EPI_ISL_302269
EPI_ISL_261939	EPI_ISL_258828	EPI_ISL_274122	EPI_ISL_257401	EPI_ISL_292047	EPI_ISL_310261
EPI_ISL_253895	EPI_ISL_281230	EPI_ISL_274118	EPI_ISL_258822	EPI_ISL_307088	EPI_ISL_307090
EPI_ISL_261690	EPI_ISL_245025	EPI_ISL_274120	EPI_ISL_241909	EPI_ISL_307089	EPI_ISL_310260
EPI_ISL_258919	EPI_ISL_253858	EPI_ISL_253150	EPI_ISL_253892	EPI_ISL_310244	EPI_ISL_310254
EPI_ISL_245005	EPI_ISL_253861	EPI_ISL_241923	EPI_ISL_245018	EPI_ISL_310252	EPI_ISL_310249
EPI_ISL_248872	EPI_ISL_261944	EPI_ISL_241916	EPI_ISL_258806	EPI_ISL_310253	EPI_ISL_307072
EPI_ISL_241940	EPI_ISL_261656	EPI_ISL_241908	EPI_ISL_261688	EPI_ISL_310263	EPI_ISL_307084
EPI_ISL_263026	EPI_ISL_274106	EPI_ISL_241906	**Season 2017-18**	EPI_ISL_282976	EPI_ISL_307092
EPI_ISL_261661	EPI_ISL_258926	EPI_ISL_235390	EPI_ISL_307077	EPI_ISL_282982	EPI_ISL_310256
EPI_ISL_261642	EPI_ISL_258834	EPI_ISL_241904	EPI_ISL_307078	EPI_ISL_282983	EPI_ISL_285989
EPI_ISL_261637	EPI_ISL_258928	EPI_ISL_241922	EPI_ISL_310242	EPI_ISL_282975	EPI_ISL_285990
EPI_ISL_274116	EPI_ISL_258819	EPI_ISL_241927	EPI_ISL_314682	EPI_ISL_285981	EPI_ISL_314659
EPI_ISL_258922	EPI_ISL_258816	EPI_ISL_241937	EPI_ISL_291392	EPI_ISL_307091	EPI_ISL_281232
EPI_ISL_258831	EPI_ISL_253889	EPI_ISL_245019	EPI_ISL_282981	EPI_ISL_282984	EPI_ISL_282970
EPI_ISL_258812	EPI_ISL_253894	EPI_ISL_245020	EPI_ISL_292924	EPI_ISL_285982	EPI_ISL_285986
EPI_ISL_253888	EPI_ISL_261648	EPI_ISL_253863	EPI_ISL_307080	EPI_ISL_291374	EPI_ISL_292923
EPI_ISL_245021	EPI_ISL_258923	EPI_ISL_253882	EPI_ISL_285972	EPI_ISL_310246	**Season 2018-19**
EPI_ISL_274119	EPI_ISL_261640	EPI_ISL_253890	EPI_ISL_292921	EPI_ISL_314681	EPI_ISL_334183
EPI_ISL_241939	EPI_ISL_241938	EPI_ISL_253952	EPI_ISL_292040	EPI_ISL_302175	EPI_ISL_334184
EPI_ISL_241901	EPI_ISL_253881	EPI_ISL_253957	EPI_ISL_285970	EPI_ISL_310248	EPI_ISL_338772
EPI_ISL_241903	EPI_ISL_258825	EPI_ISL_261658	EPI_ISL_302182	EPI_ISL_302171	EPI_ISL_338773
EPI_ISL_241910	EPI_ISL_253884	EPI_ISL_274107	EPI_ISL_307087	EPI_ISL_302289	EPI_ISL_343371
EPI_ISL_241919	EPI_ISL_239545	EPI_ISL_239543	EPI_ISL_302180	EPI_ISL_302172	EPI_ISL_355226
EPI_ISL_241928	EPI_ISL_258912	EPI_ISL_239540	EPI_ISL_302267	EPI_ISL_314685	EPI_ISL_367772
EPI_ISL_241941	EPI_ISL_241913	EPI_ISL_241917	EPI_ISL_307073	EPI_ISL_314058	EPI_ISL_343372
EPI_ISL_245015	EPI_ISL_258821	EPI_ISL_257399	EPI_ISL_281231	EPI_ISL_302277	EPI_ISL_334187
EPI_ISL_245022	EPI_ISL_241918	EPI_ISL_239534	EPI_ISL_310259	EPI_ISL_302170	EPI_ISL_334198
EPI_ISL_253860	EPI_ISL_253118	EPI_ISL_258830	EPI_ISL_282972	EPI_ISL_314663	
EPI_ISL_253883	EPI_ISL_237514	EPI_ISL_242304	EPI_ISL_281229	EPI_ISL_292906	

EPI_ISL_343373
EPI_ISL_355220
EPI_ISL_340377
EPI_ISL_334205
EPI_ISL_336165
EPI_ISL_356841
EPI_ISL_334191
EPI_ISL_334193
EPI_ISL_340384
EPI_ISL_355217
EPI_ISL_334194
EPI_ISL_336147
EPI_ISL_338755
EPI_ISL_334209
EPI_ISL_391067
EPI_ISL_355224
EPI_ISL_347810
EPI_ISL_355237
EPI_ISL_356854
EPI_ISL_347806
EPI_ISL_356863
EPI_ISL_356864
EPI_ISL_347815
EPI_ISL_355241
EPI_ISL_347816
EPI_ISL_356862
EPI_ISL_367773
EPI_ISL_356850
EPI_ISL_340378
EPI_ISL_343374
EPI_ISL_343381
EPI_ISL_347802
EPI_ISL_347812
EPI_ISL_355218
EPI_ISL_355235
EPI_ISL_355239
EPI_ISL_356839
EPI_ISL_355240
EPI_ISL_356851
EPI_ISL_367754
EPI_ISL_356843
EPI_ISL_355238
EPI_ISL_335523
EPI_ISL_336152
EPI_ISL_356858
EPI_ISL_356856
EPI_ISL_356855
EPI_ISL_356857
EPI_ISL_356844
EPI_ISL_355234
EPI_ISL_355221
EPI_ISL_334368
EPI_ISL_343375
EPI_ISL_355223
EPI_ISL_355232
EPI_ISL_347814
EPI_ISL_334186
EPI_ISL_338777
EPI_ISL_347807
EPI_ISL_335541
EPI_ISL_336166
EPI_ISL_347795
EPI_ISL_347799
EPI_ISL_355229
EPI_ISL_356859
EPI_ISL_356860
EPI_ISL_356861
EPI_ISL_356865
EPI_ISL_356866
EPI_ISL_355231
EPI_ISL_356848
EPI_ISL_340376
EPI_ISL_336154
EPI_ISL_340278
EPI_ISL_343370
EPI_ISL_343380
EPI_ISL_356840
EPI_ISL_355236
EPI_ISL_356838
EPI_ISL_343376
EPI_ISL_367810
EPI_ISL_355228
EPI_ISL_355227
EPI_ISL_332334
EPI_ISL_355233
EPI_ISL_356849
EPI_ISL_334192
EPI_ISL_343368
EPI_ISL_334180
EPI_ISL_334182
EPI_ISL_334210
EPI_ISL_334211
EPI_ISL_334207
EPI_ISL_355225
EPI_ISL_329741
EPI_ISL_343377

Season 2019-20

EPI_ISL_447874
EPI_ISL_447883
EPI_ISL_426367
EPI_ISL_420886
EPI_ISL_485779
EPI_ISL_412403
EPI_ISL_426376
EPI_ISL_426377
EPI_ISL_404258
EPI_ISL_404277
EPI_ISL_404271
EPI_ISL_447879
EPI_ISL_426372
EPI_ISL_426375
EPI_ISL_447877
EPI_ISL_447869
EPI_ISL_447868
EPI_ISL_447867
EPI_ISL_413667
EPI_ISL_413668
EPI_ISL_420883
EPI_ISL_477611
EPI_ISL_485777
EPI_ISL_391079
EPI_ISL_398731
EPI_ISL_404261
EPI_ISL_404276
EPI_ISL_447885
EPI_ISL_403231
EPI_ISL_412388
EPI_ISL_404212
EPI_ISL_404213
EPI_ISL_398735
EPI_ISL_406324
EPI_ISL_398736
EPI_ISL_403225
EPI_ISL_426369
EPI_ISL_398738
EPI_ISL_404262
EPI_ISL_412399
EPI_ISL_412400
EPI_ISL_412401
EPI_ISL_447870
EPI_ISL_426373
EPI_ISL_413685
EPI_ISL_426374
EPI_ISL_447871
EPI_ISL_477528
EPI_ISL_485749
EPI_ISL_447882
EPI_ISL_398732
EPI_ISL_413661
EPI_ISL_447881
EPI_ISL_404211
EPI_ISL_447876
EPI_ISL_447878
EPI_ISL_426366
EPI_ISL_413686
EPI_ISL_477529
EPI_ISL_485750
EPI_ISL_396431
EPI_ISL_406320
EPI_ISL_426368
EPI_ISL_413673
EPI_ISL_447873
EPI_ISL_404256
EPI_ISL_404219
EPI_ISL_398730
EPI_ISL_398737
EPI_ISL_398733
EPI_ISL_398734
EPI_ISL_399975
EPI_ISL_404214
EPI_ISL_404218
EPI_ISL_447872
EPI_ISL_412394
EPI_ISL_426370
EPI_ISL_396433
EPI_ISL_406322
EPI_ISL_413689
EPI_ISL_413688
EPI_ISL_404268
EPI_ISL_413681
EPI_ISL_403224
EPI_ISL_396436
EPI_ISL_395317
EPI_ISL_396435
EPI_ISL_406323
EPI_ISL_413665
EPI_ISL_485776
EPI_ISL_413682
EPI_ISL_413654

Selected from Russia (n=1041)

Season 2015-16

EPI_ISL_232498
EPI_ISL_224574
EPI_ISL_231824
EPI_ISL_209097
EPI_ISL_230313
EPI_ISL_205852
EPI_ISL_209098
EPI_ISL_226099
EPI_ISL_226098
EPI_ISL_209092
EPI_ISL_232501
EPI_ISL_230362
EPI_ISL_230359
EPI_ISL_230363
EPI_ISL_231825
EPI_ISL_232497
EPI_ISL_232499
EPI_ISL_232500
EPI_ISL_230360
EPI_ISL_226097
EPI_ISL_230361

Season 2016-17

EPI_ISL_256425
EPI_ISL_256418
EPI_ISL_256085
EPI_ISL_273678
EPI_ISL_256134
EPI_ISL_256809
EPI_ISL_242612
EPI_ISL_242611
EPI_ISL_242609
EPI_ISL_249007
EPI_ISL_252570
EPI_ISL_242624
EPI_ISL_242664
EPI_ISL_248438
EPI_ISL_252574
EPI_ISL_256422
EPI_ISL_273654
EPI_ISL_274561
EPI_ISL_277519
EPI_ISL_280470
EPI_ISL_257319
EPI_ISL_276910
EPI_ISL_276308
EPI_ISL_274563
EPI_ISL_256411
EPI_ISL_273705
EPI_ISL_273668
EPI_ISL_274562
EPI_ISL_259690
EPI_ISL_256077
EPI_ISL_259688
EPI_ISL_259689
EPI_ISL_261861
EPI_ISL_256404
EPI_ISL_249067
EPI_ISL_259123
EPI_ISL_242643
EPI_ISL_242654
EPI_ISL_302522
EPI_ISL_276303
EPI_ISL_256086
EPI_ISL_256084
EPI_ISL_256062
EPI_ISL_256063
EPI_ISL_280471
EPI_ISL_280471
EPI_ISL_273653
EPI_ISL_277518
EPI_ISL_273680
EPI_ISL_259686
EPI_ISL_256815
EPI_ISL_256814
EPI_ISL_256424
EPI_ISL_276316
EPI_ISL_276315
EPI_ISL_256072
EPI_ISL_256064
EPI_ISL_273660
EPI_ISL_273661
EPI_ISL_249010
EPI_ISL_249011
EPI_ISL_256807
EPI_ISL_257323
EPI_ISL_273659
EPI_ISL_242636
EPI_ISL_242645
EPI_ISL_249009
EPI_ISL_253194
EPI_ISL_253208
EPI_ISL_255922
EPI_ISL_256066
EPI_ISL_256067
EPI_ISL_256073

EPI_ISL_257320	EPI_ISL_268280	EPI_ISL_256810	EPI_ISL_256812	EPI_ISL_274572	EPI_ISL_322437
EPI_ISL_257899	EPI_ISL_242593	EPI_ISL_253195	EPI_ISL_242659	EPI_ISL_274570	EPI_ISL_315881
EPI_ISL_259139	EPI_ISL_248471	EPI_ISL_256813	EPI_ISL_242650	EPI_ISL_268738	EPI_ISL_315880
EPI_ISL_277631	EPI_ISL_249014	EPI_ISL_268281	EPI_ISL_242596	EPI_ISL_256409	EPI_ISL_322475
EPI_ISL_255745	EPI_ISL_255693	EPI_ISL_239631	EPI_ISL_280469	EPI_ISL_242660	EPI_ISL_322428
EPI_ISL_256816	EPI_ISL_256421	EPI_ISL_252573	EPI_ISL_280472	EPI_ISL_242614	EPI_ISL_297737
EPI_ISL_242656	EPI_ISL_242629	EPI_ISL_403420	EPI_ISL_280472	EPI_ISL_276313	EPI_ISL_300429
EPI_ISL_256082	EPI_ISL_268734	EPI_ISL_242591	EPI_ISL_277517	EPI_ISL_276269	EPI_ISL_320645
EPI_ISL_256083	EPI_ISL_273650	EPI_ISL_257822	EPI_ISL_273652	EPI_ISL_273657	EPI_ISL_308745
EPI_ISL_280473	EPI_ISL_242658	EPI_ISL_242619	EPI_ISL_242597	EPI_ISL_242602	EPI_ISL_320288
EPI_ISL_255750	EPI_ISL_242639	EPI_ISL_242620	EPI_ISL_242598	EPI_ISL_277513	EPI_ISL_331513
EPI_ISL_255748	EPI_ISL_252572	EPI_ISL_253189	EPI_ISL_242627	EPI_ISL_242631	EPI_ISL_315939
EPI_ISL_273672	EPI_ISL_256079	EPI_ISL_256071	EPI_ISL_242635	EPI_ISL_277620	EPI_ISL_322469
EPI_ISL_276312	EPI_ISL_256405	EPI_ISL_268282	EPI_ISL_242655	EPI_ISL_277619	EPI_ISL_320325
EPI_ISL_273708	EPI_ISL_242618	EPI_ISL_259695	EPI_ISL_242662	EPI_ISL_273707	EPI_ISL_329801
EPI_ISL_242628	EPI_ISL_256074	EPI_ISL_274436	EPI_ISL_248461	EPI_ISL_276284	EPI_ISL_330160
EPI_ISL_242600	EPI_ISL_242622	EPI_ISL_274564	EPI_ISL_252571	EPI_ISL_242634	EPI_ISL_322422
EPI_ISL_239746	EPI_ISL_242606	EPI_ISL_259691	EPI_ISL_255918	EPI_ISL_249069	EPI_ISL_320326
EPI_ISL_242615	EPI_ISL_242607	EPI_ISL_268730	EPI_ISL_255919	EPI_ISL_247714	EPI_ISL_329800
EPI_ISL_255700	EPI_ISL_242613	EPI_ISL_256080	EPI_ISL_256065	EPI_ISL_242651	EPI_ISL_330161
EPI_ISL_255753	EPI_ISL_242647	EPI_ISL_242625	EPI_ISL_256407	EPI_ISL_256075	EPI_ISL_332143
EPI_ISL_257318	EPI_ISL_242648	EPI_ISL_242626	EPI_ISL_268729	EPI_ISL_256076	EPI_ISL_299057
EPI_ISL_268736	EPI_ISL_256413	EPI_ISL_242661	EPI_ISL_273666	EPI_ISL_242640	EPI_ISL_313432
EPI_ISL_302521	EPI_ISL_256414	EPI_ISL_276909	EPI_ISL_273676	EPI_ISL_242595	EPI_ISL_286932
EPI_ISL_242657	EPI_ISL_268737	EPI_ISL_248401	EPI_ISL_276302	EPI_ISL_242604	EPI_ISL_287573
EPI_ISL_255697	EPI_ISL_273702	EPI_ISL_259692	EPI_ISL_276305	EPI_ISL_242601	EPI_ISL_287574
EPI_ISL_256416	EPI_ISL_242644	EPI_ISL_242652	EPI_ISL_276913	EPI_ISL_242599	EPI_ISL_292892
EPI_ISL_255920	EPI_ISL_273701	EPI_ISL_242653	EPI_ISL_280468	EPI_ISL_242603	EPI_ISL_297717
EPI_ISL_255921	EPI_ISL_277533	EPI_ISL_262265	EPI_ISL_280472	EPI_ISL_242605	EPI_ISL_315028
EPI_ISL_255923	EPI_ISL_274569	EPI_ISL_256426	EPI_ISL_302520	EPI_ISL_256811	EPI_ISL_322439
EPI_ISL_255924	EPI_ISL_273700	EPI_ISL_242663	EPI_ISL_280472	EPI_ISL_257322	EPI_ISL_322461
EPI_ISL_268728	EPI_ISL_274573	EPI_ISL_242646	EPI_ISL_273674	EPI_ISL_276263	EPI_ISL_322435
EPI_ISL_276310	EPI_ISL_242610	EPI_ISL_242617	EPI_ISL_273656	**Season 2017-18**	EPI_ISL_322429
EPI_ISL_256406	EPI_ISL_242623	EPI_ISL_276309	EPI_ISL_277521	EPI_ISL_322473	EPI_ISL_322453
EPI_ISL_242608	EPI_ISL_268732	EPI_ISL_273703	EPI_ISL_268731	EPI_ISL_322483	EPI_ISL_331234
EPI_ISL_239745	EPI_ISL_255925	EPI_ISL_242621	EPI_ISL_256069	EPI_ISL_322447	EPI_ISL_287568
EPI_ISL_242592	EPI_ISL_259694	EPI_ISL_242632	EPI_ISL_255688	EPI_ISL_320292	EPI_ISL_300223
EPI_ISL_242594	EPI_ISL_275711	EPI_ISL_242633	EPI_ISL_280472	EPI_ISL_329836	EPI_ISL_331236
EPI_ISL_242641	EPI_ISL_256415	EPI_ISL_274437	EPI_ISL_280472	EPI_ISL_322468	EPI_ISL_320287
EPI_ISL_242665	EPI_ISL_257321	EPI_ISL_277488	EPI_ISL_274571	EPI_ISL_320286	EPI_ISL_330960
EPI_ISL_247952	EPI_ISL_242637	EPI_ISL_276314	EPI_ISL_256427	EPI_ISL_320644	EPI_ISL_322450
EPI_ISL_273662	EPI_ISL_242638	EPI_ISL_276304	EPI_ISL_273658	EPI_ISL_320646	EPI_ISL_313514
EPI_ISL_273682	EPI_ISL_249008	EPI_ISL_256417	EPI_ISL_242616	EPI_ISL_330264	EPI_ISL_322440
EPI_ISL_276306	EPI_ISL_253207	EPI_ISL_256410	EPI_ISL_256808	EPI_ISL_308986	EPI_ISL_322460
EPI_ISL_242649	EPI_ISL_259687	EPI_ISL_256412	EPI_ISL_273704	EPI_ISL_297725	EPI_ISL_322489
EPI_ISL_256423	EPI_ISL_268735	EPI_ISL_256419	EPI_ISL_277527	EPI_ISL_305010	EPI_ISL_322465
EPI_ISL_256078	EPI_ISL_262307	EPI_ISL_256408	EPI_ISL_273706	EPI_ISL_308964	EPI_ISL_330034
EPI_ISL_255690	EPI_ISL_268733	EPI_ISL_256087	EPI_ISL_274565	EPI_ISL_322427	EPI_ISL_330938
EPI_ISL_273651	EPI_ISL_273664	EPI_ISL_255696	EPI_ISL_273655	EPI_ISL_322482	EPI_ISL_315768
EPI_ISL_273699	EPI_ISL_276311	EPI_ISL_248444	EPI_ISL_277520	EPI_ISL_322488	EPI_ISL_322474
EPI_ISL_276911	EPI_ISL_274566	EPI_ISL_259693	EPI_ISL_257324	EPI_ISL_322441	EPI_ISL_313518
EPI_ISL_256081	EPI_ISL_242642	EPI_ISL_264436	EPI_ISL_242630	EPI_ISL_320638	EPI_ISL_287569
EPI_ISL_255926	EPI_ISL_256420	EPI_ISL_273698	EPI_ISL_274568	EPI_ISL_320295	EPI_ISL_322486
EPI_ISL_253198	EPI_ISL_276307	EPI_ISL_273670	EPI_ISL_276912	EPI_ISL_329843	EPI_ISL_287567

EPI_ISL_297720
EPI_ISL_303060
EPI_ISL_313797
EPI_ISL_315882
EPI_ISL_320289
EPI_ISL_322481
EPI_ISL_322961
EPI_ISL_287570
EPI_ISL_320656
EPI_ISL_320654
EPI_ISL_320655
EPI_ISL_322445
EPI_ISL_315748
EPI_ISL_287571
EPI_ISL_287572
EPI_ISL_313429
EPI_ISL_322456
EPI_ISL_299046
EPI_ISL_313428
EPI_ISL_332142
EPI_ISL_315940
EPI_ISL_308744
EPI_ISL_315749
EPI_ISL_322455
EPI_ISL_322470
EPI_ISL_322476
EPI_ISL_322471
EPI_ISL_322443
EPI_ISL_330675
EPI_ISL_330033
EPI_ISL_330889
EPI_ISL_330162
EPI_ISL_330163
EPI_ISL_322442
EPI_ISL_321448
EPI_ISL_331232
EPI_ISL_313427
EPI_ISL_320293
EPI_ISL_320296
EPI_ISL_322960
EPI_ISL_330956
EPI_ISL_320643
EPI_ISL_322454
EPI_ISL_320648
EPI_ISL_322478
EPI_ISL_313516
EPI_ISL_322466
EPI_ISL_321444
EPI_ISL_320341
EPI_ISL_320653
EPI_ISL_329837
EPI_ISL_322479
EPI_ISL_322490
EPI_ISL_320649
EPI_ISL_320647
EPI_ISL_322477
EPI_ISL_322472
EPI_ISL_322444
EPI_ISL_297733
EPI_ISL_297732
EPI_ISL_297734
EPI_ISL_297735
EPI_ISL_297736
EPI_ISL_315941
EPI_ISL_297731
EPI_ISL_322485
EPI_ISL_322451
EPI_ISL_322448
EPI_ISL_330032
EPI_ISL_331058
EPI_ISL_322463
EPI_ISL_322436
EPI_ISL_315938
EPI_ISL_322464
EPI_ISL_322449
EPI_ISL_322452
EPI_ISL_297727
EPI_ISL_297729
EPI_ISL_297730
EPI_ISL_322457
EPI_ISL_322462
EPI_ISL_322430
EPI_ISL_322433
EPI_ISL_322434
EPI_ISL_320294
EPI_ISL_329842
EPI_ISL_313521
EPI_ISL_322438
EPI_ISL_297726
EPI_ISL_297728
EPI_ISL_285603
EPI_ISL_286933
EPI_ISL_291645
EPI_ISL_292919
EPI_ISL_297718
EPI_ISL_297719
EPI_ISL_297722
EPI_ISL_297724
EPI_ISL_299907
EPI_ISL_299908
EPI_ISL_322458
EPI_ISL_322459
EPI_ISL_322467
EPI_ISL_320324
EPI_ISL_329799
EPI_ISL_331233
EPI_ISL_283155
EPI_ISL_291646
EPI_ISL_297721
EPI_ISL_308737
EPI_ISL_313430
EPI_ISL_286931
EPI_ISL_297723
EPI_ISL_313519
EPI_ISL_331231
EPI_ISL_321443
EPI_ISL_331230
EPI_ISL_322446
EPI_ISL_322423
EPI_ISL_322424
EPI_ISL_322425
EPI_ISL_331235
EPI_ISL_308743
EPI_ISL_282487

Season 2018-19

EPI_ISL_410280
EPI_ISL_410269
EPI_ISL_351694
EPI_ISL_350672
EPI_ISL_339098
EPI_ISL_339091
EPI_ISL_338309
EPI_ISL_332726
EPI_ISL_337419
EPI_ISL_339087
EPI_ISL_339261
EPI_ISL_342009
EPI_ISL_345733
EPI_ISL_365479
EPI_ISL_373370
EPI_ISL_377664
EPI_ISL_351774
EPI_ISL_373379
EPI_ISL_342028
EPI_ISL_346070
EPI_ISL_346089
EPI_ISL_351761
EPI_ISL_351782
EPI_ISL_373377
EPI_ISL_373378
EPI_ISL_332724
EPI_ISL_364373
EPI_ISL_364801
EPI_ISL_350663
EPI_ISL_364800
EPI_ISL_364345
EPI_ISL_364357
EPI_ISL_350740
EPI_ISL_350770
EPI_ISL_351773
EPI_ISL_365473
EPI_ISL_337423
EPI_ISL_364805
EPI_ISL_364344
EPI_ISL_377654
EPI_ISL_394913
EPI_ISL_337421
EPI_ISL_351771
EPI_ISL_351755
EPI_ISL_350621
EPI_ISL_337422
EPI_ISL_351767
EPI_ISL_364358
EPI_ISL_337106
EPI_ISL_364356
EPI_ISL_378025
EPI_ISL_350772
EPI_ISL_337091
EPI_ISL_337094
EPI_ISL_342206
EPI_ISL_349743
EPI_ISL_350735
EPI_ISL_377653
EPI_ISL_337104
EPI_ISL_344875
EPI_ISL_364343
EPI_ISL_364347
EPI_ISL_331622
EPI_ISL_364326
EPI_ISL_364333
EPI_ISL_339097
EPI_ISL_341966
EPI_ISL_364320
EPI_ISL_345730
EPI_ISL_350619
EPI_ISL_364785
EPI_ISL_389228
EPI_ISL_395182
EPI_ISL_350743
EPI_ISL_377657
EPI_ISL_395055
EPI_ISL_364365
EPI_ISL_388727
EPI_ISL_388722
EPI_ISL_365478
EPI_ISL_344881
EPI_ISL_344879
EPI_ISL_365474
EPI_ISL_365468
EPI_ISL_365464
EPI_ISL_378020
EPI_ISL_394912
EPI_ISL_368819
EPI_ISL_378018
EPI_ISL_368820
EPI_ISL_357868
EPI_ISL_355585
EPI_ISL_410260
EPI_ISL_350701
EPI_ISL_337101
EPI_ISL_388772
EPI_ISL_364334
EPI_ISL_365469
EPI_ISL_350686
EPI_ISL_364349
EPI_ISL_377660
EPI_ISL_410263
EPI_ISL_389226
EPI_ISL_395184
EPI_ISL_351685
EPI_ISL_364374
EPI_ISL_350615
EPI_ISL_364362
EPI_ISL_364379
EPI_ISL_364806
EPI_ISL_388747
EPI_ISL_388725
EPI_ISL_350742
EPI_ISL_344878
EPI_ISL_350733
EPI_ISL_377656
EPI_ISL_368821
EPI_ISL_337092
EPI_ISL_337097
EPI_ISL_410265
EPI_ISL_364359
EPI_ISL_364342
EPI_ISL_351762
EPI_ISL_351689
EPI_ISL_350691
EPI_ISL_350644
EPI_ISL_350623
EPI_ISL_337100
EPI_ISL_337098
EPI_ISL_337095
EPI_ISL_351758
EPI_ISL_364784
EPI_ISL_368828
EPI_ISL_344876
EPI_ISL_350688
EPI_ISL_364378
EPI_ISL_350769
EPI_ISL_364341
EPI_ISL_350690
EPI_ISL_410256
EPI_ISL_368815
EPI_ISL_365477
EPI_ISL_364369
EPI_ISL_364340
EPI_ISL_351688
EPI_ISL_337223
EPI_ISL_337088
EPI_ISL_337089
EPI_ISL_337096
EPI_ISL_337099
EPI_ISL_337111
EPI_ISL_337222
EPI_ISL_342110
EPI_ISL_342111
EPI_ISL_344873
EPI_ISL_345535
EPI_ISL_345732
EPI_ISL_346030
EPI_ISL_346031
EPI_ISL_346034
EPI_ISL_346067
EPI_ISL_346068
EPI_ISL_346069

EPI_ISL_346086
EPI_ISL_346088
EPI_ISL_349716
EPI_ISL_350609
EPI_ISL_351756
EPI_ISL_351768
EPI_ISL_362198
EPI_ISL_364360
EPI_ISL_365466
EPI_ISL_365470
EPI_ISL_365472
EPI_ISL_373372
EPI_ISL_377649
EPI_ISL_378027
EPI_ISL_380080
EPI_ISL_388721
EPI_ISL_388726
EPI_ISL_394911
EPI_ISL_364337
EPI_ISL_364318
EPI_ISL_364338
EPI_ISL_364350
EPI_ISL_364351
EPI_ISL_364352
EPI_ISL_364353
EPI_ISL_364354
EPI_ISL_332796
EPI_ISL_345534
EPI_ISL_365480
EPI_ISL_380069
EPI_ISL_350771
EPI_ISL_364361
EPI_ISL_364363
EPI_ISL_364364
EPI_ISL_357863
EPI_ISL_380079
EPI_ISL_364366
EPI_ISL_350648
EPI_ISL_344877
EPI_ISL_344883
EPI_ISL_377659
EPI_ISL_393783
EPI_ISL_389227
EPI_ISL_395180
EPI_ISL_344880
EPI_ISL_344874
EPI_ISL_346025
EPI_ISL_350614
EPI_ISL_364376
EPI_ISL_346085
EPI_ISL_346224
EPI_ISL_350610
EPI_ISL_350611
EPI_ISL_364336
EPI_ISL_364803
EPI_ISL_378028
EPI_ISL_350773
EPI_ISL_350737
EPI_ISL_350736
EPI_ISL_368810
EPI_ISL_368826
EPI_ISL_368818
EPI_ISL_364368
EPI_ISL_377662
EPI_ISL_394946
EPI_ISL_337085
EPI_ISL_388723
EPI_ISL_350744
EPI_ISL_350741
EPI_ISL_351760
EPI_ISL_342109
EPI_ISL_365463
EPI_ISL_364348
EPI_ISL_351674
EPI_ISL_350727
EPI_ISL_351675
EPI_ISL_346023
EPI_ISL_339094
EPI_ISL_337084
EPI_ISL_337083
EPI_ISL_410259
EPI_ISL_365475
EPI_ISL_364324
EPI_ISL_350739
EPI_ISL_337093
EPI_ISL_337090
EPI_ISL_337102
EPI_ISL_337107
EPI_ISL_342006
EPI_ISL_342007
EPI_ISL_342205
EPI_ISL_349776
EPI_ISL_351757
EPI_ISL_364370
EPI_ISL_388719
EPI_ISL_337087
EPI_ISL_337086
EPI_ISL_341977
EPI_ISL_351781
EPI_ISL_351770
EPI_ISL_365465
EPI_ISL_330159
EPI_ISL_331848
EPI_ISL_364322
EPI_ISL_350673
EPI_ISL_388720
EPI_ISL_332799
EPI_ISL_332723
EPI_ISL_342012
EPI_ISL_346026
EPI_ISL_350641
EPI_ISL_378023
EPI_ISL_345610
EPI_ISL_378019
EPI_ISL_337416
EPI_ISL_364346
EPI_ISL_377655
EPI_ISL_337110
EPI_ISL_350706
EPI_ISL_337418
EPI_ISL_365467
EPI_ISL_355586
EPI_ISL_377650
EPI_ISL_410290
EPI_ISL_364371
EPI_ISL_351763
EPI_ISL_350607
EPI_ISL_357864
EPI_ISL_388724
EPI_ISL_337415
EPI_ISL_338305
EPI_ISL_342005
EPI_ISL_350705
EPI_ISL_364367
EPI_ISL_364319
EPI_ISL_350608
EPI_ISL_350726
EPI_ISL_351680
EPI_ISL_350702
EPI_ISL_364323
EPI_ISL_350675
EPI_ISL_337103
EPI_ISL_337108
EPI_ISL_337417
EPI_ISL_337420
EPI_ISL_342108
EPI_ISL_351764
EPI_ISL_351769
EPI_ISL_351783
EPI_ISL_364372
EPI_ISL_365476
EPI_ISL_331623
EPI_ISL_350639
EPI_ISL_350677
EPI_ISL_364339
EPI_ISL_364355
EPI_ISL_337105
EPI_ISL_344882
EPI_ISL_332717
EPI_ISL_345729
EPI_ISL_417900
EPI_ISL_377661
EPI_ISL_337424
EPI_ISL_378026
EPI_ISL_377658
EPI_ISL_410275
EPI_ISL_364377
EPI_ISL_364332
EPI_ISL_351765
EPI_ISL_350674
EPI_ISL_410286
EPI_ISL_350616
EPI_ISL_410288
EPI_ISL_350618
EPI_ISL_368814
EPI_ISL_388743
EPI_ISL_364804
EPI_ISL_389225
EPI_ISL_395183
EPI_ISL_378024
EPI_ISL_378029
EPI_ISL_410278
EPI_ISL_395053
EPI_ISL_364331
EPI_ISL_364329
EPI_ISL_364321
EPI_ISL_351777
EPI_ISL_350638
EPI_ISL_332795
EPI_ISL_364330
EPI_ISL_350734
EPI_ISL_377663
EPI_ISL_364375
EPI_ISL_350719
EPI_ISL_350613
EPI_ISL_351779
EPI_ISL_346087
EPI_ISL_346225
EPI_ISL_350605
EPI_ISL_373373
EPI_ISL_351776
EPI_ISL_350671
EPI_ISL_346028
EPI_ISL_346033
EPI_ISL_350728
EPI_ISL_410283
EPI_ISL_351778
EPI_ISL_351775
EPI_ISL_364327
EPI_ISL_410267
EPI_ISL_351766
EPI_ISL_337425
EPI_ISL_357862
EPI_ISL_364381
EPI_ISL_410274
EPI_ISL_410271
EPI_ISL_410270
EPI_ISL_364328
EPI_ISL_364325
EPI_ISL_351759
EPI_ISL_351686
EPI_ISL_364335
EPI_ISL_410282
EPI_ISL_337109
EPI_ISL_389229
EPI_ISL_395181
EPI_ISL_351772
EPI_ISL_410281
EPI_ISL_410277
EPI_ISL_378021
EPI_ISL_410289

Season 2019-20

EPI_ISL_419384
EPI_ISL_407145
EPI_ISL_402930
EPI_ISL_407519
EPI_ISL_527239
EPI_ISL_477610
EPI_ISL_485775
EPI_ISL_429851
EPI_ISL_419114
EPI_ISL_430832
EPI_ISL_497911
EPI_ISL_471279
EPI_ISL_419082
EPI_ISL_471274
EPI_ISL_471275
EPI_ISL_405292
EPI_ISL_398129
EPI_ISL_401514
EPI_ISL_406319
EPI_ISL_419110
EPI_ISL_419113
EPI_ISL_403967
EPI_ISL_471276
EPI_ISL_402943
EPI_ISL_403727
EPI_ISL_397335
EPI_ISL_402946
EPI_ISL_403969
EPI_ISL_405291
EPI_ISL_414033
EPI_ISL_471273
EPI_ISL_404170
EPI_ISL_409366
EPI_ISL_403680
EPI_ISL_401511
EPI_ISL_401512
EPI_ISL_403965
EPI_ISL_403966
EPI_ISL_403968
EPI_ISL_403971
EPI_ISL_405289
EPI_ISL_405290
EPI_ISL_405293
EPI_ISL_407520
EPI_ISL_410037
EPI_ISL_471272
EPI_ISL_528734
EPI_ISL_393724
EPI_ISL_403964
EPI_ISL_401513
EPI_ISL_404625
EPI_ISL_419087
EPI_ISL_471285
EPI_ISL_407100
EPI_ISL_429850
EPI_ISL_471386
EPI_ISL_471288
EPI_ISL_471538
EPI_ISL_419111
EPI_ISL_414034
EPI_ISL_429202
EPI_ISL_409365
EPI_ISL_430836
EPI_ISL_402942
EPI_ISL_402952
EPI_ISL_403970
EPI_ISL_409367
EPI_ISL_429216
EPI_ISL_429849
EPI_ISL_404167
EPI_ISL_404168
EPI_ISL_404169

EPI_ISL_419383	EPI_ISL_485769	EPI_ISL_419382	EPI_ISL_471287	EPI_ISL_471281	EPI_ISL_471280
EPI_ISL_517680	EPI_ISL_419208	EPI_ISL_471537	EPI_ISL_471277	EPI_ISL_471282	EPI_ISL_471293
EPI_ISL_419086	EPI_ISL_429198	EPI_ISL_471292	EPI_ISL_471389	EPI_ISL_471283	EPI_ISL_419083
EPI_ISL_471286	EPI_ISL_429205	EPI_ISL_471290	EPI_ISL_419085	EPI_ISL_471284	EPI_ISL_419084
EPI_ISL_471388	EPI_ISL_419385	EPI_ISL_471289	EPI_ISL_419112	EPI_ISL_471384	
EPI_ISL_477602	EPI_ISL_471291	EPI_ISL_471278	EPI_ISL_419115	EPI_ISL_528732	

Dataset 4: Accession numbers of Bangladeshi strains (n=531) collected from 2009 to 2020

Accession number	Date of Collection	Accession number	Date of Collection	Accession number	Date of Collection
EPI_ISL_149693	2013-05-07	EPI_ISL_219090	2015-09-06	EPI_ISL_233460	2016-06-02
EPI_ISL_151804	2013-05-12	EPI_ISL_219091	2015-09-08	EPI_ISL_233462	2016-06-09
EPI_ISL_168105	2014-06-09	EPI_ISL_219093	2015-09-14	EPI_ISL_233463	2016-07-13
EPI_ISL_195894	2015-05-25	EPI_ISL_219094	2015-09-14	EPI_ISL_233464	2016-06-25
EPI_ISL_201264	2015-08-12	EPI_ISL_219095	2015-09-03	EPI_ISL_233465	2016-06-12
EPI_ISL_201265	2015-08-31	EPI_ISL_219096	2015-09-09	EPI_ISL_233466	2016-06-25
EPI_ISL_201266	2015-08-24	EPI_ISL_219097	2015-09-13	EPI_ISL_233467	2016-06-13
EPI_ISL_201267	2015-08-24	EPI_ISL_219098	2015-09-21	EPI_ISL_233468	2016-07-17
EPI_ISL_201268	2015-08-26	EPI_ISL_219099	2015-09-21	EPI_ISL_233472	2016-06-21
EPI_ISL_201269	2015-08-10	EPI_ISL_219100	2015-09-22	EPI_ISL_234989	2016-07-10
EPI_ISL_201270	2015-08-20	EPI_ISL_219101	2015-09-20	EPI_ISL_235533	2016-06-26
EPI_ISL_201271	2015-08-29	EPI_ISL_219102	2015-09-19	EPI_ISL_237338	2016-06-13
EPI_ISL_202074	2015-08-10	EPI_ISL_219103	2015-10-03	EPI_ISL_238609	2016-07-14
EPI_ISL_202076	2015-08-20	EPI_ISL_219104	2015-10-05	EPI_ISL_238610	2016-06-02
EPI_ISL_202083	2015-08-17	EPI_ISL_219105	2015-10-13	EPI_ISL_238625	2016-06-27
EPI_ISL_202084	2015-08-11	EPI_ISL_219106	2015-10-13	EPI_ISL_238626	2016-07-10
EPI_ISL_202108	2015-08-29	EPI_ISL_219107	2015-10-07	EPI_ISL_238649	2016-09-01
EPI_ISL_203579	2015-08-17	EPI_ISL_219108	2015-10-12	EPI_ISL_239162	2016-07-11
EPI_ISL_208575	2015-09-09	EPI_ISL_219109	2015-10-04	EPI_ISL_239163	2016-08-08
EPI_ISL_208576	2015-09-12	EPI_ISL_219110	2015-10-05	EPI_ISL_239164	2016-09-24
EPI_ISL_208578	2015-09-03	EPI_ISL_219131	2015-11-12	EPI_ISL_240304	2016-09-24
EPI_ISL_208579	2015-09-03	EPI_ISL_219132	2015-11-09	EPI_ISL_240342	2016-07-10
EPI_ISL_208580	2015-10-19	EPI_ISL_219133	2015-12-03	EPI_ISL_242687	2016-06-11
EPI_ISL_208581	2015-09-13	EPI_ISL_220271	2015-09-08	EPI_ISL_242688	2016-07-13
EPI_ISL_208586	2015-09-07	EPI_ISL_223356	2015-09-14	EPI_ISL_242689	2016-06-12
EPI_ISL_208590	2015-11-09	EPI_ISL_223357	2015-09-19	EPI_ISL_259116	2016-09-01
EPI_ISL_208595	2015-12-03	EPI_ISL_223995	2015-10-05	EPI_ISL_268374	2017-03-12
EPI_ISL_208596	2015-11-18	EPI_ISL_225033	2015-10-03	EPI_ISL_268375	2017-03-23
EPI_ISL_208610	2015-11-12	EPI_ISL_233432	2016-07-14	EPI_ISL_275771	2017-06-14
EPI_ISL_208611	2015-11-07	EPI_ISL_233435	2016-07-12	EPI_ISL_275772	2017-06-16
EPI_ISL_208617	2015-11-10	EPI_ISL_233436	2016-07-12	EPI_ISL_275773	2017-06-12
EPI_ISL_208622	2015-12-03	EPI_ISL_233440	2016-07-12	EPI_ISL_275774	2017-06-15
EPI_ISL_208631	2015-11-16	EPI_ISL_233443	2016-07-02	EPI_ISL_275775	2017-06-13
EPI_ISL_208637	2015-09-03	EPI_ISL_233444	2016-07-02	EPI_ISL_275776	2017-06-13
EPI_ISL_211677	2015-09-10	EPI_ISL_233446	2016-06-20	EPI_ISL_275777	2017-06-19
EPI_ISL_211898	2015-11-03	EPI_ISL_233448	2016-06-25	EPI_ISL_275778	2017-06-24
EPI_ISL_212959	2015-10-03	EPI_ISL_233449	2016-06-25	EPI_ISL_275779	2017-06-21
EPI_ISL_212960	2015-10-17	EPI_ISL_233450	2016-06-27	EPI_ISL_275781	2017-06-19
EPI_ISL_212961	2015-09-17	EPI_ISL_233451	2016-06-27	EPI_ISL_275782	2017-06-19
EPI_ISL_219086	2015-09-14	EPI_ISL_233452	2016-06-11	EPI_ISL_275783	2017-06-19
EPI_ISL_219087	2015-09-14	EPI_ISL_233453	2016-06-20	EPI_ISL_275784	2017-06-21
EPI_ISL_219088	2015-09-07	EPI_ISL_233454	2016-06-23	EPI_ISL_275785	2017-06-20
EPI_ISL_219089	2015-09-02	EPI_ISL_233458	2016-07-11	EPI_ISL_275786	2017-06-06

Accession number	Date of Collection
EPI_ISL_275787	2017-06-12
EPI_ISL_275788	2017-06-13
EPI_ISL_277148	2017-07-01
EPI_ISL_277149	2017-07-01
EPI_ISL_277150	2017-06-14
EPI_ISL_277151	2017-06-13
EPI_ISL_277152	2017-06-13
EPI_ISL_277153	2017-06-13
EPI_ISL_277154	2017-06-04
EPI_ISL_277155	2017-06-12
EPI_ISL_277157	2017-06-24
EPI_ISL_277158	2017-06-22
EPI_ISL_277159	2017-06-11
EPI_ISL_277160	2017-06-11
EPI_ISL_277269	2017-05-09
EPI_ISL_277270	2017-05-07
EPI_ISL_277271	2017-05-16
EPI_ISL_277272	2017-05-16
EPI_ISL_277273	2017-05-04
EPI_ISL_277298	2017-01-01
EPI_ISL_277299	2017-01-01
EPI_ISL_277300	2017-01-01
EPI_ISL_277487	2017-06-11
EPI_ISL_282880	2017-08-16
EPI_ISL_282881	2017-08-03
EPI_ISL_282882	2017-08-02
EPI_ISL_282883	2017-08-03
EPI_ISL_283318	2017-06-21
EPI_ISL_283319	2017-06-24
EPI_ISL_283320	2017-06-13
EPI_ISL_283321	2017-05-07
EPI_ISL_283322	2017-06-19
EPI_ISL_283323	2017-06-21
EPI_ISL_285881	2017-07-01
EPI_ISL_285882	2017-06-14
EPI_ISL_285883	2017-06-24
EPI_ISL_285884	2017-06-22
EPI_ISL_286017	2017-06-13
EPI_ISL_286032	2017-08-02
EPI_ISL_286060	2017-08-13
EPI_ISL_286061	2017-08-10
EPI_ISL_286068	2017-08-03
EPI_ISL_286069	2017-08-03
EPI_ISL_286118	2017-06-11
EPI_ISL_286119	2017-07-19

Accession number	Date of Collection
EPI_ISL_286120	2017-06-11
EPI_ISL_286121	2017-06-03
EPI_ISL_288498	2017-07-19
EPI_ISL_310576	2013-01-31
EPI_ISL_330248	2018-07-24
EPI_ISL_330474	2018-01-07
EPI_ISL_330476	2018-02-07
EPI_ISL_330482	2018-06-11
EPI_ISL_330483	2018-06-11
EPI_ISL_330485	2018-06-24
EPI_ISL_330488	2018-07-11
EPI_ISL_330489	2018-07-11
EPI_ISL_330490	2018-07-18
EPI_ISL_330926	2018-07-11
EPI_ISL_330927	2018-02-07
EPI_ISL_330939	2018-06-11
EPI_ISL_330940	2018-07-11
EPI_ISL_330941	2018-07-24
EPI_ISL_330942	2018-06-11
EPI_ISL_330943	2018-07-18
EPI_ISL_331045	2018-06-24
EPI_ISL_332954	2018-08-13
EPI_ISL_332958	2018-08-27
EPI_ISL_332964	2018-08-01
EPI_ISL_332965	2018-07-30
EPI_ISL_332966	2018-08-28
EPI_ISL_332978	2018-08-06
EPI_ISL_332979	2018-08-28
EPI_ISL_332980	2018-08-09
EPI_ISL_333438	2018-07-14
EPI_ISL_333439	2018-07-24
EPI_ISL_333441	2018-07-05
EPI_ISL_333457	2018-08-28
EPI_ISL_333481	2018-08-18
EPI_ISL_333853	2018-01-07
EPI_ISL_334143	2018-08-09
EPI_ISL_334144	2018-07-24
EPI_ISL_334145	2018-07-05
EPI_ISL_334147	2018-07-14
EPI_ISL_334154	2018-08-27
EPI_ISL_336710	2018-08-28
EPI_ISL_336711	2018-08-01
EPI_ISL_336712	2018-08-27
EPI_ISL_336713	2018-08-06
EPI_ISL_336714	2018-08-28

Accession number	Date of Collection
EPI_ISL_340405	2017-08-03
EPI_ISL_340421	2017-08-03
EPI_ISL_347909	2018-10-09
EPI_ISL_357821	2018-09-09
EPI_ISL_363639	2018-09-10
EPI_ISL_363640	2018-09-10
EPI_ISL_368161	2019-05-12
EPI_ISL_368171	2019-05-15
EPI_ISL_368175	2019-05-14
EPI_ISL_368185	2019-05-22
EPI_ISL_368208	2019-04-28
EPI_ISL_378055	2019-05-03
EPI_ISL_378060	2019-05-17
EPI_ISL_378062	2019-05-19
EPI_ISL_378063	2019-05-24
EPI_ISL_378064	2019-05-25
EPI_ISL_378065	2019-05-22
EPI_ISL_378066	2019-05-23
EPI_ISL_378068	2019-05-22
EPI_ISL_378069	2019-05-20
EPI_ISL_378070	2019-05-22
EPI_ISL_378071	2019-05-24
EPI_ISL_378072	2019-05-21
EPI_ISL_378074	2019-05-11
EPI_ISL_386840	2019-05-29
EPI_ISL_386863	2019-05-27
EPI_ISL_390019	2019-06-15
EPI_ISL_390031	2019-06-12
EPI_ISL_390047	2019-06-12
EPI_ISL_390048	2019-06-10
EPI_ISL_390050	2019-06-09
EPI_ISL_390051	2019-06-09
EPI_ISL_390052	2019-06-01
EPI_ISL_390053	2019-06-09
EPI_ISL_390056	2019-06-11
EPI_ISL_390057	2019-06-15
EPI_ISL_390059	2019-06-11
EPI_ISL_390060	2019-06-12
EPI_ISL_390062	2019-06-13
EPI_ISL_390064	2019-06-12
EPI_ISL_390065	2019-06-16
EPI_ISL_390066	2019-06-14
EPI_ISL_390067	2019-06-11
EPI_ISL_390068	2019-06-15
EPI_ISL_390069	2019-06-10

Accession number	Date of Collection
EPI_ISL_390071	2019-05-28
EPI_ISL_390072	2019-05-30
EPI_ISL_390073	2019-05-27
EPI_ISL_390074	2019-05-25
EPI_ISL_390075	2019-06-08
EPI_ISL_390076	2019-06-09
EPI_ISL_390077	2019-06-08
EPI_ISL_390078	2019-06-08
EPI_ISL_390079	2019-06-01
EPI_ISL_390080	2019-06-01
EPI_ISL_390081	2019-06-01
EPI_ISL_390082	2019-06-09
EPI_ISL_390083	2019-06-09
EPI_ISL_390084	2019-06-09
EPI_ISL_390085	2019-05-26
EPI_ISL_390140	2019-06-08
EPI_ISL_390141	2019-06-17
EPI_ISL_390142	2019-06-19
EPI_ISL_390143	2019-06-18
EPI_ISL_390144	2019-06-18
EPI_ISL_390145	2019-06-17
EPI_ISL_390146	2019-06-17
EPI_ISL_390147	2019-06-17
EPI_ISL_390149	2019-06-20
EPI_ISL_390150	2019-06-22
EPI_ISL_390151	2019-06-20
EPI_ISL_390152	2019-06-20
EPI_ISL_390153	2019-06-23
EPI_ISL_390154	2019-06-19
EPI_ISL_390155	2019-06-23
EPI_ISL_390156	2019-06-19
EPI_ISL_390157	2019-06-23
EPI_ISL_390158	2019-06-22
EPI_ISL_390159	2019-06-21
EPI_ISL_390160	2019-06-19
EPI_ISL_390161	2019-06-13
EPI_ISL_390162	2019-06-11
EPI_ISL_390163	2019-06-16
EPI_ISL_390164	2019-06-20
EPI_ISL_390165	2019-06-11
EPI_ISL_390166	2019-06-13
EPI_ISL_390167	2019-06-12
EPI_ISL_390168	2019-06-10
EPI_ISL_390169	2019-06-13
EPI_ISL_390170	2019-06-15

Accession number	Date of Collection
EPI_ISL_390172	2019-06-10
EPI_ISL_390173	2019-06-15
EPI_ISL_390174	2019-06-12
EPI_ISL_390175	2019-06-12
EPI_ISL_390176	2019-06-15
EPI_ISL_390179	2019-06-11
EPI_ISL_390180	2019-06-15
EPI_ISL_390181	2019-06-12
EPI_ISL_390182	2019-06-11
EPI_ISL_390183	2019-06-12
EPI_ISL_390184	2019-06-12
EPI_ISL_390185	2019-06-15
EPI_ISL_390186	2019-06-15
EPI_ISL_390187	2019-06-12
EPI_ISL_390188	2019-06-15
EPI_ISL_390189	2019-05-28
EPI_ISL_390190	2019-06-12
EPI_ISL_390191	2019-06-16
EPI_ISL_390192	2019-06-12
EPI_ISL_390193	2019-05-30
EPI_ISL_390195	2019-05-26
EPI_ISL_390198	2019-06-01
EPI_ISL_390199	2019-06-22
EPI_ISL_391130	2019-06-17
EPI_ISL_391131	2019-06-17
EPI_ISL_391132	2019-06-17
EPI_ISL_391133	2019-06-18
EPI_ISL_391134	2019-06-18
EPI_ISL_391135	2019-06-18
EPI_ISL_391136	2019-06-18
EPI_ISL_391137	2019-06-18
EPI_ISL_391138	2019-06-18
EPI_ISL_391139	2019-06-19
EPI_ISL_391140	2019-06-19
EPI_ISL_391141	2019-06-19
EPI_ISL_391142	2019-06-19
EPI_ISL_391143	2019-06-19
EPI_ISL_391144	2019-06-19
EPI_ISL_391145	2019-06-19
EPI_ISL_391146	2019-06-19
EPI_ISL_391147	2019-06-19
EPI_ISL_391148	2019-06-20
EPI_ISL_391149	2019-06-20
EPI_ISL_391150	2019-06-20
EPI_ISL_391151	2019-06-20

Accession number	Date of Collection
EPI_ISL_391152	2019-06-20
EPI_ISL_391154	2019-06-20
EPI_ISL_391155	2019-06-22
EPI_ISL_391156	2019-06-22
EPI_ISL_391157	2019-06-22
EPI_ISL_391159	2019-06-22
EPI_ISL_391161	2019-06-23
EPI_ISL_391162	2019-06-23
EPI_ISL_391163	2019-06-23
EPI_ISL_391164	2019-06-23
EPI_ISL_391165	2019-06-23
EPI_ISL_391166	2019-06-24
EPI_ISL_391167	2019-06-24
EPI_ISL_391168	2019-06-24
EPI_ISL_391169	2019-06-24
EPI_ISL_391170	2019-06-24
EPI_ISL_391171	2019-06-25
EPI_ISL_391172	2019-06-25
EPI_ISL_391173	2019-06-25
EPI_ISL_391175	2019-06-26
EPI_ISL_391177	2019-06-26
EPI_ISL_391178	2019-06-26
EPI_ISL_391179	2019-06-26
EPI_ISL_391180	2019-06-26
EPI_ISL_391181	2019-06-26
EPI_ISL_391182	2019-06-26
EPI_ISL_391183	2019-06-26
EPI_ISL_391358	2019-06-26
EPI_ISL_391359	2019-06-26
EPI_ISL_391360	2019-06-24
EPI_ISL_391363	2019-06-22
EPI_ISL_391364	2019-06-22
EPI_ISL_391365	2019-06-19
EPI_ISL_395138	2019-07-13
EPI_ISL_395139	2019-07-08
EPI_ISL_395140	2019-07-11
EPI_ISL_395141	2019-07-09
EPI_ISL_395142	2019-07-09
EPI_ISL_395143	2019-07-13
EPI_ISL_395144	2019-07-07
EPI_ISL_395145	2019-07-04
EPI_ISL_395146	2019-07-06
EPI_ISL_395147	2019-07-07
EPI_ISL_395156	2019-07-10
EPI_ISL_395157	2019-07-10

Accession number	Date of Collection	Accession number	Date of Collection	Accession number	Date of Collection
EPI_ISL_395380	2019-07-13	EPI_ISL_398345	2019-06-24	EPI_ISL_398398	2019-07-11
EPI_ISL_395381	2019-07-11	EPI_ISL_398346	2019-08-21	EPI_ISL_398399	2019-07-14
EPI_ISL_395383	2019-07-10	EPI_ISL_398347	2019-08-22	EPI_ISL_398400	2019-07-13
EPI_ISL_395384	2019-07-09	EPI_ISL_398348	2019-08-21	EPI_ISL_398401	2019-06-17
EPI_ISL_395385	2019-07-09	EPI_ISL_398349	2019-08-20	EPI_ISL_398402	2019-06-24
EPI_ISL_395386	2019-07-11	EPI_ISL_398350	2019-06-22	EPI_ISL_398403	2019-07-16
EPI_ISL_395387	2019-07-13	EPI_ISL_398351	2019-08-20	EPI_ISL_398404	2019-07-17
EPI_ISL_395389	2019-07-02	EPI_ISL_398352	2019-08-19	EPI_ISL_398405	2019-06-24
EPI_ISL_395390	2019-07-02	EPI_ISL_398353	2019-08-10	EPI_ISL_398529	2019-07-06
EPI_ISL_395392	2019-07-07	EPI_ISL_398354	2019-08-08	EPI_ISL_398530	2019-07-09
EPI_ISL_395394	2019-07-04	EPI_ISL_398355	2019-08-18	EPI_ISL_398531	2019-07-01
EPI_ISL_395395	2019-07-03	EPI_ISL_398356	2019-08-08	EPI_ISL_398775	2019-07-14
EPI_ISL_395396	2019-07-02	EPI_ISL_398357	2019-08-10	EPI_ISL_398824	2019-08-07
EPI_ISL_395397	2019-07-06	EPI_ISL_398358	2019-08-07	EPI_ISL_398839	2019-06-22
EPI_ISL_395398	2019-07-03	EPI_ISL_398359	2019-08-04	EPI_ISL_398840	2019-06-24
EPI_ISL_395399	2019-07-02	EPI_ISL_398360	2019-08-04	EPI_ISL_398841	2019-06-24
EPI_ISL_395400	2019-07-03	EPI_ISL_398362	2019-08-05	EPI_ISL_398844	2019-08-26
EPI_ISL_395401	2019-07-04	EPI_ISL_398363	2019-08-03	EPI_ISL_398845	2019-08-28
EPI_ISL_395402	2019-07-02	EPI_ISL_398364	2019-08-03	EPI_ISL_398851	2019-07-16
EPI_ISL_395403	2019-07-02	EPI_ISL_398365	2019-08-03	EPI_ISL_398852	2019-07-21
EPI_ISL_395404	2019-07-08	EPI_ISL_398366	2019-08-05	EPI_ISL_400799	2019-10-05
EPI_ISL_395406	2019-07-11	EPI_ISL_398367	2019-08-05	EPI_ISL_400801	2019-10-06
EPI_ISL_395416	2019-07-22	EPI_ISL_398368	2019-08-04	EPI_ISL_400802	2019-10-06
EPI_ISL_395417	2019-07-30	EPI_ISL_398369	2019-08-04	EPI_ISL_400819	2019-10-12
EPI_ISL_395418	2019-07-29	EPI_ISL_398370	2019-07-29	EPI_ISL_400820	2019-10-12
EPI_ISL_395419	2019-07-23	EPI_ISL_398371	2019-07-23	EPI_ISL_401016	2019-06-23
EPI_ISL_395425	2019-07-11	EPI_ISL_398372	2019-07-28	EPI_ISL_401317	2019-06-25
EPI_ISL_395447	2019-07-11	EPI_ISL_398373	2019-07-25	EPI_ISL_401321	2019-06-25
EPI_ISL_395448	2019-07-14	EPI_ISL_398374	2019-07-24	EPI_ISL_402450	2019-10-20
EPI_ISL_395449	2019-07-13	EPI_ISL_398375	2019-07-23	EPI_ISL_402452	2019-10-02
EPI_ISL_395451	2019-07-08	EPI_ISL_398376	2019-07-29	EPI_ISL_402453	2019-09-29
EPI_ISL_397170	2019-07-02	EPI_ISL_398377	2019-07-27	EPI_ISL_402454	2019-09-28
EPI_ISL_397176	2019-06-29	EPI_ISL_398378	2019-07-27	EPI_ISL_402461	2019-10-19
EPI_ISL_397224	2019-07-09	EPI_ISL_398386	2019-07-17	EPI_ISL_402462	2019-10-24
EPI_ISL_398329	2019-07-22	EPI_ISL_398388	2019-07-20	EPI_ISL_404745	2019-06-24
EPI_ISL_398330	2019-07-18	EPI_ISL_398389	2019-07-17	EPI_ISL_404753	2019-06-24
EPI_ISL_398331	2019-07-15	EPI_ISL_398390	2019-07-21	EPI_ISL_409039	2019-07-02
EPI_ISL_398332	2019-07-17	EPI_ISL_398391	2019-07-18	EPI_ISL_430749	2019-10-19
EPI_ISL_398338	2019-04-27	EPI_ISL_398392	2019-07-17	EPI_ISL_471531	2019-07-01
EPI_ISL_398339	2019-04-27	EPI_ISL_398393	2019-07-17	EPI_ISL_491803	2019-10-06
EPI_ISL_398340	2019-06-24	EPI_ISL_398394	2019-07-18	EPI_ISL_491817	2019-08-19
EPI_ISL_398342	2019-07-01	EPI_ISL_398395	2019-07-18	EPI_ISL_584002	2020-01-03
EPI_ISL_398343	2019-07-02	EPI_ISL_398396	2019-08-25	EPI_ISL_74079	2009-05-17
EPI_ISL_398344	2019-08-26	EPI_ISL_398397	2019-07-13	EPI_ISL_96082	2011-05-19

Dataset 5: Accession numbers of South-South East Asian strains used to track evolutionary rate for HA gene (Jan 2015-Dec 2020)

Selected from Bangladesh (n=700)

EPI_ISL_1296491	EPI_ISL_882844	EPI_ISL_398404	EPI_ISL_398347	EPI_ISL_395143	EPI_ISL_391154
EPI_ISL_1015492	EPI_ISL_882843	EPI_ISL_398403	EPI_ISL_398346	EPI_ISL_395142	EPI_ISL_391153
EPI_ISL_1015491	EPI_ISL_882842	EPI_ISL_398402	EPI_ISL_398341	EPI_ISL_395141	EPI_ISL_391152
EPI_ISL_1015489	EPI_ISL_882841	EPI_ISL_398401	EPI_ISL_398332	EPI_ISL_395140	EPI_ISL_391151
EPI_ISL_1015488	EPI_ISL_882840	EPI_ISL_398400	EPI_ISL_398331	EPI_ISL_395139	EPI_ISL_391150
EPI_ISL_1015487	EPI_ISL_882839	EPI_ISL_398399	EPI_ISL_398330	EPI_ISL_395138	EPI_ISL_391148
EPI_ISL_1015485	EPI_ISL_882838	EPI_ISL_398398	EPI_ISL_398329	EPI_ISL_392549	EPI_ISL_391146
EPI_ISL_1015484	EPI_ISL_882837	EPI_ISL_398397	EPI_ISL_397224	EPI_ISL_391163	EPI_ISL_391145
EPI_ISL_965042	EPI_ISL_882836	EPI_ISL_398396	EPI_ISL_397222	EPI_ISL_391365	EPI_ISL_391144
EPI_ISL_965041	EPI_ISL_882835	EPI_ISL_398395	EPI_ISL_395451	EPI_ISL_391137	EPI_ISL_391143
EPI_ISL_965040	EPI_ISL_767337	EPI_ISL_398394	EPI_ISL_395450	EPI_ISL_391147	EPI_ISL_391142
EPI_ISL_965039	EPI_ISL_767336	EPI_ISL_398393	EPI_ISL_395449	EPI_ISL_391364	EPI_ISL_391141
EPI_ISL_965038	EPI_ISL_766852	EPI_ISL_398392	EPI_ISL_395448	EPI_ISL_391149	EPI_ISL_391139
EPI_ISL_882881	EPI_ISL_717690	EPI_ISL_398391	EPI_ISL_395447	EPI_ISL_391363	EPI_ISL_391138
EPI_ISL_882880	EPI_ISL_717689	EPI_ISL_398390	EPI_ISL_395425	EPI_ISL_391362	EPI_ISL_391136
EPI_ISL_882879	EPI_ISL_717688	EPI_ISL_398389	EPI_ISL_395419	EPI_ISL_391132	EPI_ISL_391135
EPI_ISL_882878	EPI_ISL_717687	EPI_ISL_398388	EPI_ISL_395418	EPI_ISL_391155	EPI_ISL_391134
EPI_ISL_882877	EPI_ISL_717686	EPI_ISL_398386	EPI_ISL_395417	EPI_ISL_391161	EPI_ISL_391133
EPI_ISL_882876	EPI_ISL_717685	EPI_ISL_398378	EPI_ISL_395416	EPI_ISL_391140	EPI_ISL_391131
EPI_ISL_882875	EPI_ISL_717684	EPI_ISL_398377	EPI_ISL_395415	EPI_ISL_391182	EPI_ISL_391130
EPI_ISL_882874	EPI_ISL_717683	EPI_ISL_398376	EPI_ISL_395406	EPI_ISL_391361	EPI_ISL_391129
EPI_ISL_882873	EPI_ISL_717682	EPI_ISL_398375	EPI_ISL_395404	EPI_ISL_391180	EPI_ISL_390199
EPI_ISL_882871	EPI_ISL_717681	EPI_ISL_398374	EPI_ISL_395403	EPI_ISL_391360	EPI_ISL_390198
EPI_ISL_882870	EPI_ISL_717680	EPI_ISL_398373	EPI_ISL_395402	EPI_ISL_391167	EPI_ISL_390197
EPI_ISL_882869	EPI_ISL_717679	EPI_ISL_398372	EPI_ISL_395401	EPI_ISL_391359	EPI_ISL_390196
EPI_ISL_882868	EPI_ISL_717678	EPI_ISL_398371	EPI_ISL_395400	EPI_ISL_391358	EPI_ISL_390195
EPI_ISL_882867	EPI_ISL_717677	EPI_ISL_398370	EPI_ISL_395399	EPI_ISL_391171	EPI_ISL_390194
EPI_ISL_882866	EPI_ISL_717676	EPI_ISL_398369	EPI_ISL_395398	EPI_ISL_391176	EPI_ISL_390193
EPI_ISL_882865	EPI_ISL_717675	EPI_ISL_398368	EPI_ISL_395397	EPI_ISL_391183	EPI_ISL_390192
EPI_ISL_882864	EPI_ISL_717674	EPI_ISL_398367	EPI_ISL_395396	EPI_ISL_391181	EPI_ISL_390069
EPI_ISL_882863	EPI_ISL_717673	EPI_ISL_398366	EPI_ISL_395395	EPI_ISL_391179	EPI_ISL_390191
EPI_ISL_882862	EPI_ISL_584005	EPI_ISL_398365	EPI_ISL_395394	EPI_ISL_391178	EPI_ISL_390190
EPI_ISL_882861	EPI_ISL_584004	EPI_ISL_398364	EPI_ISL_395392	EPI_ISL_391177	EPI_ISL_390189
EPI_ISL_882860	EPI_ISL_584003	EPI_ISL_398363	EPI_ISL_395391	EPI_ISL_391175	EPI_ISL_390188
EPI_ISL_882859	EPI_ISL_584002	EPI_ISL_398362	EPI_ISL_395390	EPI_ISL_391174	EPI_ISL_390187
EPI_ISL_882858	EPI_ISL_402462	EPI_ISL_398361	EPI_ISL_395389	EPI_ISL_391173	EPI_ISL_390186
EPI_ISL_882857	EPI_ISL_402454	EPI_ISL_398360	EPI_ISL_395387	EPI_ISL_391172	EPI_ISL_390185
EPI_ISL_882856	EPI_ISL_402453	EPI_ISL_398359	EPI_ISL_395386	EPI_ISL_391170	EPI_ISL_390184
EPI_ISL_882855	EPI_ISL_402452	EPI_ISL_398358	EPI_ISL_395385	EPI_ISL_391169	EPI_ISL_390183
EPI_ISL_882854	EPI_ISL_402450	EPI_ISL_398357	EPI_ISL_395384	EPI_ISL_391168	EPI_ISL_390182
EPI_ISL_882853	EPI_ISL_401321	EPI_ISL_398356	EPI_ISL_395383	EPI_ISL_391166	EPI_ISL_390181
EPI_ISL_882852	EPI_ISL_401317	EPI_ISL_398355	EPI_ISL_395381	EPI_ISL_391165	EPI_ISL_390180
EPI_ISL_882851	EPI_ISL_398852	EPI_ISL_398354	EPI_ISL_395380	EPI_ISL_391164	EPI_ISL_390179
EPI_ISL_882850	EPI_ISL_398851	EPI_ISL_398353	EPI_ISL_395157	EPI_ISL_391162	EPI_ISL_390178
EPI_ISL_882849	EPI_ISL_398847	EPI_ISL_398352	EPI_ISL_395156	EPI_ISL_391160	EPI_ISL_390177
EPI_ISL_882848	EPI_ISL_398845	EPI_ISL_398351	EPI_ISL_395147	EPI_ISL_391159	EPI_ISL_390176
EPI_ISL_882847	EPI_ISL_398824	EPI_ISL_398350	EPI_ISL_395146	EPI_ISL_391158	EPI_ISL_390175
EPI_ISL_882846	EPI_ISL_398775	EPI_ISL_398349	EPI_ISL_395145	EPI_ISL_391157	EPI_ISL_390174
EPI_ISL_882845	EPI_ISL_398405	EPI_ISL_398348	EPI_ISL_395144	EPI_ISL_391156	EPI_ISL_390173

EPI_ISL_390172	EPI_ISL_390064	EPI_ISL_332980	EPI_ISL_270162	EPI_ISL_220270	EPI_ISL_208581
EPI_ISL_390171	EPI_ISL_390062	EPI_ISL_332978	EPI_ISL_268375	EPI_ISL_219133	EPI_ISL_208580
EPI_ISL_390170	EPI_ISL_390060	EPI_ISL_332954	EPI_ISL_268374	EPI_ISL_219132	EPI_ISL_208579
EPI_ISL_390169	EPI_ISL_390059	EPI_ISL_286069	EPI_ISL_259116	EPI_ISL_219131	EPI_ISL_208578
EPI_ISL_390168	EPI_ISL_390057	EPI_ISL_286068	EPI_ISL_242689	EPI_ISL_219110	EPI_ISL_208576
EPI_ISL_390167	EPI_ISL_390056	EPI_ISL_286032	EPI_ISL_242688	EPI_ISL_219109	EPI_ISL_208575
EPI_ISL_390166	EPI_ISL_390053	EPI_ISL_286017	EPI_ISL_242687	EPI_ISL_219108	EPI_ISL_203579
EPI_ISL_390165	EPI_ISL_390052	EPI_ISL_285884	EPI_ISL_240342	EPI_ISL_219107	EPI_ISL_202115
EPI_ISL_390164	EPI_ISL_390051	EPI_ISL_285883	EPI_ISL_240304	EPI_ISL_219106	EPI_ISL_202108
EPI_ISL_390163	EPI_ISL_390050	EPI_ISL_285882	EPI_ISL_239164	EPI_ISL_219105	EPI_ISL_202084
EPI_ISL_390162	EPI_ISL_390049	EPI_ISL_285881	EPI_ISL_239163	EPI_ISL_219104	EPI_ISL_202083
EPI_ISL_390161	EPI_ISL_390048	EPI_ISL_285880	EPI_ISL_239162	EPI_ISL_219103	EPI_ISL_202076
EPI_ISL_390160	EPI_ISL_390047	EPI_ISL_283323	EPI_ISL_238649	EPI_ISL_219102	EPI_ISL_202074
EPI_ISL_390159	EPI_ISL_390031	EPI_ISL_283322	EPI_ISL_238626	EPI_ISL_219101	EPI_ISL_201636
EPI_ISL_390158	EPI_ISL_390019	EPI_ISL_283320	EPI_ISL_238625	EPI_ISL_219100	EPI_ISL_201271
EPI_ISL_390157	EPI_ISL_386863	EPI_ISL_283319	EPI_ISL_238610	EPI_ISL_219099	EPI_ISL_201270
EPI_ISL_390156	EPI_ISL_386852	EPI_ISL_283318	EPI_ISL_238609	EPI_ISL_219098	EPI_ISL_201269
EPI_ISL_390155	EPI_ISL_386840	EPI_ISL_282884	EPI_ISL_237338	EPI_ISL_219097	EPI_ISL_201268
EPI_ISL_390154	EPI_ISL_386838	EPI_ISL_282883	EPI_ISL_235534	EPI_ISL_219096	EPI_ISL_201267
EPI_ISL_390153	EPI_ISL_378074	EPI_ISL_282882	EPI_ISL_235533	EPI_ISL_219095	EPI_ISL_201266
EPI_ISL_390152	EPI_ISL_378072	EPI_ISL_282881	EPI_ISL_234989	EPI_ISL_219094	EPI_ISL_201265
EPI_ISL_390151	EPI_ISL_378071	EPI_ISL_282880	EPI_ISL_233725	EPI_ISL_219093	EPI_ISL_201264
EPI_ISL_390150	EPI_ISL_378070	EPI_ISL_277846	EPI_ISL_233472	EPI_ISL_219092	EPI_ISL_201263
EPI_ISL_390149	EPI_ISL_378069	EPI_ISL_277487	EPI_ISL_233468	EPI_ISL_219091	EPI_ISL_195894
EPI_ISL_390148	EPI_ISL_378068	EPI_ISL_277160	EPI_ISL_233467	EPI_ISL_219090	EPI_ISL_968670
EPI_ISL_390147	EPI_ISL_378066	EPI_ISL_277159	EPI_ISL_233466	EPI_ISL_219089	EPI_ISL_968668
EPI_ISL_390146	EPI_ISL_378065	EPI_ISL_277158	EPI_ISL_233465	EPI_ISL_219088	EPI_ISL_968666
EPI_ISL_390145	EPI_ISL_378064	EPI_ISL_277157	EPI_ISL_233464	EPI_ISL_219087	EPI_ISL_968664
EPI_ISL_390144	EPI_ISL_378063	EPI_ISL_277156	EPI_ISL_233463	EPI_ISL_219086	EPI_ISL_1064128
EPI_ISL_390143	EPI_ISL_378062	EPI_ISL_277155	EPI_ISL_233462	EPI_ISL_214521	EPI_ISL_1064125
EPI_ISL_390142	EPI_ISL_378060	EPI_ISL_277154	EPI_ISL_233460	EPI_ISL_214520	EPI_ISL_965036
EPI_ISL_390141	EPI_ISL_378055	EPI_ISL_277153	EPI_ISL_233458	EPI_ISL_212961	EPI_ISL_882834
EPI_ISL_390140	EPI_ISL_368208	EPI_ISL_277152	EPI_ISL_233454	EPI_ISL_212960	EPI_ISL_882833
EPI_ISL_390086	EPI_ISL_368185	EPI_ISL_277151	EPI_ISL_233453	EPI_ISL_212959	EPI_ISL_882832
EPI_ISL_390085	EPI_ISL_368175	EPI_ISL_277150	EPI_ISL_233452	EPI_ISL_211898	EPI_ISL_882831
EPI_ISL_390084	EPI_ISL_368171	EPI_ISL_277149	EPI_ISL_233451	EPI_ISL_211677	EPI_ISL_882830
EPI_ISL_390083	EPI_ISL_368161	EPI_ISL_277148	EPI_ISL_233450	EPI_ISL_208637	EPI_ISL_882829
EPI_ISL_390082	EPI_ISL_363640	EPI_ISL_275788	EPI_ISL_233449	EPI_ISL_208631	EPI_ISL_882828
EPI_ISL_390081	EPI_ISL_363639	EPI_ISL_275787	EPI_ISL_233448	EPI_ISL_208622	EPI_ISL_882827
EPI_ISL_390080	EPI_ISL_353448	EPI_ISL_275786	EPI_ISL_233446	EPI_ISL_208617	EPI_ISL_882826
EPI_ISL_390079	EPI_ISL_349919	EPI_ISL_275785	EPI_ISL_233444	EPI_ISL_208611	EPI_ISL_882825
EPI_ISL_390078	EPI_ISL_340421	EPI_ISL_275784	EPI_ISL_233443	EPI_ISL_208610	EPI_ISL_882824
EPI_ISL_390077	EPI_ISL_340405	EPI_ISL_275783	EPI_ISL_233440	EPI_ISL_208596	EPI_ISL_882823
EPI_ISL_390076	EPI_ISL_336713	EPI_ISL_275782	EPI_ISL_233436	EPI_ISL_208595	EPI_ISL_882822
EPI_ISL_390075	EPI_ISL_334147	EPI_ISL_275781	EPI_ISL_233435	EPI_ISL_208590	EPI_ISL_882821
EPI_ISL_390074	EPI_ISL_334145	EPI_ISL_275779	EPI_ISL_233432	EPI_ISL_208586	EPI_ISL_882820
EPI_ISL_390073	EPI_ISL_334144	EPI_ISL_275778	EPI_ISL_225033		
EPI_ISL_390072	EPI_ISL_334143	EPI_ISL_275777	EPI_ISL_223995		
EPI_ISL_390071	EPI_ISL_333481	EPI_ISL_275776	EPI_ISL_223357		
EPI_ISL_390070	EPI_ISL_333480	EPI_ISL_275775	EPI_ISL_223356		
EPI_ISL_390068	EPI_ISL_333442	EPI_ISL_275774	EPI_ISL_223355		
EPI_ISL_390067	EPI_ISL_333441	EPI_ISL_275773	EPI_ISL_220281		
EPI_ISL_390066	EPI_ISL_333439	EPI_ISL_275772	EPI_ISL_220271		
EPI_ISL_390065	EPI_ISL_333438	EPI_ISL_275771			

EPI_ISL_882819	EPI_ISL_409039	EPI_ISL_398345	EPI_ISL_336710	EPI_ISL_330926	EPI_ISL_283321
EPI_ISL_882818	EPI_ISL_404753	EPI_ISL_398344	EPI_ISL_334154	EPI_ISL_330490	EPI_ISL_277300
EPI_ISL_882817	EPI_ISL_404745	EPI_ISL_398343	EPI_ISL_334101	EPI_ISL_330489	EPI_ISL_277299
EPI_ISL_882816	EPI_ISL_402461	EPI_ISL_398342	EPI_ISL_333853	EPI_ISL_330488	EPI_ISL_277298
EPI_ISL_882815	EPI_ISL_401016	EPI_ISL_398340	EPI_ISL_333457	EPI_ISL_330485	EPI_ISL_277273
EPI_ISL_882814	EPI_ISL_400820	EPI_ISL_398339	EPI_ISL_332979	EPI_ISL_330483	EPI_ISL_277272
EPI_ISL_882813	EPI_ISL_400819	EPI_ISL_398338	EPI_ISL_332966	EPI_ISL_330482	EPI_ISL_277271
EPI_ISL_882812	EPI_ISL_400802	EPI_ISL_397176	EPI_ISL_332965	EPI_ISL_330476	EPI_ISL_277270
EPI_ISL_882811	EPI_ISL_400801	EPI_ISL_397175	EPI_ISL_332964	EPI_ISL_330474	EPI_ISL_277269
EPI_ISL_882810	EPI_ISL_400799	EPI_ISL_397173	EPI_ISL_332958	EPI_ISL_330248	EPI_ISL_968773
EPI_ISL_882809	EPI_ISL_398844	EPI_ISL_397170	EPI_ISL_331045	EPI_ISL_288498	EPI_ISL_959654
EPI_ISL_491819	EPI_ISL_398841	EPI_ISL_397169	EPI_ISL_330943	EPI_ISL_286121	EPI_ISL_959653
EPI_ISL_491817	EPI_ISL_398840	EPI_ISL_357821	EPI_ISL_330942	EPI_ISL_286120	EPI_ISL_959652
EPI_ISL_491816	EPI_ISL_398839	EPI_ISL_347909	EPI_ISL_330941	EPI_ISL_286119	EPI_ISL_959651
EPI_ISL_491803	EPI_ISL_398531	EPI_ISL_336714	EPI_ISL_330940	EPI_ISL_286118	
EPI_ISL_471531	EPI_ISL_398530	EPI_ISL_336712	EPI_ISL_330939	EPI_ISL_286061	
EPI_ISL_430749	EPI_ISL_398529	EPI_ISL_336711	EPI_ISL_330927	EPI_ISL_286060	

Selected from India (n=139)

EPI_ISL_506029	EPI_ISL_370685	EPI_ISL_365324	EPI_ISL_1242039	EPI_ISL_404741	EPI_ISL_296431
EPI_ISL_506028	EPI_ISL_370684	EPI_ISL_365323	EPI_ISL_1242033	EPI_ISL_404740	EPI_ISL_296429
EPI_ISL_506027	EPI_ISL_370683	EPI_ISL_365322	EPI_ISL_534009	EPI_ISL_391120	EPI_ISL_296427
EPI_ISL_506026	EPI_ISL_370682	EPI_ISL_365321	EPI_ISL_412107	EPI_ISL_391118	EPI_ISL_296425
EPI_ISL_506025	EPI_ISL_370681	EPI_ISL_365320	EPI_ISL_389146	EPI_ISL_333866	EPI_ISL_296074
EPI_ISL_506024	EPI_ISL_370680	EPI_ISL_365319	EPI_ISL_389144	EPI_ISL_331516	EPI_ISL_296073
EPI_ISL_506023	EPI_ISL_370679	EPI_ISL_365318	EPI_ISL_389143	EPI_ISL_331515	EPI_ISL_296072
EPI_ISL_506022	EPI_ISL_370678	EPI_ISL_365317	EPI_ISL_389142	EPI_ISL_331514	EPI_ISL_296071
EPI_ISL_506021	EPI_ISL_370677	EPI_ISL_365316	EPI_ISL_389141	EPI_ISL_331511	EPI_ISL_296070
EPI_ISL_506020	EPI_ISL_368315	EPI_ISL_365315	EPI_ISL_389140	EPI_ISL_329833	EPI_ISL_296069
EPI_ISL_506019	EPI_ISL_365338	EPI_ISL_365314	EPI_ISL_389139	EPI_ISL_321889	EPI_ISL_296068
EPI_ISL_506018	EPI_ISL_365337	EPI_ISL_365313	EPI_ISL_389138	EPI_ISL_321888	EPI_ISL_244853
EPI_ISL_506017	EPI_ISL_365336	EPI_ISL_365312	EPI_ISL_389137	EPI_ISL_321887	EPI_ISL_244850
EPI_ISL_506016	EPI_ISL_365335	EPI_ISL_365311	EPI_ISL_389136	EPI_ISL_321886	EPI_ISL_239657
EPI_ISL_506015	EPI_ISL_365334	EPI_ISL_365310	EPI_ISL_389134	EPI_ISL_321253	EPI_ISL_234046
EPI_ISL_506014	EPI_ISL_365333	EPI_ISL_365309	EPI_ISL_389133	EPI_ISL_321252	EPI_ISL_233426
EPI_ISL_378290	EPI_ISL_365332	EPI_ISL_365308	EPI_ISL_389132	EPI_ISL_315777	EPI_ISL_233425
EPI_ISL_370692	EPI_ISL_365331	EPI_ISL_365307	EPI_ISL_389131	EPI_ISL_314391	EPI_ISL_212992
EPI_ISL_370691	EPI_ISL_365330	EPI_ISL_365306	EPI_ISL_389130	EPI_ISL_314388	EPI_ISL_212991
EPI_ISL_370690	EPI_ISL_365329	EPI_ISL_365305	EPI_ISL_4373474	EPI_ISL_311653	
EPI_ISL_370689	EPI_ISL_365328	EPI_ISL_365304	EPI_ISL_4080564	EPI_ISL_296437	
EPI_ISL_370688	EPI_ISL_365327	EPI_ISL_365303	EPI_ISL_1015486	EPI_ISL_296435	
EPI_ISL_370687	EPI_ISL_365326	EPI_ISL_365302	EPI_ISL_965037	EPI_ISL_296434	
EPI_ISL_370686	EPI_ISL_365325	EPI_ISL_365301	EPI_ISL_404743	EPI_ISL_296433	

Selected from Singapore (n=680)

EPI_ISL_240801	EPI_ISL_240797	EPI_ISL_240793	EPI_ISL_240789	EPI_ISL_240785	EPI_ISL_240781
EPI_ISL_240800	EPI_ISL_240796	EPI_ISL_240792	EPI_ISL_240788	EPI_ISL_240784	EPI_ISL_240780
EPI_ISL_240799	EPI_ISL_240795	EPI_ISL_240791	EPI_ISL_240787	EPI_ISL_240783	EPI_ISL_240779
EPI_ISL_240798	EPI_ISL_240794	EPI_ISL_240790	EPI_ISL_240786	EPI_ISL_240782	EPI_ISL_240778

EPI_ISL_240777	EPI_ISL_239249	EPI_ISL_481340	EPI_ISL_400295	EPI_ISL_391594	EPI_ISL_350566
EPI_ISL_240776	EPI_ISL_239143	EPI_ISL_481339	EPI_ISL_400294	EPI_ISL_391593	EPI_ISL_350565
EPI_ISL_240775	EPI_ISL_239142	EPI_ISL_481338	EPI_ISL_400293	EPI_ISL_391592	EPI_ISL_350563
EPI_ISL_240774	EPI_ISL_239141	EPI_ISL_481337	EPI_ISL_400292	EPI_ISL_391591	EPI_ISL_350560
EPI_ISL_240773	EPI_ISL_239140	EPI_ISL_481336	EPI_ISL_400291	EPI_ISL_391590	EPI_ISL_350559
EPI_ISL_240772	EPI_ISL_239139	EPI_ISL_481335	EPI_ISL_399036	EPI_ISL_391588	EPI_ISL_350558
EPI_ISL_240771	EPI_ISL_239138	EPI_ISL_481334	EPI_ISL_399035	EPI_ISL_391587	EPI_ISL_350557
EPI_ISL_240770	EPI_ISL_239137	EPI_ISL_401505	EPI_ISL_399026	EPI_ISL_391586	EPI_ISL_350555
EPI_ISL_240769	EPI_ISL_239136	EPI_ISL_401505	EPI_ISL_399021	EPI_ISL_391585	EPI_ISL_350554
EPI_ISL_240768	EPI_ISL_239135	EPI_ISL_401504	EPI_ISL_399020	EPI_ISL_391584	EPI_ISL_346449
EPI_ISL_240767	EPI_ISL_239134	EPI_ISL_401503	EPI_ISL_398749	EPI_ISL_391583	EPI_ISL_346448
EPI_ISL_240766	EPI_ISL_239132	EPI_ISL_401502	EPI_ISL_398748	EPI_ISL_391582	EPI_ISL_346447
EPI_ISL_240765	EPI_ISL_239131	EPI_ISL_401501	EPI_ISL_398747	EPI_ISL_391581	EPI_ISL_346446
EPI_ISL_239328	EPI_ISL_239130	EPI_ISL_401500	EPI_ISL_398746	EPI_ISL_391580	EPI_ISL_346444
EPI_ISL_239327	EPI_ISL_239129	EPI_ISL_401499	EPI_ISL_398745	EPI_ISL_391579	EPI_ISL_346443
EPI_ISL_239326	EPI_ISL_239128	EPI_ISL_401498	EPI_ISL_398744	EPI_ISL_391577	EPI_ISL_346442
EPI_ISL_239325	EPI_ISL_239127	EPI_ISL_401497	EPI_ISL_398232	EPI_ISL_391576	EPI_ISL_346440
EPI_ISL_239323	EPI_ISL_239126	EPI_ISL_401494	EPI_ISL_398231	EPI_ISL_391575	EPI_ISL_346439
EPI_ISL_239322	EPI_ISL_239125	EPI_ISL_401491	EPI_ISL_398228	EPI_ISL_391574	EPI_ISL_346435
EPI_ISL_239321	EPI_ISL_239124	EPI_ISL_401490	EPI_ISL_398227	EPI_ISL_391573	EPI_ISL_346434
EPI_ISL_239320	EPI_ISL_239123	EPI_ISL_401489	EPI_ISL_398225	EPI_ISL_391572	EPI_ISL_346433
EPI_ISL_239319	EPI_ISL_239122	EPI_ISL_401488	EPI_ISL_398223	EPI_ISL_391571	EPI_ISL_346431
EPI_ISL_239318	EPI_ISL_239121	EPI_ISL_401487	EPI_ISL_398221	EPI_ISL_350599	EPI_ISL_346426
EPI_ISL_239317	EPI_ISL_239120	EPI_ISL_401469	EPI_ISL_398220	EPI_ISL_350598	EPI_ISL_346425
EPI_ISL_239316	EPI_ISL_239119	EPI_ISL_401468	EPI_ISL_398217	EPI_ISL_350597	EPI_ISL_346416
EPI_ISL_239315	EPI_ISL_239118	EPI_ISL_401467	EPI_ISL_398214	EPI_ISL_350596	EPI_ISL_346411
EPI_ISL_239314	EPI_ISL_239117	EPI_ISL_401466	EPI_ISL_398211	EPI_ISL_350595	EPI_ISL_346407
EPI_ISL_239313	EPI_ISL_239116	EPI_ISL_401465	EPI_ISL_398209	EPI_ISL_350594	EPI_ISL_346403
EPI_ISL_239312	EPI_ISL_239115	EPI_ISL_401464	EPI_ISL_398208	EPI_ISL_350593	EPI_ISL_346402
EPI_ISL_239311	EPI_ISL_4061316	EPI_ISL_401463	EPI_ISL_398207	EPI_ISL_350592	EPI_ISL_346401
EPI_ISL_239310	EPI_ISL_399304	EPI_ISL_401462	EPI_ISL_392653	EPI_ISL_350591	EPI_ISL_346399
EPI_ISL_239309	EPI_ISL_294421	EPI_ISL_401461	EPI_ISL_392650	EPI_ISL_350590	EPI_ISL_346398
EPI_ISL_239308	EPI_ISL_294420	EPI_ISL_401460	EPI_ISL_392648	EPI_ISL_350589	EPI_ISL_346396
EPI_ISL_239307	EPI_ISL_294419	EPI_ISL_401459	EPI_ISL_392647	EPI_ISL_350588	EPI_ISL_346395
EPI_ISL_239306	EPI_ISL_330262	EPI_ISL_401458	EPI_ISL_392643	EPI_ISL_350587	EPI_ISL_344195
EPI_ISL_239305	EPI_ISL_330025	EPI_ISL_400314	EPI_ISL_392640	EPI_ISL_350586	EPI_ISL_344194
EPI_ISL_239304	EPI_ISL_330024	EPI_ISL_400313	EPI_ISL_392639	EPI_ISL_350585	EPI_ISL_344193
EPI_ISL_239303	EPI_ISL_311692	EPI_ISL_400312	EPI_ISL_392638	EPI_ISL_350584	EPI_ISL_344192
EPI_ISL_239302	EPI_ISL_311688	EPI_ISL_400311	EPI_ISL_392636	EPI_ISL_350583	EPI_ISL_344191
EPI_ISL_239301	EPI_ISL_296168	EPI_ISL_400310	EPI_ISL_391678	EPI_ISL_350582	EPI_ISL_344190
EPI_ISL_239300	EPI_ISL_285898	EPI_ISL_400309	EPI_ISL_391677	EPI_ISL_350581	EPI_ISL_344188
EPI_ISL_239299	EPI_ISL_285897	EPI_ISL_400308	EPI_ISL_391676	EPI_ISL_350580	EPI_ISL_344187
EPI_ISL_239298	EPI_ISL_284191	EPI_ISL_400307	EPI_ISL_391675	EPI_ISL_350579	EPI_ISL_344186
EPI_ISL_239296	EPI_ISL_284136	EPI_ISL_400306	EPI_ISL_391674	EPI_ISL_350578	EPI_ISL_344185
EPI_ISL_239294	EPI_ISL_277876	EPI_ISL_400305	EPI_ISL_391673	EPI_ISL_350577	EPI_ISL_340494
EPI_ISL_239293	EPI_ISL_277146	EPI_ISL_400304	EPI_ISL_391672	EPI_ISL_350576	EPI_ISL_340493
EPI_ISL_239284	EPI_ISL_277145	EPI_ISL_400303	EPI_ISL_391671	EPI_ISL_350575	EPI_ISL_340492
EPI_ISL_239283	EPI_ISL_275710	EPI_ISL_400302	EPI_ISL_391670	EPI_ISL_350574	EPI_ISL_340491
EPI_ISL_239282	EPI_ISL_275709	EPI_ISL_400301	EPI_ISL_391669	EPI_ISL_350573	EPI_ISL_340490
EPI_ISL_239281	EPI_ISL_257001	EPI_ISL_400300	EPI_ISL_391668	EPI_ISL_350572	EPI_ISL_340489
EPI_ISL_239280	EPI_ISL_257000	EPI_ISL_400299	EPI_ISL_391667	EPI_ISL_350571	EPI_ISL_340488
EPI_ISL_239279	EPI_ISL_1655936	EPI_ISL_400298	EPI_ISL_391666	EPI_ISL_350570	EPI_ISL_340487
EPI_ISL_239251	EPI_ISL_481342	EPI_ISL_400297	EPI_ISL_391596	EPI_ISL_350569	EPI_ISL_340486
EPI_ISL_239250	EPI_ISL_481341	EPI_ISL_400296	EPI_ISL_391595	EPI_ISL_350567	EPI_ISL_340485

EPI_ISL_340484
EPI_ISL_340483
EPI_ISL_340482
EPI_ISL_340481
EPI_ISL_340480
EPI_ISL_340479
EPI_ISL_322112
EPI_ISL_322111
EPI_ISL_322110
EPI_ISL_322109
EPI_ISL_322108
EPI_ISL_322107
EPI_ISL_322106
EPI_ISL_322105
EPI_ISL_322104
EPI_ISL_322103
EPI_ISL_322102
EPI_ISL_322100
EPI_ISL_322099
EPI_ISL_322098
EPI_ISL_322097
EPI_ISL_322096
EPI_ISL_322095
EPI_ISL_322094
EPI_ISL_322093
EPI_ISL_322092
EPI_ISL_322091
EPI_ISL_322090
EPI_ISL_322089
EPI_ISL_322088
EPI_ISL_322087
EPI_ISL_322086
EPI_ISL_322085
EPI_ISL_322084
EPI_ISL_322083
EPI_ISL_322082
EPI_ISL_322081
EPI_ISL_322080
EPI_ISL_322079
EPI_ISL_322078
EPI_ISL_322077
EPI_ISL_322076
EPI_ISL_322075
EPI_ISL_322074
EPI_ISL_322073
EPI_ISL_322072
EPI_ISL_322071
EPI_ISL_322070
EPI_ISL_322069
EPI_ISL_322068
EPI_ISL_322066
EPI_ISL_322065
EPI_ISL_322064
EPI_ISL_292456
EPI_ISL_292455
EPI_ISL_292454
EPI_ISL_292453
EPI_ISL_292452
EPI_ISL_292451
EPI_ISL_292450
EPI_ISL_292449
EPI_ISL_292448
EPI_ISL_292447
EPI_ISL_292446
EPI_ISL_292445
EPI_ISL_292444
EPI_ISL_292443
EPI_ISL_292442
EPI_ISL_292441
EPI_ISL_292440
EPI_ISL_292439
EPI_ISL_288102
EPI_ISL_288101
EPI_ISL_288100
EPI_ISL_288099
EPI_ISL_288098
EPI_ISL_288097
EPI_ISL_288096
EPI_ISL_288095
EPI_ISL_288094
EPI_ISL_288093
EPI_ISL_288092
EPI_ISL_288091
EPI_ISL_288090
EPI_ISL_288089
EPI_ISL_288088
EPI_ISL_288087
EPI_ISL_288086
EPI_ISL_283101
EPI_ISL_283100
EPI_ISL_283099
EPI_ISL_283098
EPI_ISL_283096
EPI_ISL_283093
EPI_ISL_283092
EPI_ISL_283089
EPI_ISL_283085
EPI_ISL_283084
EPI_ISL_283083
EPI_ISL_283082
EPI_ISL_283081
EPI_ISL_283079
EPI_ISL_283077
EPI_ISL_283075
EPI_ISL_283072
EPI_ISL_283070
EPI_ISL_283069
EPI_ISL_283068
EPI_ISL_283067
EPI_ISL_283065
EPI_ISL_283064
EPI_ISL_283063
EPI_ISL_283062
EPI_ISL_283061
EPI_ISL_283060
EPI_ISL_283059
EPI_ISL_283058
EPI_ISL_283057
EPI_ISL_283056
EPI_ISL_283055
EPI_ISL_283054
EPI_ISL_283053
EPI_ISL_283052
EPI_ISL_283051
EPI_ISL_283050
EPI_ISL_283049
EPI_ISL_283048
EPI_ISL_283012
EPI_ISL_283011
EPI_ISL_283010
EPI_ISL_283009
EPI_ISL_283008
EPI_ISL_283007
EPI_ISL_283006
EPI_ISL_283005
EPI_ISL_283004
EPI_ISL_283003
EPI_ISL_283002
EPI_ISL_283001
EPI_ISL_269774
EPI_ISL_269773
EPI_ISL_269772
EPI_ISL_269771
EPI_ISL_269770
EPI_ISL_269769
EPI_ISL_269768
EPI_ISL_269764
EPI_ISL_269763
EPI_ISL_269762
EPI_ISL_269760
EPI_ISL_269759
EPI_ISL_269758
EPI_ISL_269757
EPI_ISL_269756
EPI_ISL_269755
EPI_ISL_269754
EPI_ISL_269753
EPI_ISL_269704
EPI_ISL_269702
EPI_ISL_269701
EPI_ISL_269700
EPI_ISL_269699
EPI_ISL_269698
EPI_ISL_255893
EPI_ISL_255588
EPI_ISL_255586
EPI_ISL_255583
EPI_ISL_255582
EPI_ISL_255581
EPI_ISL_255579
EPI_ISL_255578
EPI_ISL_255577
EPI_ISL_255576
EPI_ISL_255574
EPI_ISL_255573
EPI_ISL_255571
EPI_ISL_255570
EPI_ISL_255569
EPI_ISL_255568
EPI_ISL_255567
EPI_ISL_255566
EPI_ISL_255565
EPI_ISL_225952
EPI_ISL_225951
EPI_ISL_225949
EPI_ISL_225948
EPI_ISL_225947
EPI_ISL_225946
EPI_ISL_225945
EPI_ISL_225944
EPI_ISL_225943
EPI_ISL_225942
EPI_ISL_225941
EPI_ISL_225940
EPI_ISL_225939
EPI_ISL_225938
EPI_ISL_225937
EPI_ISL_225936
EPI_ISL_225935
EPI_ISL_225934
EPI_ISL_225933
EPI_ISL_225932
EPI_ISL_225931
EPI_ISL_225930
EPI_ISL_225929
EPI_ISL_225928
EPI_ISL_225927
EPI_ISL_225925
EPI_ISL_225924
EPI_ISL_225923
EPI_ISL_225922
EPI_ISL_225921
EPI_ISL_225920
EPI_ISL_225919
EPI_ISL_225917
EPI_ISL_225916
EPI_ISL_225915
EPI_ISL_225914
EPI_ISL_225913
EPI_ISL_225912
EPI_ISL_225911
EPI_ISL_225910
EPI_ISL_225909
EPI_ISL_225908
EPI_ISL_225907
EPI_ISL_225906
EPI_ISL_225905
EPI_ISL_225904
EPI_ISL_225903
EPI_ISL_225902
EPI_ISL_225864
EPI_ISL_225863
EPI_ISL_225862
EPI_ISL_225861
EPI_ISL_225859
EPI_ISL_225858
EPI_ISL_225857
EPI_ISL_225856
EPI_ISL_225855
EPI_ISL_225854
EPI_ISL_225853
EPI_ISL_225852
EPI_ISL_225851
EPI_ISL_225849
EPI_ISL_225845
EPI_ISL_225842
EPI_ISL_225837
EPI_ISL_225834
EPI_ISL_225830
EPI_ISL_225829
EPI_ISL_225828
EPI_ISL_225827
EPI_ISL_215842
EPI_ISL_215841
EPI_ISL_215840
EPI_ISL_215839
EPI_ISL_215838
EPI_ISL_215837
EPI_ISL_215836
EPI_ISL_215834
EPI_ISL_215833
EPI_ISL_215832
EPI_ISL_215831
EPI_ISL_215830
EPI_ISL_215829
EPI_ISL_215828
EPI_ISL_215827
EPI_ISL_215826
EPI_ISL_215715
EPI_ISL_215714
EPI_ISL_215713
EPI_ISL_215712
EPI_ISL_215711
EPI_ISL_215710
EPI_ISL_215709
EPI_ISL_215707
EPI_ISL_215706
EPI_ISL_215705
EPI_ISL_215704
EPI_ISL_215703
EPI_ISL_215702
EPI_ISL_215701
EPI_ISL_215700
EPI_ISL_215699
EPI_ISL_215698
EPI_ISL_215697
EPI_ISL_215696
EPI_ISL_215695
EPI_ISL_215694
EPI_ISL_215693
EPI_ISL_215692
EPI_ISL_215691
EPI_ISL_215690
EPI_ISL_215689
EPI_ISL_215296
EPI_ISL_215295
EPI_ISL_215294
EPI_ISL_215293
EPI_ISL_215292

EPI_ISL_215291	EPI_ISL_215285	EPI_ISL_215279	EPI_ISL_320965	EPI_ISL_291274	EPI_ISL_239803
EPI_ISL_215290	EPI_ISL_215284	EPI_ISL_215278	EPI_ISL_320964	EPI_ISL_269293	EPI_ISL_219072
EPI_ISL_215289	EPI_ISL_215283	EPI_ISL_215277	EPI_ISL_320963	EPI_ISL_269292	EPI_ISL_291290
EPI_ISL_215288	EPI_ISL_215282	EPI_ISL_545577	EPI_ISL_320960	EPI_ISL_269289	
EPI_ISL_215287	EPI_ISL_215281	EPI_ISL_534303	EPI_ISL_320959	EPI_ISL_255431	
EPI_ISL_215286	EPI_ISL_215280	EPI_ISL_514710	EPI_ISL_299646	EPI_ISL_239804	

Selected from Thailand (n=502)

EPI_ISL_252429	EPI_ISL_4069978	EPI_ISL_4069934	EPI_ISL_290127	EPI_ISL_252456	EPI_ISL_342386
EPI_ISL_252428	EPI_ISL_4069977	EPI_ISL_4069933	EPI_ISL_290126	EPI_ISL_252455	EPI_ISL_342385
EPI_ISL_252424	EPI_ISL_4069976	EPI_ISL_4069932	EPI_ISL_290125	EPI_ISL_252454	EPI_ISL_342384
EPI_ISL_252423	EPI_ISL_4069975	EPI_ISL_4069931	EPI_ISL_290124	EPI_ISL_252453	EPI_ISL_342383
EPI_ISL_252422	EPI_ISL_4069974	EPI_ISL_4069930	EPI_ISL_290123	EPI_ISL_252452	EPI_ISL_342382
EPI_ISL_4070017	EPI_ISL_4069973	EPI_ISL_4069929	EPI_ISL_290122	EPI_ISL_252451	EPI_ISL_342381
EPI_ISL_4070016	EPI_ISL_4069972	EPI_ISL_4064287	EPI_ISL_290121	EPI_ISL_252450	EPI_ISL_342380
EPI_ISL_4070015	EPI_ISL_4069971	EPI_ISL_4064286	EPI_ISL_290103	EPI_ISL_252449	EPI_ISL_342373
EPI_ISL_4070014	EPI_ISL_4069970	EPI_ISL_4064285	EPI_ISL_290102	EPI_ISL_252448	EPI_ISL_342372
EPI_ISL_4070013	EPI_ISL_4069969	EPI_ISL_4064284	EPI_ISL_290101	EPI_ISL_252447	EPI_ISL_342371
EPI_ISL_4070012	EPI_ISL_4069968	EPI_ISL_4064283	EPI_ISL_290100	EPI_ISL_252446	EPI_ISL_1367583
EPI_ISL_4070011	EPI_ISL_4069967	EPI_ISL_368346	EPI_ISL_290099	EPI_ISL_252445	EPI_ISL_1367582
EPI_ISL_4070010	EPI_ISL_4069966	EPI_ISL_368345	EPI_ISL_290098	EPI_ISL_252444	EPI_ISL_1367581
EPI_ISL_4070009	EPI_ISL_4069965	EPI_ISL_368341	EPI_ISL_290097	EPI_ISL_252443	EPI_ISL_1367580
EPI_ISL_4070008	EPI_ISL_4069964	EPI_ISL_368340	EPI_ISL_290096	EPI_ISL_252442	EPI_ISL_1367579
EPI_ISL_4070007	EPI_ISL_4069963	EPI_ISL_368339	EPI_ISL_290095	EPI_ISL_252441	EPI_ISL_1367577
EPI_ISL_4070006	EPI_ISL_4069962	EPI_ISL_368338	EPI_ISL_290094	EPI_ISL_252440	EPI_ISL_1367576
EPI_ISL_4070005	EPI_ISL_4069961	EPI_ISL_368337	EPI_ISL_290093	EPI_ISL_252439	EPI_ISL_1367575
EPI_ISL_4070004	EPI_ISL_4069960	EPI_ISL_290155	EPI_ISL_290092	EPI_ISL_252438	EPI_ISL_1367574
EPI_ISL_4070003	EPI_ISL_4069959	EPI_ISL_290154	EPI_ISL_290091	EPI_ISL_252437	EPI_ISL_1367573
EPI_ISL_4070002	EPI_ISL_4069958	EPI_ISL_290153	EPI_ISL_290090	EPI_ISL_252436	EPI_ISL_1367572
EPI_ISL_4070001	EPI_ISL_4069957	EPI_ISL_290150	EPI_ISL_290089	EPI_ISL_252435	EPI_ISL_1367570
EPI_ISL_4070000	EPI_ISL_4069956	EPI_ISL_290149	EPI_ISL_290088	EPI_ISL_252434	EPI_ISL_1367569
EPI_ISL_4069999	EPI_ISL_4069955	EPI_ISL_290148	EPI_ISL_290087	EPI_ISL_252433	EPI_ISL_1367568
EPI_ISL_4069998	EPI_ISL_4069954	EPI_ISL_290147	EPI_ISL_290086	EPI_ISL_252432	EPI_ISL_1296363
EPI_ISL_4069997	EPI_ISL_4069953	EPI_ISL_290146	EPI_ISL_290085	EPI_ISL_252431	EPI_ISL_1296344
EPI_ISL_4069996	EPI_ISL_4069952	EPI_ISL_290145	EPI_ISL_290084	EPI_ISL_252430	EPI_ISL_1296343
EPI_ISL_4069995	EPI_ISL_4069951	EPI_ISL_290144	EPI_ISL_290083	EPI_ISL_252427	EPI_ISL_1296342
EPI_ISL_4069994	EPI_ISL_4069950	EPI_ISL_290143	EPI_ISL_290082	EPI_ISL_252426	EPI_ISL_1296341
EPI_ISL_4069993	EPI_ISL_4069949	EPI_ISL_290142	EPI_ISL_290081	EPI_ISL_252425	EPI_ISL_1296340
EPI_ISL_4069992	EPI_ISL_4069948	EPI_ISL_290141	EPI_ISL_290080	EPI_ISL_403415	EPI_ISL_1296337
EPI_ISL_4069991	EPI_ISL_4069947	EPI_ISL_290140	EPI_ISL_290079	EPI_ISL_390922	EPI_ISL_1296328
EPI_ISL_4069990	EPI_ISL_4069946	EPI_ISL_290139	EPI_ISL_290078	EPI_ISL_390921	EPI_ISL_1296327
EPI_ISL_4069989	EPI_ISL_4069945	EPI_ISL_290138	EPI_ISL_290077	EPI_ISL_390920	EPI_ISL_1296326
EPI_ISL_4069988	EPI_ISL_4069944	EPI_ISL_290137	EPI_ISL_290076	EPI_ISL_390919	EPI_ISL_1296323
EPI_ISL_4069987	EPI_ISL_4069943	EPI_ISL_290136	EPI_ISL_290075	EPI_ISL_390918	EPI_ISL_1296321
EPI_ISL_4069986	EPI_ISL_4069942	EPI_ISL_290135	EPI_ISL_252464	EPI_ISL_390917	EPI_ISL_1296319
EPI_ISL_4069985	EPI_ISL_4069941	EPI_ISL_290134	EPI_ISL_252463	EPI_ISL_390916	EPI_ISL_1296318
EPI_ISL_4069984	EPI_ISL_4069940	EPI_ISL_290133	EPI_ISL_252462	EPI_ISL_390915	EPI_ISL_416794
EPI_ISL_4069983	EPI_ISL_4069939	EPI_ISL_290132	EPI_ISL_252461	EPI_ISL_390914	EPI_ISL_409064
EPI_ISL_4069982	EPI_ISL_4069938	EPI_ISL_290131	EPI_ISL_252460	EPI_ISL_390913	EPI_ISL_409062
EPI_ISL_4069981	EPI_ISL_4069937	EPI_ISL_290130	EPI_ISL_252459	EPI_ISL_390912	EPI_ISL_409061
EPI_ISL_4069980	EPI_ISL_4069936	EPI_ISL_290129	EPI_ISL_252458	EPI_ISL_390911	EPI_ISL_409060
EPI_ISL_4069979	EPI_ISL_4069935	EPI_ISL_290128	EPI_ISL_252457	EPI_ISL_342387	EPI_ISL_409059

EPI_ISL_409058	EPI_ISL_349731	EPI_ISL_329807	EPI_ISL_256131	EPI_ISL_197515	EPI_ISL_322724
EPI_ISL_409057	EPI_ISL_349719	EPI_ISL_329806	EPI_ISL_249991	EPI_ISL_197507	EPI_ISL_322723
EPI_ISL_409056	EPI_ISL_349706	EPI_ISL_329805	EPI_ISL_249990	EPI_ISL_197506	EPI_ISL_322722
EPI_ISL_409055	EPI_ISL_341176	EPI_ISL_329804	EPI_ISL_249989	EPI_ISL_197500	EPI_ISL_322721
EPI_ISL_409053	EPI_ISL_341174	EPI_ISL_329803	EPI_ISL_249988	EPI_ISL_197498	EPI_ISL_322720
EPI_ISL_409052	EPI_ISL_341171	EPI_ISL_329802	EPI_ISL_249987	EPI_ISL_197261	EPI_ISL_322719
EPI_ISL_409051	EPI_ISL_341170	EPI_ISL_329798	EPI_ISL_249986	EPI_ISL_197260	EPI_ISL_299692
EPI_ISL_409050	EPI_ISL_341169	EPI_ISL_329797	EPI_ISL_249985	EPI_ISL_197259	EPI_ISL_299691
EPI_ISL_398985	EPI_ISL_341164	EPI_ISL_305011	EPI_ISL_249984	EPI_ISL_197257	EPI_ISL_299683
EPI_ISL_398961	EPI_ISL_341163	EPI_ISL_303255	EPI_ISL_249983	EPI_ISL_718128	EPI_ISL_299682
EPI_ISL_395123	EPI_ISL_341053	EPI_ISL_303251	EPI_ISL_249982	EPI_ISL_644654	EPI_ISL_299645
EPI_ISL_395122	EPI_ISL_341052	EPI_ISL_303248	EPI_ISL_249981	EPI_ISL_644653	EPI_ISL_299644
EPI_ISL_394957	EPI_ISL_341051	EPI_ISL_303244	EPI_ISL_249980	EPI_ISL_644651	EPI_ISL_299601
EPI_ISL_394956	EPI_ISL_341050	EPI_ISL_303222	EPI_ISL_232109	EPI_ISL_644650	EPI_ISL_299600
EPI_ISL_390101	EPI_ISL_341049	EPI_ISL_303152	EPI_ISL_230294	EPI_ISL_644649	EPI_ISL_275192
EPI_ISL_390100	EPI_ISL_341048	EPI_ISL_303151	EPI_ISL_230293	EPI_ISL_583901	EPI_ISL_274948
EPI_ISL_390099	EPI_ISL_341047	EPI_ISL_303150	EPI_ISL_230292	EPI_ISL_453806	EPI_ISL_274947
EPI_ISL_386870	EPI_ISL_341046	EPI_ISL_300804	EPI_ISL_230291	EPI_ISL_453805	EPI_ISL_274946
EPI_ISL_386869	EPI_ISL_333865	EPI_ISL_300215	EPI_ISL_230290	EPI_ISL_453804	EPI_ISL_249245
EPI_ISL_386866	EPI_ISL_333864	EPI_ISL_300214	EPI_ISL_230289	EPI_ISL_419280	EPI_ISL_249244
EPI_ISL_386865	EPI_ISL_333863	EPI_ISL_299955	EPI_ISL_230288	EPI_ISL_419279	EPI_ISL_249243
EPI_ISL_386864	EPI_ISL_333862	EPI_ISL_299953	EPI_ISL_225034	EPI_ISL_410165	EPI_ISL_249242
EPI_ISL_386857	EPI_ISL_333857	EPI_ISL_299959	EPI_ISL_219116	EPI_ISL_406833	EPI_ISL_249208
EPI_ISL_386843	EPI_ISL_333854	EPI_ISL_299952	EPI_ISL_213017	EPI_ISL_406832	EPI_ISL_249190
EPI_ISL_386841	EPI_ISL_331518	EPI_ISL_277539	EPI_ISL_213016	EPI_ISL_406828	EPI_ISL_249189
EPI_ISL_386839	EPI_ISL_331077	EPI_ISL_277538	EPI_ISL_213015	EPI_ISL_406827	EPI_ISL_249188
EPI_ISL_386836	EPI_ISL_331073	EPI_ISL_277537	EPI_ISL_213014	EPI_ISL_406822	EPI_ISL_249187
EPI_ISL_386835	EPI_ISL_331072	EPI_ISL_277536	EPI_ISL_213011	EPI_ISL_406820	EPI_ISL_249156
EPI_ISL_353511	EPI_ISL_331065	EPI_ISL_277535	EPI_ISL_212972	EPI_ISL_406819	EPI_ISL_246671
EPI_ISL_350086	EPI_ISL_331064	EPI_ISL_277534	EPI_ISL_211928	EPI_ISL_405960	EPI_ISL_246670
EPI_ISL_350081	EPI_ISL_331052	EPI_ISL_277531	EPI_ISL_211923	EPI_ISL_405959	EPI_ISL_234170
EPI_ISL_350079	EPI_ISL_331051	EPI_ISL_277530	EPI_ISL_211922	EPI_ISL_339069	EPI_ISL_234160
EPI_ISL_349982	EPI_ISL_331047	EPI_ISL_277526	EPI_ISL_211921	EPI_ISL_339066	EPI_ISL_209066
EPI_ISL_349732	EPI_ISL_330902	EPI_ISL_277514	EPI_ISL_211920	EPI_ISL_323049	EPI_ISL_209065
EPI_ISL_349924	EPI_ISL_330028	EPI_ISL_277492	EPI_ISL_211900	EPI_ISL_322738	EPI_ISL_197939
EPI_ISL_349915	EPI_ISL_330027	EPI_ISL_277491	EPI_ISL_211899	EPI_ISL_322737	EPI_ISL_197936
EPI_ISL_349762	EPI_ISL_330014	EPI_ISL_277486	EPI_ISL_197529	EPI_ISL_322736	EPI_ISL_197935
EPI_ISL_349736	EPI_ISL_330012	EPI_ISL_277485	EPI_ISL_197523	EPI_ISL_322735	EPI_ISL_197934
EPI_ISL_349735	EPI_ISL_329809	EPI_ISL_277484	EPI_ISL_197520	EPI_ISL_322730	
EPI_ISL_349733	EPI_ISL_329808	EPI_ISL_256132	EPI_ISL_197518	EPI_ISL_322725	

References

1 Xia, J. *et al.* Comparative epidemiology, phylogenetics, and transmission patterns of severe influenza A/H3N2 in Australia from 2003 to 2017. *Influenza Other Respir Viruses* **14**, 700-709, doi:10.1111/irv.12772 (2020).

2 Agustiningsih, A. *et al.* Evolutionary study and phylodynamic pattern of human influenza A/H3N2 virus in Indonesia from 2008 to 2010. *PLoS One* **13**, e0201427, doi:10.1371/journal.pone.0201427 (2018).

3 Kim, J. I. *et al.* Phylogenetic relationships of the HA and NA genes between vaccine and seasonal influenza A(H3N2) strains in Korea. *PLoS One* **12**, e0172059, doi:10.1371/journal.pone.0172059 (2017).

4 Müller, N. F. *et al.* Characterising the epidemic spread of influenza A/H3N2 within a city through phylogenetics. *PLoS Pathog* **16**, e1008984, doi:10.1371/journal.ppat.1008984 (2020).

5 Al Khatib, H. A., Al Thani, A. A., Gallouzi, I. & Yassine, H. M. Epidemiological and genetic characterization of pH1N1 and H3N2 influenza viruses circulated in MENA region during 2009-2017. *BMC infectious diseases* **19**, 314, doi:10.1186/s12879-019-3930-6 (2019).

6 Adabor, E. S. Anticipating time-dependent antigenic variants of influenza A (H3N2) viruses. *Infection, genetics and evolution : journal of molecular epidemiology and evolutionary genetics in infectious diseases* **67**, 67-72, doi:10.1016/j.meegid.2018.10.028 (2019).

7 Nyang'au, E. M., Bulimo, W. D., Mobegi, V., Opanda, S. & Magiri, E. Genetic analysis of HA1 domain of influenza A/H3N2 viruses isolated in Kenya during the 2007-2013 seasons reveal significant divergence from WHO-recommended vaccine strains. *International journal of infectious diseases : IJID : official publication of the International Society for Infectious Diseases* **95**, 413-420, doi:10.1016/j.ijid.2020.04.001 (2020).

8 Westgeest, K. B. *et al.* Genomewide analysis of reassortment and evolution of human influenza A (H3N2) viruses circulating between 1968 and 2011. *Journal of virology* **88**, 2844-2857 (2014).
